# Mutation type‐specific transcriptomic signatures and readthrough therapy rescue in *SMC1A*‐related developmental and epileptic encephalopathy

**DOI:** 10.1002/epi.70150

**Published:** 2026-03-02

**Authors:** Maddalena Di Nardo, Francesca Sardina, Maria M. Pallotta, Iñigo Marcos‐Alcalde, Paulino Gómez‐Puertas, Cinzia Rinaldo, Ian D. Krantz, Antonio Musio

**Affiliations:** ^1^ Institute of Biomedical Technologies, National Research Council Pisa Italy; ^2^ Institute of Molecular Biology and Pathology, National Research Council Rome Italy; ^3^ Molecular Modeling Group, Centro de Biología Molecular Severo Ochoa, Consejo Superior de Investigaciones Científicas ‐ Universidad Autónoma de Madrid Madrid Spain; ^4^ Division of Pediatric Genetics and Genomics Cohen Children's Medical Center, Northwell Health Great Neck New York USA; ^5^ Department of Pediatrics Zucker School of Medicine, Hofstra University New York New York USA; ^6^ Present address: Institute of Molecular Genetics, National Research Council Pavia Italy

**Keywords:** ataluren, Cornelia de Lange syndrome, developmental and epileptic encephalopathy (DEE85), SMC1A, transcriptomic profiles

## Abstract

**Objective:**

This study was undertaken to investigate the molecular consequences of pathogenic variants in the *SMC1A* gene—particularly those associated with developmental and epileptic encephalopathy (DEE85)—and to evaluate the therapeutic potential of ataluren in restoring SMC1A function and mitigating disease‐related transcriptomic and genomic alterations.

**Methods:**

The study analyzed transcriptomic profiles from cell lines derived from individuals with DEE85 and Cornelia de Lange syndrome (CdLS), comparing the effects of different *SMC1A* variants. Particular focus was placed on nonsense variants and their impact on gene expression. Functional assays were conducted to assess the ability of ataluren to restore SMC1A protein expression, correct transcriptional defects, and reduce genomic instability.

**Results:**

Transcriptomic alterations were strongly dependent on variant type, with nonsense variants causing the most profound gene expression changes. DEE85 and CdLS cell lines exhibited distinct transcriptional signatures. Treatment with ataluren led to successful restoration of SMC1A protein levels, partial correction of gene expression abnormalities, and a reduction in genomic instability in cells harboring nonsense variants.

**Significance:**

These findings demonstrate that *SMC1A*‐related epileptic encephalopathies are driven by variant‐specific molecular mechanisms and highlight the therapeutic promise of ataluren for DEE85. The study supports further development of precision medicine strategies targeting nonsense variants in *SMC1A*, with potential implications for improving diagnosis, treatment, and quality of life in affected individuals.

**Key points**

- Mutation‐specific impact: nonsense variants in *SMC1A* lead to the most severe transcriptomic disruptions and genomic instability, distinguishing DEE85 from CdLS at the molecular level.
- Therapeutic potential of ataluren: treatment with ataluren restores SMC1A protein expression and partially corrects gene expression defects in cells with nonsense variants, supporting its use in precision therapies for DEE85.
- Stratified clinical relevance: comparative transcriptomic analysis reveals distinct molecular signatures between epileptic and developmental phenotypes, highlighting the need for variant‐informed diagnostic and therapeutic strategies.

## 1 INTRODUCTION

The cohesin complex is a multisubunit protein complex of four main subunits: the SMC1A and SMC3 adenosine triphosphatases (ATPases), the kleisin RAD21, and either SA1 or SA2 (STAG1 or STAG2), which link the SMC heterodimer to the DNA and regulatory factors.^1^ One of the primary functions of cohesin is to hold sister chromatids together from S phase until their separation during anaphase, a process essential for accurate chromosome segregation.^2^ It interacts with DNA by encircling chromatin fibers and is loaded onto chromatin by the NIPBL‐MAU2 complex. Although cohesin loading is genome‐wide, its stable chromatin binding and retention are highly regulated and often restricted to specific genomic locations, notably at sites co‐occupied by the architectural protein CTCF.^3^ Cohesin participates in the establishment and maintenance of topologically associating domains (TADs) and chromatin loops, which bring distal regulatory elements such as enhancers into close spatial proximity with their target promoters.^4^, ^5^ These structures help define regulatory neighborhoods and insulate genes from inappropriate enhancer activity. CTCF serves as a boundary element, and together with cohesin, forms loop anchors in a process known as loop extrusion, in which cohesin extrudes loops of chromatin until it encounters convergently oriented CTCF sites.^6^, ^7^

The cohesin complex is also a crucial guardian of genome stability, performing several interconnected functions that ensure the faithful transmission of genetic material, the repair of damaged DNA, and the preservation of chromosomal architecture. Cohesin is actively recruited to sites of DNA damage in a process dependent on the SMC‐loading factor NIPBL, independently of its canonical role in cohesion.^8^, ^9^ Cells deficient in cohesin subunits or its regulators show hypersensitivity to genotoxic stress such as ionizing radiation, mitomycin C, and replication inhibitors, underscoring cohesin's protective role during genotoxic challenges.^10^ Perturbation of cohesin function—by mutation or depletion—leads to widespread gene expression changes that often precede alterations in chromatin accessibility or TAD architecture and genome instability. Such effects are observed in both developmental diagnoses and cancer.^10^, ^11^, ^12^

Germline variants in *SMC1A* have been identified as one of the genetic causes of Cornelia de Lange syndrome (CdLS), a multisystem developmental diagnosis characterized by growth delay, intellectual disability (ID), limb differences, and distinctive facial features. Although pathogenic variants in *NIPBL* account for the majority of CdLS cases, *SMC1A* variants, missense or small in‐frame deletion, represent a significant subset, often associated with a milder or atypical phenotype.^13^, ^14^, ^15^ Recently, de novo variants in *SMC1A* have been reported in individuals with developmental and epileptic encephalopathy (DEE85), typically characterized by early onset seizures, global developmental delay, ID, and drug‐resistant epilepsy. Unlike the milder CdLS phenotypes associated with in‐frame or missense mutations, the epileptic phenotypes are frequently linked to loss‐of‐function variants such as nonsense, frameshift, or splice‐site mutations.^16^, ^17^, ^18^, ^19^, ^20^, ^21^, ^22^, ^23^ This condition significantly impacts patients' quality of life; however, because no dedicated studies have yet been conducted, there is an urgent need to investigate these patients, who often have limited therapeutic options. Understanding the molecular and clinical features of their condition could lead to earlier diagnosis, better seizure control, and ultimately the development of targeted therapies.

Ataluren is a small‐molecule drug designed to promote ribosomal readthrough of premature termination codons, which are responsible for approximately 12% of all inherited disease‐causing mutations.^24^ By enabling the translation machinery to bypass nonsense mutations, ataluren can restore the production of full‐length, functional proteins. Clinically, ataluren has shown promise in treating Duchenne muscular dystrophy (DMD) caused by nonsense mutations in the dystrophin gene. It was approved by the European Medicines Agency for ambulatory patients aged 2 years and older.^25^, ^26^ Beyond DMD, ataluren has been investigated in cystic fibrosis, aniridia, and other rare genetic disorders where nonsense mutations disrupt essential protein function.^27^

This study highlights how transcriptomic alterations caused by pathogenic *SMC1A* variants are strongly dependent on mutation type, with nonsense mutations inducing the most extensive and profound changes in gene expression. Comparative analysis between DEE85 and CdLS cell lines confirms the presence of distinct transcriptional signatures, underscoring the complexity of the molecular mechanisms underlying these clinical phenotypes. Furthermore, treatment with ataluren successfully restored SMC1A protein expression, partially corrected gene expression defects, and reduced genomic instability in cells carrying nonsense mutations. These findings support the therapeutic potential of ataluren for DEE85 and pave the way for precision medicine approaches targeting *SMC1A*‐related epileptic encephalopathies.

## 2 MATERIALS AND METHODS

### 2.1 Cell lines

Lymphocytes from 11 unrelated DEE85 girls characterized by ID and drug‐resistant epilepsy (epileptic [EP] cells) were immortalized by Epstein–Barr virus. All patients harbored variants (deletion, frameshift, nonsense, and missense) in the *SMC1A* gene. In addition, three CdLS (carrying missense variants in the *SMC1A* gene) and three healthy and normal control cell lines were used in this study (Table S1). Lymphoblastoid cell lines (LCLs) were grown in RPMI 1640 medium supplemented with 10% fetal bovine serum, 100 U/mL penicillin, .1 mg/mL streptomycin, and 1% L‐glutamine. This study was conducted according to the principles expressed in the Declaration of Helsinki. Informed consent was obtained from the families, according to the procedures established by the National Research Council Ethical Clearance (199894/2023).

### 2.2 Structural modeling of SMC1A

The structural model of the homodimer of human proteins SMC1A (UniprotKB id: Q14683) and SMC3 (UniprotKB id: Q9UQE7) was generated from the following structures present in the Protein Data Bank: 7OGT (crystal structure of the folded elbow of the yeast SMC1 protein), 7DG5 (crystal structure of mouse Smc1‐Smc3 hinge domain), and 8POA (human Cohesin ATPase module).^28^, ^29^ Four partial models were extracted from this model for molecular dynamics simulations: a head model, containing amino acids 2–169 and 1061–1227 of SMC1A, amino acids 1–162 and 1029–1215 of SMC3, as well as two ATP molecules and two Mg^++^ atoms; a hinge model, containing amino acids 479–679 of SMC1A and amino acids 468–687 of SMC3; a coiled‐coil model, containing amino acids 226–308, 444–487, 668–710, and 849–890 of SMC1A and amino acids 264–311, 450–495, 676–723, and 905–957 of SMC3, around amino acid Arg693; and a second coiled‐coil model, containing amino acids 205–249 and 908–1022 of SMC1A and amino acids 204–251 and 918–991 of SMC3, around amino acid Tyr983. The structures of the wild‐type domains were modeled by combining residue positions obtained using Phyre 2^30^ and SwissModel.^31^ The structures of the SMC1A Gly32Glu, Arg96Cys, Arg496His, Arg693Gly, and Tyr983Cys variants were modeled using the models of the wild‐type domains as templates.

#### 2.2.1 Molecular dynamics simulation of wild‐type and variant proteins

The nine structural models, four corresponding to the partial models of the wild‐type complex and five corresponding to each of the variants, were subjected to 200 ns of unrestrained molecular dynamics (MD) simulation using the Amber18 package (https://ambermd.org; University of California, San Francisco), essentially as previously described.^32^ Briefly, after solvation, initial wild‐type and variant model structures were subjected to 10 000 cycles of energy minimization, followed by a 1‐ns restrained equilibration phase in which the temperature was smoothly raised to 297 K, after which the restraints were gradually removed over 10 ns. Each system was then subjected to a 200‐ns free MD production phase. Trajectories were analyzed using cpptraj^33^ and VMD.^34^ Plots were generated using PyMOL (https://pymol.org).

### 2.3 DNA synthesis and quantitative real‐time polymerase chain reaction

Total RNA was isolated from LCLs using the RNeasy Mini Kit (Qiagen), and cDNA was synthesized with SuperScript II Reverse Transcriptase and oligo(dT) primers (Invitrogen). Quantitative polymerase chain reaction (PCR) reactions were performed in duplicate using QuantiTect SYBR Green PCR Master Mix (Qiagen) on a Rotor‐Gene 3000 system (Corbett). Gene expression levels were normalized to hypoxanthine phosphoribosyltransferase. As no significant differences were observed among control cell lines, their data were pooled. Primer sequences used for mRNA analysis are listed in Table S2. Statistical differences in gene expression among EP, CdLS, and control cell lines were assessed using Student *t*‐test.

### 2.4 Ataluren treatment

Cells were treated with .5, 1.5, or 3 μg/mL ataluren for 24 h. After treatment, either total protein was extracted for Western blot analysis or mRNA was isolated to assess gene expression profiles.

### 2.5 Statistical analysis

Differences in the number of chromosomal aberrations were assessed using Student *t*‐test. Statistical significance was defined as a *p* < .05.

## 3 RESULTS

### 3.1 Mutation‐type specific transcriptomic alterations in *SMC1A* ‐related epilepsy

**FIGURE 1:**
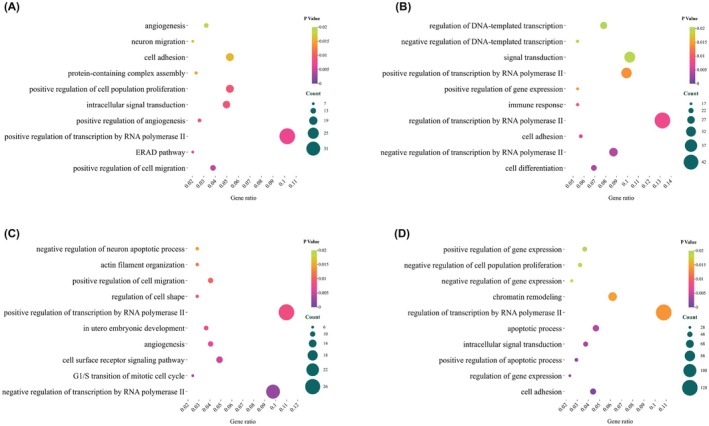
Transcriptomic profiling reveals distinct metabolic pathway alterations in epileptic (EP) cells. Metabolic pathway enrichment analysis was performed using EnrichR on differentially expressed genes (DEGs) from EP cells compared to controls. Bubble plots illustrate the enriched pathways across Gene Ontology categories for the following: (A) all EP cells, (B) EP cells carrying frameshift variants, (C) EP cells harboring missense variants, and (D) EP cells carrying nonsense variants. Each plot highlights deregulated pathways specific to the genetic subgroup, reflecting both shared and unique transcriptomic signatures. Dot size corresponds to the number of DEGs associated with each pathway, and color intensity indicates statistical significance (*p*‐value). These analyses underscore the impact of variant type on cellular metabolism and provide insights into genotype‐specific molecular mechanisms. ERAD, endoplasmic reticulum‐associated degradation.

**FIGURE 2:**
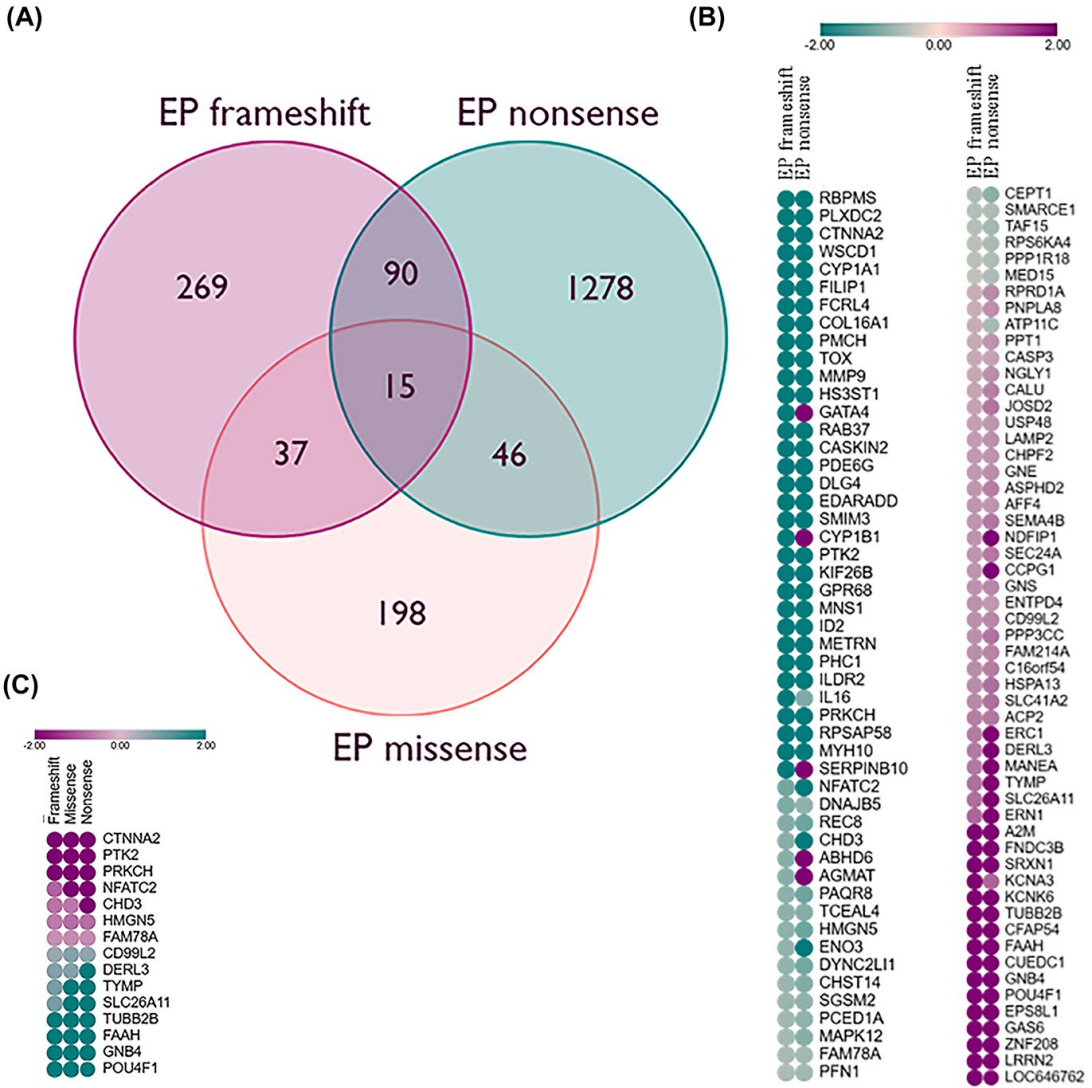
Comparative transcriptomic analysis across *SMC1A* variant groups. (A) Venn diagram illustrating the overlap of differentially expressed genes (DEGs) between nonsense and frameshift groups, revealing 105 shared genes. (B) Heatmap showing that 99 of these genes exhibit a concordant expression trend across both groups, albeit with varying magnitude, whereas six genes (*ABHD6*, *AGMAT*, *GATA4*, *ATP11C*, *CYP1B1*, and *SERPINB10*) display opposite regulation. (C) Only 15 DEGs are shared among frameshift, nonsense, and missense groups, highlighting limited transcriptomic overlap and supporting the presence of mutation‐specific expression signatures. EP, epileptic cells.

Genome‐wide transcriptional profiling was performed on 11 LCLs derived from DEE85 girls, all carrying pathogenic variants in the *SMC1A* gene (EP1–EP14). For comparison, we included three LCLs from individuals with clinically diagnosed CdLS harboring *SMC1A* variants (CdL363, CdL565, CdL060), and three control LCLs (LCL1, LCL3, LCL4). Among the EP cell lines, one carried a deletion encompassing *SMC1A*, two had nonsense variants, three had missense variants, and five carried frameshift variants (Table S1). Most variants mapped to the protein's coiled‐coil regions, with two positioned in the N‐head domain and one near the hinge region (Figure S1). *SMC1A* variants did not affect transcription (data not shown). To assess whether the protein is produced, we performed immunoblot analysis of SMC1A across all cell lines. In all cases, SMC1A levels were comparable to control cells, except in EP1 and EP2 cells carrying nonsense variants (Figure S2). Differential gene expression analysis (DGEA) comparing EP cell lines to controls identified 449 differentially expressed genes (DEGs; 284 were upregulated and 165 downregulated; Figure S3A, Table S3). Gene Ontology (GO) enrichment analysis showed involvement in angiogenesis, transcriptional regulation, cell adhesion and migration, signal transduction, and neuronal migration (Figure 1A). To assess variant‐specific effects, we performed separate DGEA comparisons for each subgroup. EP cell lines with frameshift variants showed 411 DEGs (251 upregulated and 160 downregulated) enriched mainly in pathways involved in transcriptional regulation (Figure 1B, Figure S3B, Table S4). Missense variants affected 296 genes (133 upregulated and 163 downregulated), with pathways linked to transcriptional control, angiogenesis, signal transduction, and cell migration (Figure 1C, Figure S3C, Table S5). Nonsense variants caused the most extensive changes, with 1429 misregulated genes (656 upregulated and 773 downregulated). Beyond transcriptional dysregulation, this group showed enrichment in chromatin remodeling, apoptosis, cell adhesion, cell proliferation, and intracellular signaling (Figure 1D, Figure S3D, Table S6). A comparative analysis revealed that 105 genes were shared between nonsense and frameshift groups (Figure 2A), with 99 showing concordant expression trend, albeit with variable intensity, and six genes (*ABHD6, AGMAT, GATA4*, *ATP11C, CYP1B1*, and *SERPINB10*) reversing direction (Figure 2B). Differential expression was validated by quantitative real‐time PCR (Figure S4). Only 15 genes (*CD99L2*, *CHD3*, *CTNNA2*, *DERL3*, *FAAH*, *FAM78A*, *GNB4*, *HMGN5*, *NFATC2*, *POU4F1*, *PRKCH*, *PTK2*, *SLC26A11*, *TUBB2B*, and *TYMP*) were common to all three mutation types—frameshift, nonsense, and missense (Figure 2C)—indicating limited overlap and mutation‐specific transcriptional signatures. These findings suggest that although nonsense and frameshift mutations may share some downstream effects, nonsense variants exert broader and more disruptive impacts on gene regulation. Taken together, altered gene expression appears to be a hallmark of DEE85, with nonsense variants producing the most severe transcriptional dysregulation.

### 3.2 Transcriptomic comparison of CdLS and DEE85 reveals mutation‐specific signatures

**FIGURE 3:**
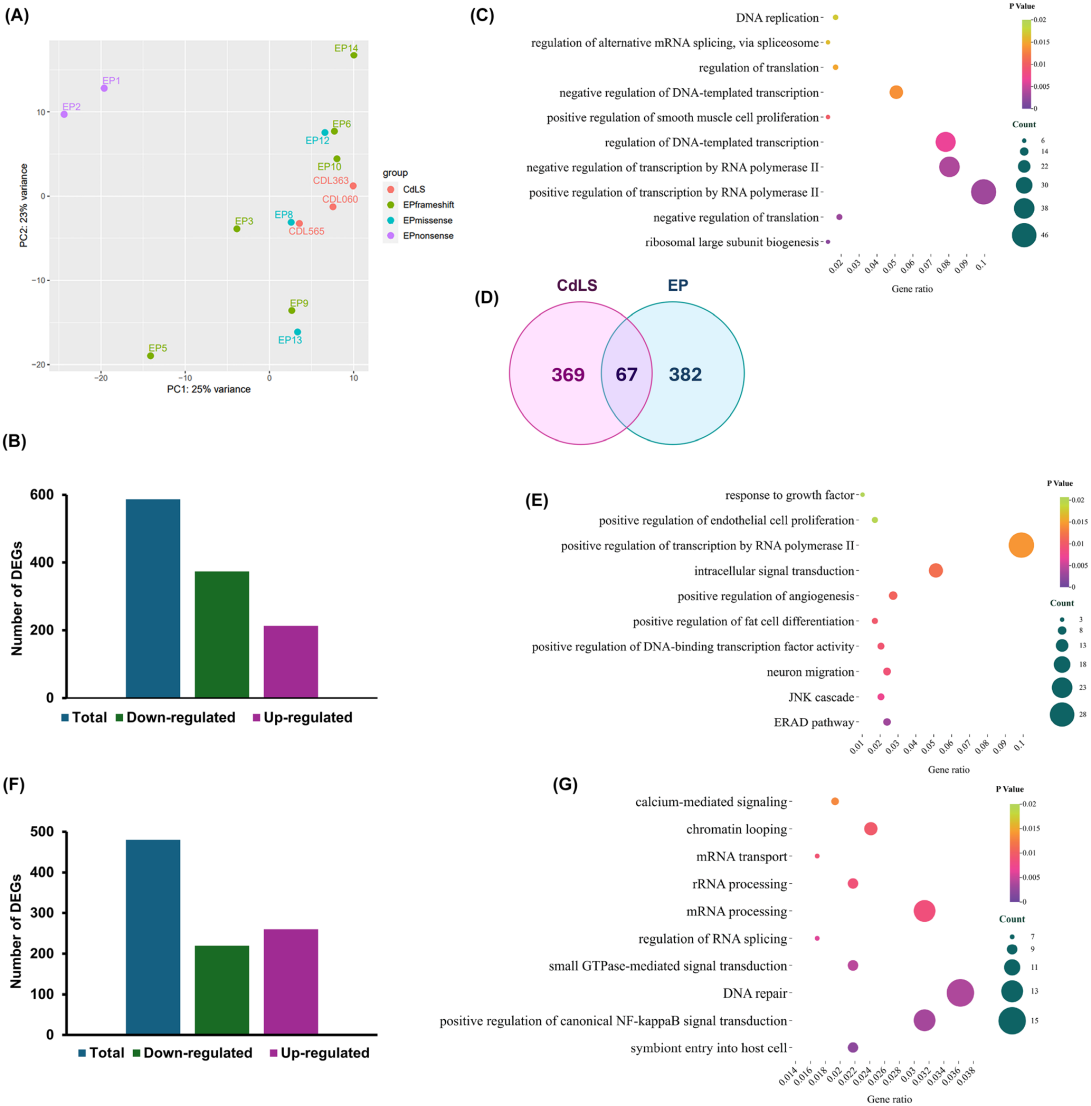
Transcriptomic divergence between Cornelia de Lange syndrome (CdLS) and developmental and epileptic encephalopathy (DEE85) highlights variant‐specific molecular signatures. (A) Principal component analysis (PCA) highlights distinct clustering of epileptic (EP) nonsense samples, clearly separated from CdLS, whereas EP frameshift and missense samples show greater dispersion, consistent with increased transcriptional heterogeneity. (B) Differential expression analysis identified 587 differentially expressed genes (DEGs) between DEE85 and CdLS (374 downregulated, 213 upregulated). (C) Bubble plot illustrates the pathways across Gene Ontology (GO)‐enriched categories enriched in pathways related to transcription, translation, DNA replication, and mRNA processing. (D) To define DEE85‐specific signatures, genes shared with CdLS and controls were excluded, yielding 382 uniquely dysregulated genes. (E) GO enrichment analysis of this DEE85‐specific gene set revealed significant involvement in transcriptional regulation, chromatin remodeling, angiogenesis, intracellular signaling, and neuronal migration. (F) A focused comparison between EP and CdLS samples carrying *SMC1A* missense variants identified 480 DEGs (220 downregulated, 260 upregulated). (G) Bubble plot showing that these DEGs are implicated in biological processes such as calcium‐mediated signaling, RNA processing, chromatin looping, and DNA repair. Dot size corresponds to the number of DEGs associated with each pathway, and color intensity indicates statistical significance (*p*‐value). ERAD, endoplasmic reticulum‐associated degradation; GTPase, guanosine triphosphatase.

Given the known clinical differences between CdLS and DEE85 caused by *SMC1A* variants,^35^ we directly compared the transcriptomic profiles of CdLS‐derived LCLs to DEE85 samples to gain insights into the molecular mechanisms underlying these phenotypic divergences. Principal component analysis further underscored these differences. EP nonsense samples formed a distinct cluster separate from CdLS, indicating a clear divergence in gene expression programs. In contrast, EP frameshift and missense samples exhibited greater dispersion, consistent with higher transcriptional heterogeneity (Figure 3A). This analysis identified 587 DEGs (374 were downregulated and 213 upregulated), associated with transcription, translation, DNA replication, and mRNA processing (Figure 3B,C, Table S7). To isolate DEE85‐specific expression patterns, we excluded genes shared with CdLS and controls, yielding 382 uniquely dysregulated genes (Figure 3D). GO analysis of this set revealed enrichment not only in transcriptional regulation but also in chromatin remodeling, angiogenesis, intracellular signaling transduction, and neuronal migration (Figure 3E). Finally, transcriptomic comparison between EP and CdLS cell lines, both carrying missense variants in *SMC1A*, identified 480 DEGs (220 downregulated, 260 upregulated), pointing to altered biological processes including calcium‐mediated signaling, RNA processing, chromatin looping, and DNA repair (Figure 3F,G, Table S8).

These observations support that although both disorders involve *SMC1A* variants, their molecular consequences differ substantially depending on variant type. Nonsense variants appear to exert more severe effects on cohesin function and downstream gene expression.

### 3.3 Comparative molecular dynamics of wild‐type and mutant SMC1A protein structures

**FIGURE 4:**
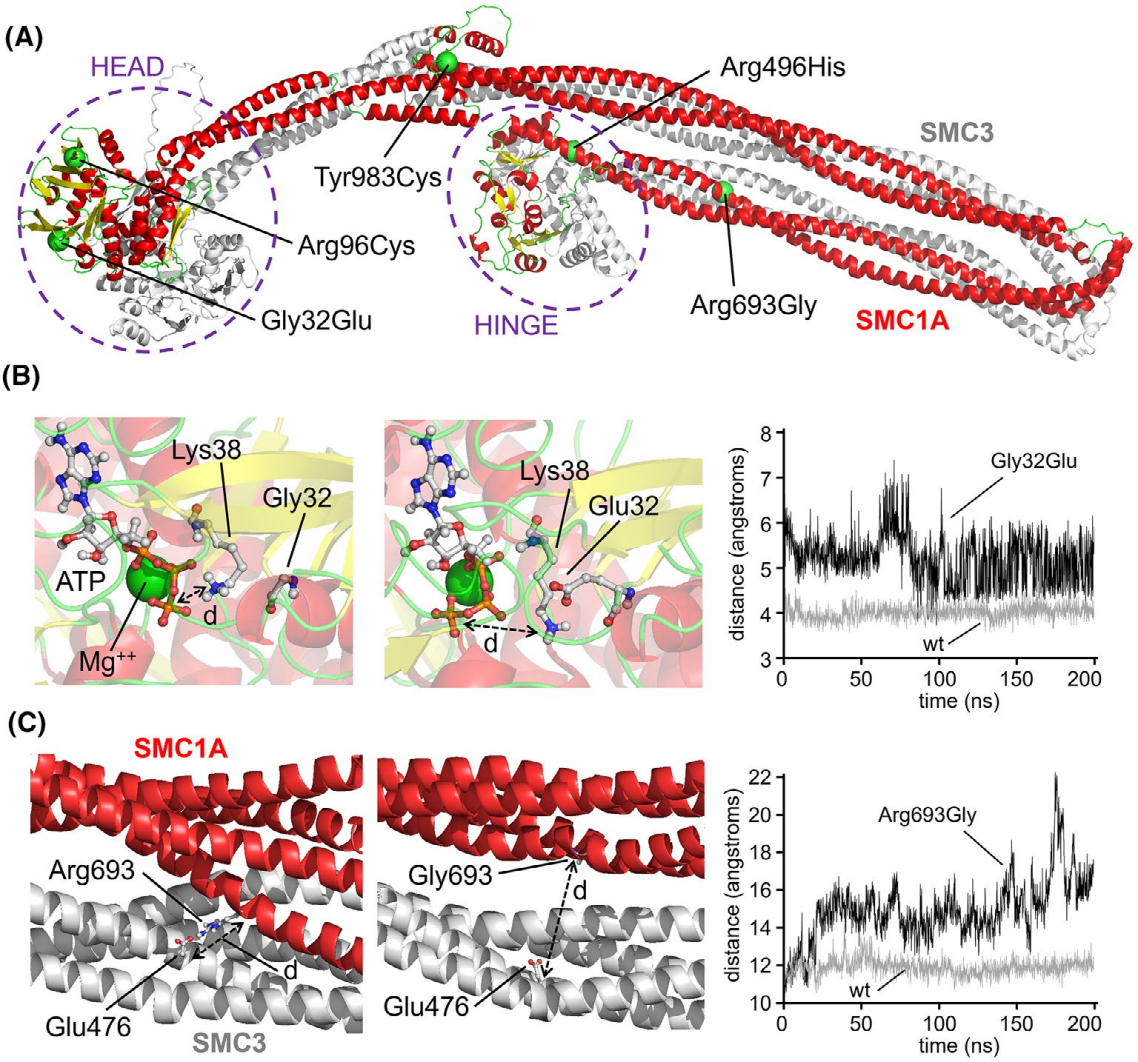
Effect of the Gly32Glu and Arg693Gly variants in the three‐dimensional structure of the SMC1A/SMC3 dimer. (A) Three‐dimensional model of the SMC1A/SMC3 dimer. The SMC1A protein is colored according to its secondary structure elements. The SMC3 protein is shown in gray. The positions of the HEAD and HINGE domains are shown, as well as the positions of the alpha carbons of the amino acids corresponding to the Gly32Glu, Arg96Cys, Arg496His, Arg693Gly, and Tyr983Cys variants of SMC1A (green spheres). (B) Glu32Gly variant. The positions of the amino acids Gly32 (or Glu32) and Lys38, the adenosine triphosphate (ATP) molecule, and the Mg^2+^ atom of the active center of SMC1A are shown after 200 ns of molecular dynamics simulation of the head domain model of the wild‐type (wt) protein (left) or the Gly32Glu variant (center). Right: Plot showing the evolution of the distance “d” (in angstroms) between the nitrogen atom of the amino group of Lys38 and the phosphorus atom of the gamma phosphate group of the ATP molecule during the 200‐ns simulation. Gray line: Wild‐type protein; black line: Gly32Glu variant. (C) Arg693Gly variant. The partial model of the coiled‐coil domain of SMC1A/SMC3 in the vicinity of amino acid Arg693 (or Gly693) of SMC1A is shown after 200 ns of molecular dynamics simulation of the wild‐type protein (left) and the Arg693Gly variant (center). Right: Plot showing the evolution of the distance “d” (in angstroms) between the alpha carbons of Arg693 (or Gly693) of SMC1A and Glu479 of SMC3 during the 200‐ns molecular dynamics simulation. Gray line: Wild‐type protein; black line: Arg693Gly variant. Drawings were generated using PyMOL.

Given that missense variants in the *SMC1A* gene have traditionally been associated with CdLS but have more recently also been identified in cases of DEE85, structural modeling of SMC1A represents a critical tool for elucidating the molecular mechanisms by which specific variants may alter protein conformation and disrupt cohesin function. Therefore, to analyze the potential impact of variants on the SMC1A/SMC3 complex structure, partial models were generated for each domain corresponding to areas surrounding the variants (Figure 4A). Among the five variants studied, only two, Gly32Glu and Arg693Gly, exhibited significant deviations from the wild‐type protein behavior. Figure 4B shows the result of the molecular dynamics simulation of the Gly32Glu variant. In the wild‐type protein, the Gly32 residue is located near the positively charged Lys38 amino acid, which closely contacts the phosphate groups of the ATP molecule in the ATPase active site of SMC1A (Figure 4B, left). Substituting Gly32 with Glu, a negatively charged amino acid, distorts the interaction between Lys38 and ATP (Figure 4B, center). Figure 4B (right) shows the distance between the nitrogen atom of the amino group of Lys38 and the phosphorus atom of the gamma phosphate group of the ATP molecule in the 200‐ns trajectories of the wild‐type protein (gray line) and the Gly32Glu variant (black line). In the wild‐type protein, this distance remains constant at approximately .4 nm, maintaining close contact between the γ phosphate group and the amino group. In contrast, the Gly32Glu variant exhibits a much greater and highly variable distance, indicating local instability that will likely affect the affinity for ATP and/or the ATPase activity of the head domain. Figure 4C shows the possible effect of the Arg693Gly variant on the coiled‐coil domain of the SMC1A/SMC3 dimer. In the wild‐type protein, the positively charged amino acid Arg693 of SMC1A interacts with the negatively charged amino acid Glu476 of SMC3. This interaction stabilizes the position of the coiled coil of both molecules locally (Figure 4C, left). The change from arginine to glycine causes the loss of this interaction, resulting in the separation of the two proteins and local disorganization of the structure (Figure 4C, center). Figure 4C (right) shows the measurement of the distance between the α carbons of the amino acids Arg693 (or Gly693) of SMC1A and Glu479 of SMC3 along the 200‐ns molecular dynamics simulation. The gray line shows that this distance remains constant at approximately 1.2 nm in the wild‐type protein, whereas the black line shows that it increases to values greater than 2.0 nm in the Arg693Gly variant, indicating the local separation of the coiled‐coil domains of both proteins. This local disorganization and possible defect in the dimer's correct structure may explain the variant's effect on the SMC1A/SMC3 ring's functionality. The remaining three variants—Arg96Cys, Arg496His, and Tyr983Cys—did not show substantial structural deviations from the wild‐type protein. Specifically, the Arg96Cys variant is located in a β‐sheet of the head domain, distant from the ATPase centers and surrounded by polar residues (Thr53, Ser103, Tyr105). Substitution with Cys, a similarly polar but smaller residue, preserved the local structure throughout the simulation. The Tyr983Cys variant is located in a flexible loop of the coiled‐coil region, surrounded by polar residues (Gln224, Tyr229, Glu232). The substitution did not induce notable changes in the local environment. Finally, the Arg496His variant maps in an α‐helix of the hinge domain and oriented toward the solvent. Replacement with His, another positively charged residue, did not alter the structural behavior during the simulation.

### 3.4 Ataluren reverses transcriptional and genomic defects in cells with *SMC1A* nonsense variants

Despite advances in understanding the molecular basis of DEE85, no effective therapies currently exist. Ataluren (PTC124), a translational readthrough‐inducing drug, has shown promise in rescuing gene expression in disorders caused by nonsense mutations, including DMD, Shwachman–Diamond syndrome, and cystic fibrosis, both in vitro and in vivo.^26^, ^36^, ^37^, ^38^, ^39^, ^40^ Moreover, it has undergone clinical trials in patients with DMD and cystic fibrosis, supporting its relevance for disorders caused by premature stop codons.^25^, ^41^, ^42^

**FIGURE 5:**
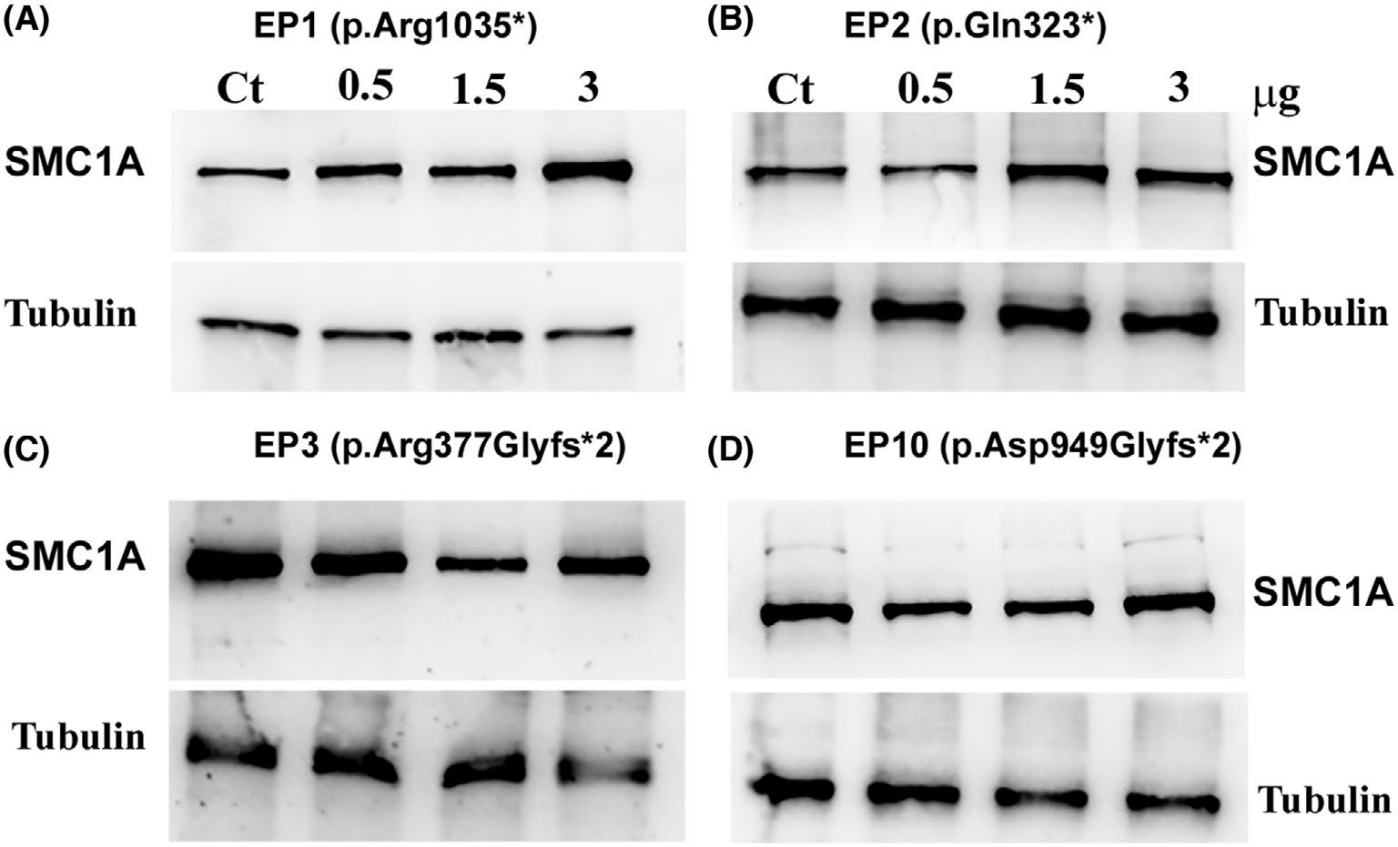
Ataluren restores SMC1A protein expression in lymphoblastoid cell lines (LCLs) carrying nonsense variants but not frameshift mutations. (A) Western blot analysis of SMC1A protein levels in EP1 LCLs (c.3103C>T, p.Arg1035*) following 24‐h treatment with increasing concentrations of ataluren (.5, 1.5, and 3 μg/mL). A dose‐dependent increase in SMC1A expression is observed. (B) Similar analysis in EP2 LCLs (c.901C>T, p.Gln323*) confirms ataluren‐induced restoration of SMC1A protein levels. (C) EP3 LCLs carrying a frameshift variant (c.1063delC) show no detectable change in SMC1A expression upon ataluren treatment, indicating lack of responsiveness. (D) Consistent with this, EP10 LCLs harboring the c.2842_2845dup frameshift variant also fail to respond to ataluren, supporting the specificity of its mechanism of action for nonsense‐mediated translational readthrough. The images shown are representative of two independent experiments.

To evaluate whether ataluren could restore SMC1A protein expression, LCLs from two patients carrying nonsense variants in *SMC1A* (EP1: c.3103C>T, p.Arg1035* and EP2: c.901C>T, p.Gln323*) were treated with increasing doses of ataluren (.5, 1.5, and 3 μg/mL) for 24 h. The dosing rationale was based on clinical pharmacokinetic data. In phase II/III trials in patients with DMD and cystic fibrosis, oral administration of ataluren (4–40 mg/kg) resulted in plasma concentrations typically ranging from 2 to 30 μg/mL.^27^ In addition, in myoblasts from *mdx* mice, Western blotting revealed a dose‐dependent restoration of full‐length dystrophin following ataluren treatment (.6–3 μg/mL).^43^ Accordingly, we selected in vitro concentrations of .5, 1.5, and 3 μg/mL, which fall within or slightly below the clinically observed range, thereby ensuring physiological relevance while minimizing potential cytotoxicity in cell culture. Western blot analysis demonstrated the increase of SMC1A protein in both cell lines (Figure 5A,B, Figure S5). In contrast, EP3 and EP10 cells carrying a frameshift variant, c.1063delC and c.2842_2845dup respectively, showed no response, confirming the specificity of ataluren for nonsense alleles (Figure 5C,D).

**FIGURE 6:**
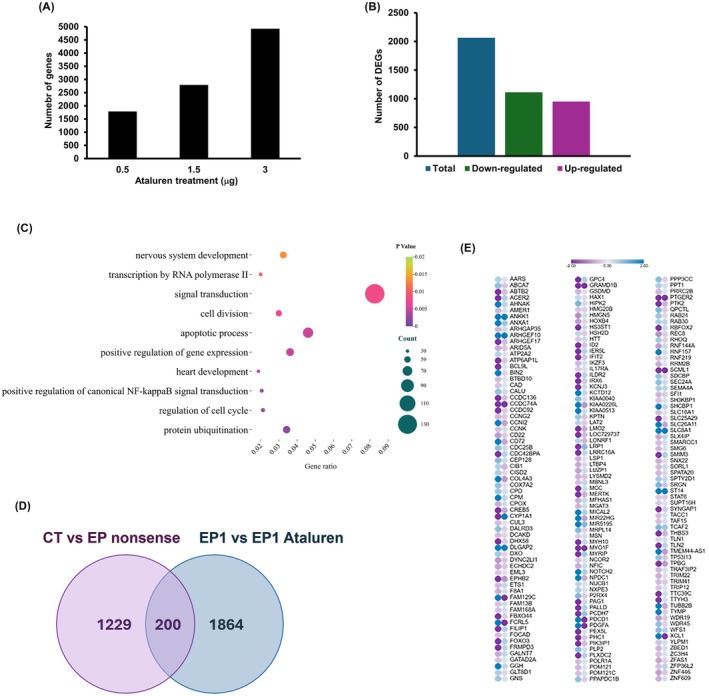
Transcriptomic response to ataluren in EP1 cells carrying a nonsense variant in *SMC1A*. (A) Transcriptomic profiling of EP1 lymphoblastoid cells treated with increasing concentrations of ataluren (.5, 1.5, and 3 μg/mL for 24 h) revealed a dose‐dependent effect, with 1786, 2792, and 4920 differentially expressed genes (DEGs), respectively. (B) Aggregated analysis across all doses identified 2064 DEGs (1113 downregulated and 951 upregulated) when comparing treated versus untreated cells. (C) Gene Ontology enrichment analysis of these DEGs highlighted significant involvement in biological processes such as nervous system development, cell cycle progression, transcriptional regulation, and apoptosis. Dot size reflects the number of DEGs associated with each pathway; color intensity indicates statistical significance (*p*‐value). (D) Venn diagram showing a core set of 200 genes consistently affected across all treatment concentrations. (E) Heatmap illustrating that 116 of these genes (58%) exhibit a reversal in expression direction following treatment, suggesting a robust and specific transcriptional reprogramming induced by ataluren. CT, control cells; EP, epileptic cells.

To determine whether newly synthesized SMC1A is incorporated into the cohesin complex, we performed coimmunoprecipitation of SMC3 with SMC1A. These experiments confirmed the physical association between SMC1A and SMC3, whereas no signal was observed in control Western blotting with IgG‐coated beads (Figure S6). Given the role of *SMC1A* in cohesin‐mediated gene regulation and genome stability, we first investigated the transcriptional impact of ataluren. Transcriptomic profiling of treated EP1 cells revealed a dose‐dependent response, with 1786, 2792, and 4920 DEGs identified at .5, 1.5, and 3 μg/mL, respectively (Figure 6A). When aggregating data across doses and comparing treated versus untreated cells, we detected 2064 DEGs (1113 downregulated and 951 upregulated; Figure 6B, Table S9), enriched in key biological processes including nervous system development, cell cycle and division, transcriptional regulation, and apoptosis (Figure 6C). Notably, 200 genes were consistently affected across all concentrations, with 116 (58%) showing a reversal in expression direction following treatment (Figure 6D,E).

A comparable pattern emerged in EP2 cells, where ataluren modulated the expression of 1152 and 1938 genes at 1.5 and 3 μg/mL, respectively (Figure S7A). Upon pooling the treatment data, we identified 1347 DEGs (703 downregulated, 644 upregulated; Figure S7B and Table S10), again enriched in pathways related to cell proliferation, signal transduction, and cell adhesion (Figure S7C). Consistently, 161 genes were altered across doses, with 44.7% reverting toward control expression levels (Figure S7D,E). Finally, we also assessed spontaneous chromosomal instability. Of 100 metaphases, 24 (EP1) and 21 (EP2) exhibited both gaps and breaks (Figure S8A–C), whereas only 2–3 gaps per 100 metaphases were observed in control cells. These differences were statistically significant (*p* < .05). After ataluren treatment, aberration levels dropped to near control levels (Figure S8A).

In summary, ataluren effectively rescues SMC1A expression in nonsense mutant cells, restores normal gene expression, and reduces genomic instability, supporting its potential as a targeted therapy for DEE85.

## 4 DISCUSSION

This study provides novel insights into the molecular consequences of *SMC1A* variants in patients affected by DEE85, a severe neurodevelopmental diagnosis characterized by early onset, severe ID, and drug‐resistant epilepsy. By performing genome‐wide transcriptomic profiling in LCLs carrying different types of *SMC1A* variants—nonsense, frameshift, and missense—we demonstrate that each variant type is associated with a distinct transcriptional signature. Notably, nonsense variants exerted the most profound effects on global gene expression, with more than 1429 DEGs, compared to 411 and 296 DEGs in frameshift and missense variants, respectively. This aligns with clinical observations that patients with nonsense variants often present with more severe neurodevelopmental phenotypes. In contrast, missense and frameshift variants produced more moderate and heterogeneous effects, suggesting partial retention of cohesin activity. The comparative analysis between variant subgroups revealed minimal overlap in dysregulated genes, with only 15 genes shared across all three mutation types. This limited convergence underscores the complexity of *SMC1A*‐related pathogenesis and suggests that each mutation class may perturb distinct regulatory networks. Interestingly, nonsense and frameshift variants shared a subset of misregulated genes, with most showing concordant expression trends, whereas a few reversed direction, highlighting nuanced differences in downstream effects. The data revealed that dysregulated genes in DEE85 are involved in crucial biological processes including transcriptional regulation, chromatin remodeling, signal transduction, and neuronal migration, pathways directly relevant to brain development and function. These findings reflect the severity of the associated phenotype and suggest that the functional impact of *SMC1A* variants is highly variant type‐dependent and transcriptomic profiling could serve as a valuable tool for stratifying patients and predicting clinical outcomes.

Germline pathogenic variants in the *SMC1A* gene are associated with both CdLS and DEE85. CdLS is generally linked to missense variants or small in‐frame deletions that produce milder clinical manifestations, whereas DEE85 is predominantly caused by loss‐of‐function variants in *SMC1A*.^13^, ^14^, ^35^, ^44^, ^45^ Comparison with CdLS cell lines revealed further transcriptomic divergence, despite their shared genetic origin. CdLS samples exhibited a more restrained transcriptional profile, consistent with the milder clinical phenotype typically associated with missense or in‐frame *SMC1A* variants.^13^, ^14^, ^35^ In contrast, DEE85 samples—particularly those with nonsense variants—displayed extensive dysregulation in genes involved in transcription, translation, DNA replication, and mRNA processing. Principal component analysis confirmed the separation of DEE85 nonsense samples from CdLS, supporting the hypothesis that loss‐of‐function variants in *SMC1A* disrupt cohesin's regulatory functions more severely, leading to broader transcriptomic instability and more aggressive neurological manifestations.

Although computational simulations are not yet sufficient to independently predict pathogenicity, they offer valuable molecular‐scale insights into the impact of missense variants. To elucidate the molecular basis underlying phenotypic differences, we therefore performed structural modeling and molecular dynamics simulations of selected *SMC1A* missense variants. The simulations revealed that only Gly32Glu and Arg693Gly variants significantly affect the structural integrity of the SMC1A/SMC3 complex, potentially impairing cohesin function. Of note, the Gly32Glu variant likely disrupts ATP binding and hydrolysis in the head domain, which could compromise the ATPase‐driven activity essential for cohesin loading and release, and the Arg693Gly variant destabilizes the coiled‐coil interface between SMC1A and SMC3, possibly affecting the mechanical stability and proper assembly of the cohesin ring.

For Arg96Cys, Arg496His, and Tyr983Cys, the absence of observable structural changes does not exclude pathogenic effects. Molecular dynamics simulations do not capture folding‐related perturbations, and these variants may induce misfolding or subtle conformational shifts during protein maturation. In particular, Arg496His, located in the hinge domain and exposed to the solvent, may influence interactions with other cohesin components or DNA. Although structurally stable in simulation, its position suggests potential functional relevance. Supporting this hypothesis, experimental evidence indicates that the Arg496His change impairs DNA repair mechanisms and elevates oxidative stress levels, both of which contribute to genomic instability.^46^, ^47^

Although significant progress has been made in elucidating the molecular mechanisms underlying DEE85, there are still no effective treatments available. Ataluren, a drug that promotes translational readthrough, has demonstrated potential in restoring full‐length protein in diseases caused by nonsense variants—such as DMD—in both in vitro and in vivo studies.^26^ Ataluren treatment of EP1 and EP2 LCLs, both harboring nonsense *SMC1A* variants, resulted in the re‐expression of full‐length SMC1A protein, which was incorporated into the cohesin complex. In contrast, no effect was observed in cells carrying a frameshift variant, confirming the variant‐specific mechanism of action. This observation further supports the notion that ataluren can counteract the molecular defects caused by nonsense variants. Clinical trials have shown that ataluren can slow disease progression in certain DMD patients and improve some aspects of lung function in cystic fibrosis, although its efficacy varies depending on the mutation and individual patient factors.^25^, ^41^, ^42^, ^48^, ^49^ Strikingly, ataluren also reversed the expression of up to 58% of the previously dysregulated genes in these lines and significantly reduced the number of spontaneous chromosomal aberrations, restoring genomic stability to near control levels. These findings strongly suggest that restoration of SMC1A protein can rescue both transcriptional and genomic integrity, highlighting the central role of cohesin dysfunction in DEE85 pathology.

## 5 CONCLUSIONS

Taken together, these results indicate that DEE85 is primarily a transcriptional disorder driven by cohesin dysfunction, and that nonsense *SMC1A* variants have the most deleterious effect on the cellular transcriptome. The success of ataluren in restoring SMC1A protein and reversing molecular defects provides compelling preclinical evidence for its potential as a targeted therapy for a subset of DEE85 patients. In conclusion, this work emphasizes the importance of variant‐type stratification in both mechanistic and therapeutic studies of *SMC1A*‐related disorders. It also identifies ataluren as a promising candidate for personalized intervention in DEE85 patients carrying nonsense variants, opening new avenues for precision medicine in developmental epilepsies.

## AUTHOR CONTRIBUTIONS

**Maddalena Di Nardo**: Investigation; data curation. **Francesca Sardina**: Investigation; data curation. **Maria M. Pallotta:** Investigation; data curation. **Iñigo Marcos‐Alcalde**: Investigation; data curation. **Paulino Gómez‐Puertas**: Conceptualization (supporting); investigation; data curation; writing—review and editing. **Ian D. Krantz**: Conceptualization (supporting); investigation; data curation; writing—review and editing; funding acquisition. **Cinzia Rinaldo**: Conceptualization (supporting); investigation; data curation; writing—review and editing. **Antonio Musio**: Conceptualization (lead); investigation; data curation; formal analysis; writing—original draft preparation; writing—review and editing; funding acquisition.

## FUNDING INFORMATION

This work was supported by the project 202227SYBW_LS3_PRIN2022, by a donation from the Italian SMC1A Association to A.M., and by Spanish Government grant PID2021‐126625OB‐I00 (MCIN/AEI/10.13039/501100011033/FEDER,EU.2022) to P.G.‐P.

## CONFLICT OF INTEREST STATEMENT

None of the authors has any conflict of interest to disclose. We confirm that we have read the Journal's position on issues involved in ethical publication and affirm that this report is consistent with those guidelines.

## Supporting information


Data S1.

## Data Availability

The relevant data supporting the findings of this study are available in this article and its Supporting Information files. All NGS raw files have been deposited into NCBI Sequence Read Archive under accession number PRJNA1370816.
