# Supplementary material for: Mutation type‐specific transcriptomic signatures and readthrough therapy rescue in SMC1A‐related developmental and epileptic encephalopathy
Source: Epilepsia. 2026 Mar 2;67(6):3185–98. doi: 10.1002/epi.70150 (PMC13285250; doi:10.1002/epi.70150)
Supplement: Supplementary file 1 — Data S1. [file EPI-67-3185-s001.pdf]

# 1 SUPPLEMENTARY MATERIAL AND METHODS

## 1.1 Library preparation and RNA sequencing

Samples were processed for RNA-seq analyses as previously described [1]. Briefly, library preparation was performed using the Universal Plus mRNA-Seq kit (Tecan Genomics, Redwood City, CA), following the manufacturer's protocol. RNA samples were quantified and assessed for quality using the Agilent 2100 Bioanalyzer RNA assay (Agilent Technologies, Santa Clara, CA). Final libraries were evaluated using the Qubit 3.0 Fluorometer (Invitrogen, Carlsbad, CA) and the Agilent Bioanalyzer DNA assay. Sequencing libraries were then processed and sequenced in paired-end 150 bp mode on the NovaSeq 6000 platform (Illumina, San Diego, CA).

## 1.2 RNA-Seq analysis

Raw sequencing data were processed using Illumina BCL Convert v3.9.31, which performed base calling, demultiplexing, and adapter masking. During demultiplexing, adapter sequences were masked by converting them to *N* characters, with corresponding base quality scores overwritten to 2 to facilitate downstream trimming using standard quality filtering tools. Subsequent trimming of low-quality bases and residual adapter sequences was carried out using ERNE software [2]. Cleaned reads were then aligned to the *Homo sapiens* reference genome (hg38) using STAR with default parameters. STAR, a splice-aware aligner optimized for RNA-Seq data, enabled accurate mapping and identification of exon-exon junctions [3]. Transcript assembly and quantification were performed with StringTie, allowing reconstruction of full-length transcripts and estimation of expression levels for multiple spliced isoforms at each gene locus [4]. For quality control, the RSeqQC5 package was employed to assess read strand specificity and gene body coverage, ensuring the integrity and reliability of the RNA-Seq dataset [5].

### 1.2.1 Pair-wise differential expression analysis

Differential expression analysis was conducted using DESeq2, which applies a Generalized Linear Model (GLM) to estimate expression levels for each gene and transcript. The method incorporates shrinkage estimation for both dispersion and fold change values, enhancing the stability and

interpretability of the results. This approach allows for a more quantitative assessment, emphasizing the magnitude of differential expression rather than its mere presence. Normalization of count data was performed using the median-of-ratios method, and statistical significance was evaluated through the Wald test [6, 7].

**1.2.2 Pathway analysis and function**

Differentially expressed genes (DEGs)were functionally analysed for associated biological processes using the Database for Annotation, Visualization, and Integrated Discovery (DAVID), version 2025_1 (https://david.ncifcrf.gov). For each annotated term, enrichment was assessed by calculating the corresponding $p$-value, and terms with $p < 0.05$ were considered significantly enriched.

**1.3 Western blotting**

Western blotting was performed as described previously [1]. Briefly, whole-cell protein extracts cells were prepared using a lysis buffer containing 25 µM Tris-HCl (pH 8.0), 55 µM NaCl, 1 µM EDTA, and a protease inhibitor cocktail (Sigma-Aldrich). Protein concentrations were determined using the Bradford Protein Assay (Thermo Scientific). Equal amounts of protein (20 µg per lane) were separated by SDS-PAGE and transferred to nitrocellulose membranes (Amersham). Membranes were incubated with a primary anti-SMC1A antibody (Fortis Life Sciences), followed by a peroxidase-conjugated secondary antibody (Sigma). Detection was performed using a chemiluminescence system (Amersham), and signals were visualized with a Chemidoc imaging system (Bio-Rad). An anti-tubulin antibody (Merck) was used as a loading control. The ImageJ software was used to carry out semiquantitative image analysis of immunoblotting data, expressed by percent of ataluren treatments (0.5, 1.5 and 3 µg/ml)/control ratio.

**1.4 Spontaneous genomic instability assay**

Spontaneous genomic instability in cell lines carrying nonsense *SMC1A* variants was assessed using standard cytogenetic protocols. Briefly, colcemid was added to cell cultures for 90 minutes to arrest cells in metaphase, followed by hypotonic treatment with 0.075 M KCl for 20 minutes at 37°C.

Cells were then fixed with multiple changes of Carnoy's fixative (methanol:acetic acid, 3:1). Fixed cells were dropped onto clean, moist microscope slides. For each patient sample, 100 metaphases were analysed. Chromosomal aberrations, including gaps and breaks, were visualized by Giemsa staining and scored by direct microscopic examination.

**SUPPLEMENTARY REFERENCES**

1. Di Nardo M, Astigiano S, Baldari S, Pallotta MM, Porta G, Pigozzi S, et al. The synergism of SMC1A cohesin gene silencing and bevacizumab against colorectal cancer J Exp Clin Cancer Res. 2024 Feb 16;43:49.
2. Del Fabbro C, Scalabrin S, Morgante M, Giorgi FM. An extensive evaluation of read trimming effects on Illumina NGS data analysis PLoS One. 2013;8:e85024.
3. Dobin A, Davis CA, Schlesinger F, Drenkow J, Zaleski C, Jha S, et al. STAR: ultrafast universal RNA-seq aligner Bioinformatics. 2013 Jan 1;29:15-21.
4. Pertea M, Pertea GM, Antonescu CM, Chang TC, Mendell JT, Salzberg SL. StringTie enables improved reconstruction of a transcriptome from RNA-seq reads Nat Biotechnol. 2015 Mar;33:290-295.
5. Wang L, Wang S, Li W. RSeQC: quality control of RNA-seq experiments Bioinformatics. 2012 Aug 15;28:2184-2185.
6. Love MI, Huber W, Anders S. Moderated estimation of fold change and dispersion for RNA-seq data with DESeq2 Genome Biol. 2014;15:550.
7. Anders S, Huber W. Differential expression analysis for sequence count data Genome Biol. 2010;11:R106.

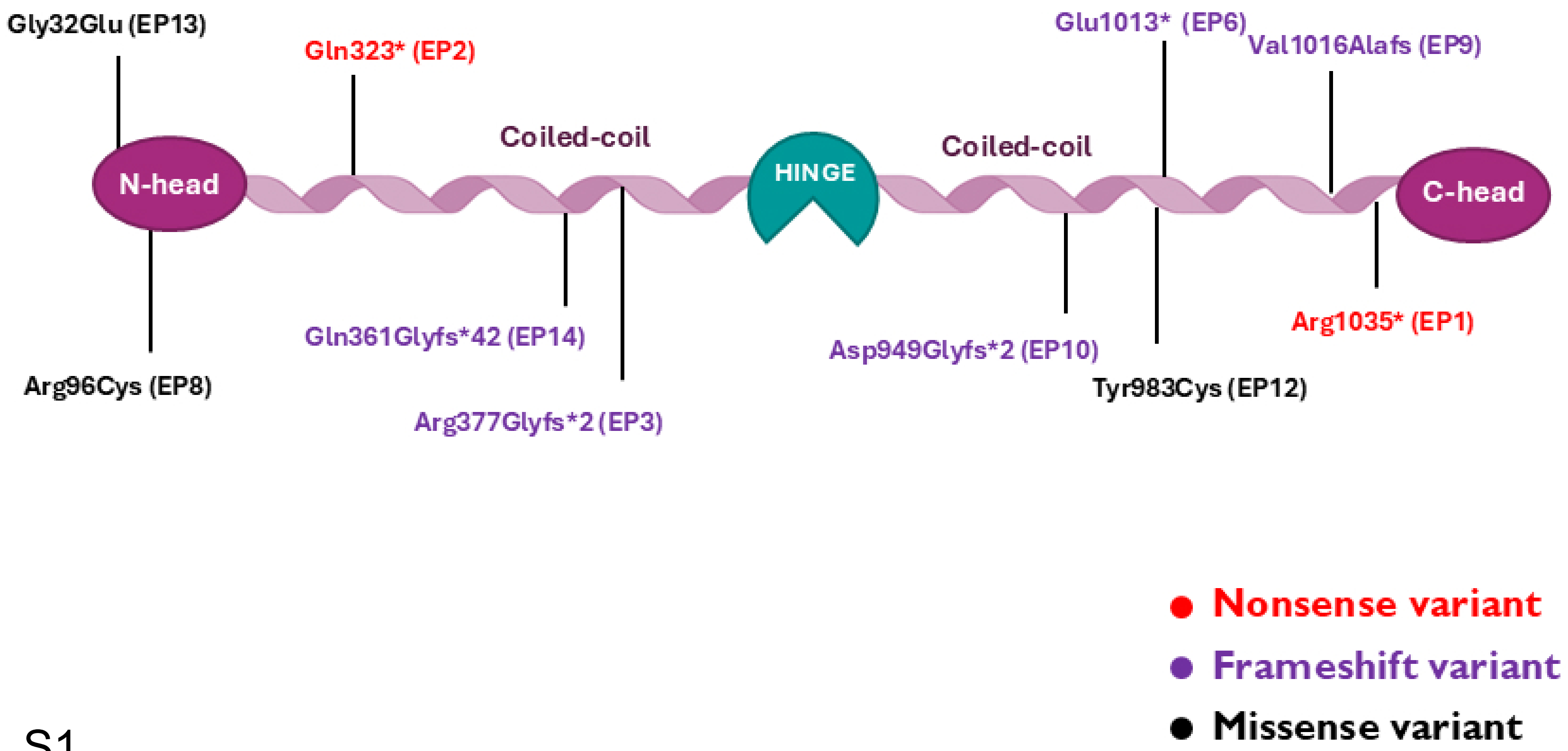
Gly32Glu (EP13)
Gln323* (EP2)
Glu1013* (EP6)
Val1016Alafs (EP9)
Coiled-coil
Coiled-coil
N-head
HINGE
C-head
Arg96Cys (EP8)
Gln361Glyfs*42 (EP14)
Arg377Glyfs*2 (EP3)
Asp949Glyfs*2 (EP10)
Tyr983Cys (EP12)
Arg1035* (EP1)
Nonsense variant
Frameshift variant
Missense variant

Fig. S1

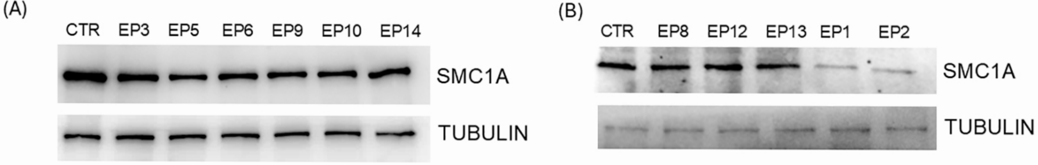
(A)
CTR
EP3
EP5
EP6
EP9
EP10
EP14
SMC1A
TUBULIN
(B)
CTR
EP8
EP12
EP13
EP1
EP2
SMC1A
TUBULIN

Fig. S2

Fig. S3

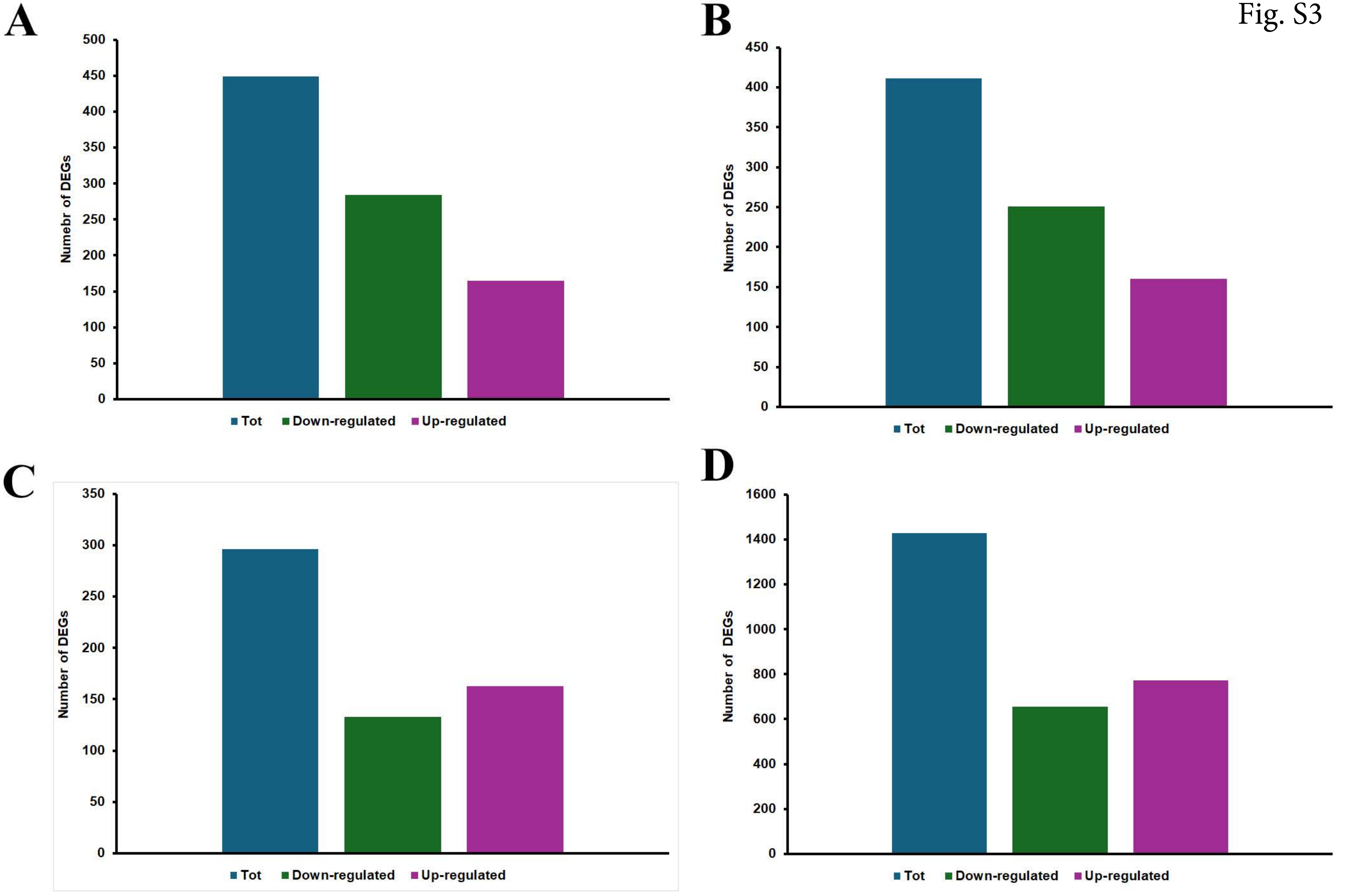
A
Numebr of DEGs
500
450
400
350
300
250
200
150
100
50
0
Tot
Down-regulated
Up-regulated
B
Number of DEGs
450
400
350
300
250
200
150
100
50
0
Tot
Down-regulated
Up-regulated
C
Number of DEGs
350
300
250
200
150
100
50
0
Tot
Down-regulated
Up-regulated
D
Number of DEGs
1600
1400
1200
1000
800
600
400
200
0
Tot
Down-regulated
Up-regulated

Fig. S4

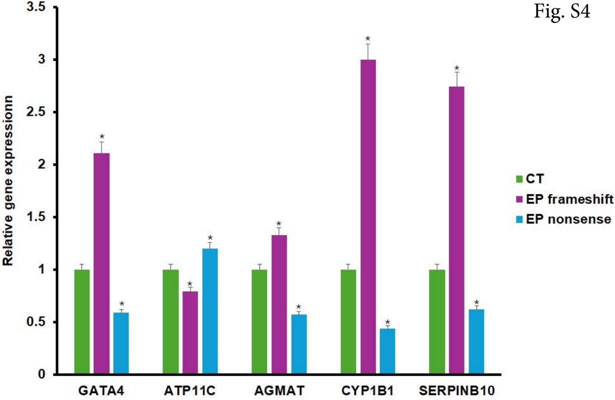
3.5
3
2.5
2
1.5
1
0.5
0
Relative gene expressionn
CT
EP frameshift
EP nonsense
GATA4
ATP11C
AGMAT
CYP1B1
SERPINB10

Fig. S5

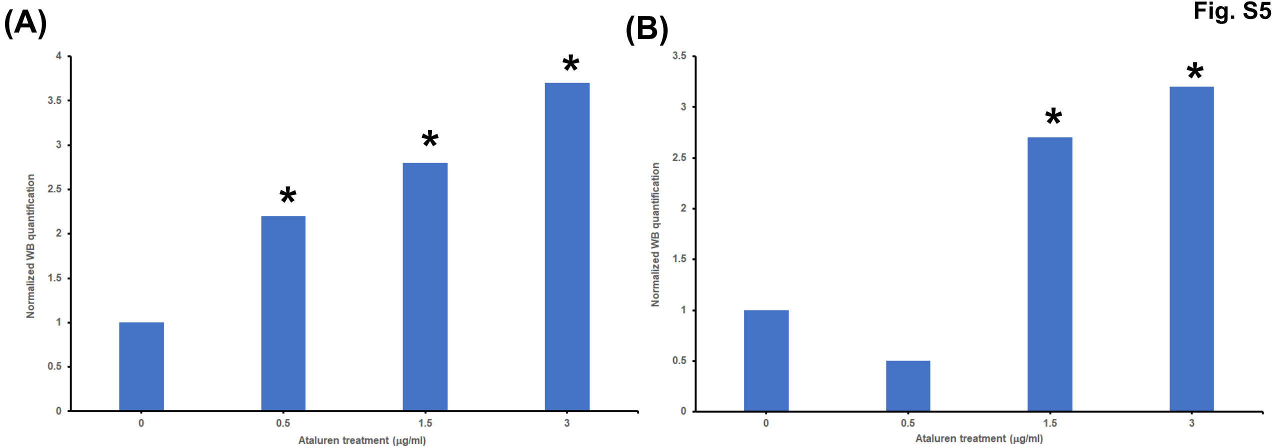
(A)
Normalized WB quantification
0
0.5
1
1.5
2
2.5
3
3.5
4
0
0.5
1.5
3
Ataluren treatment (µg/ml)
(B)
Normalized WB quantification
0
0.5
1
1.5
2
2.5
3
3.5
0
0.5
1.5
3
Ataluren treatment (µg/ml)

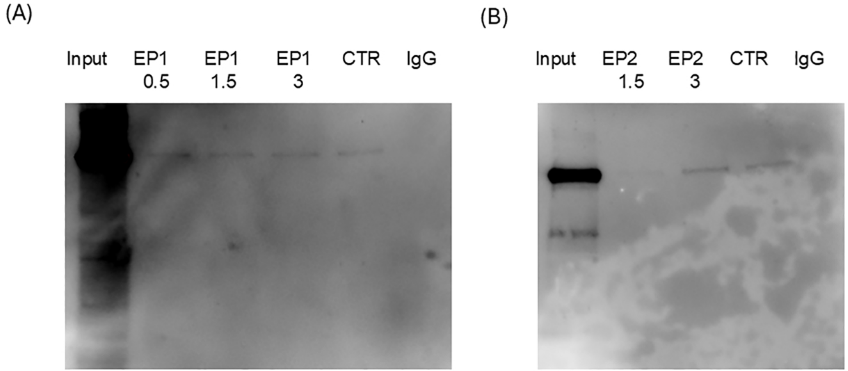


**Fig. S6**

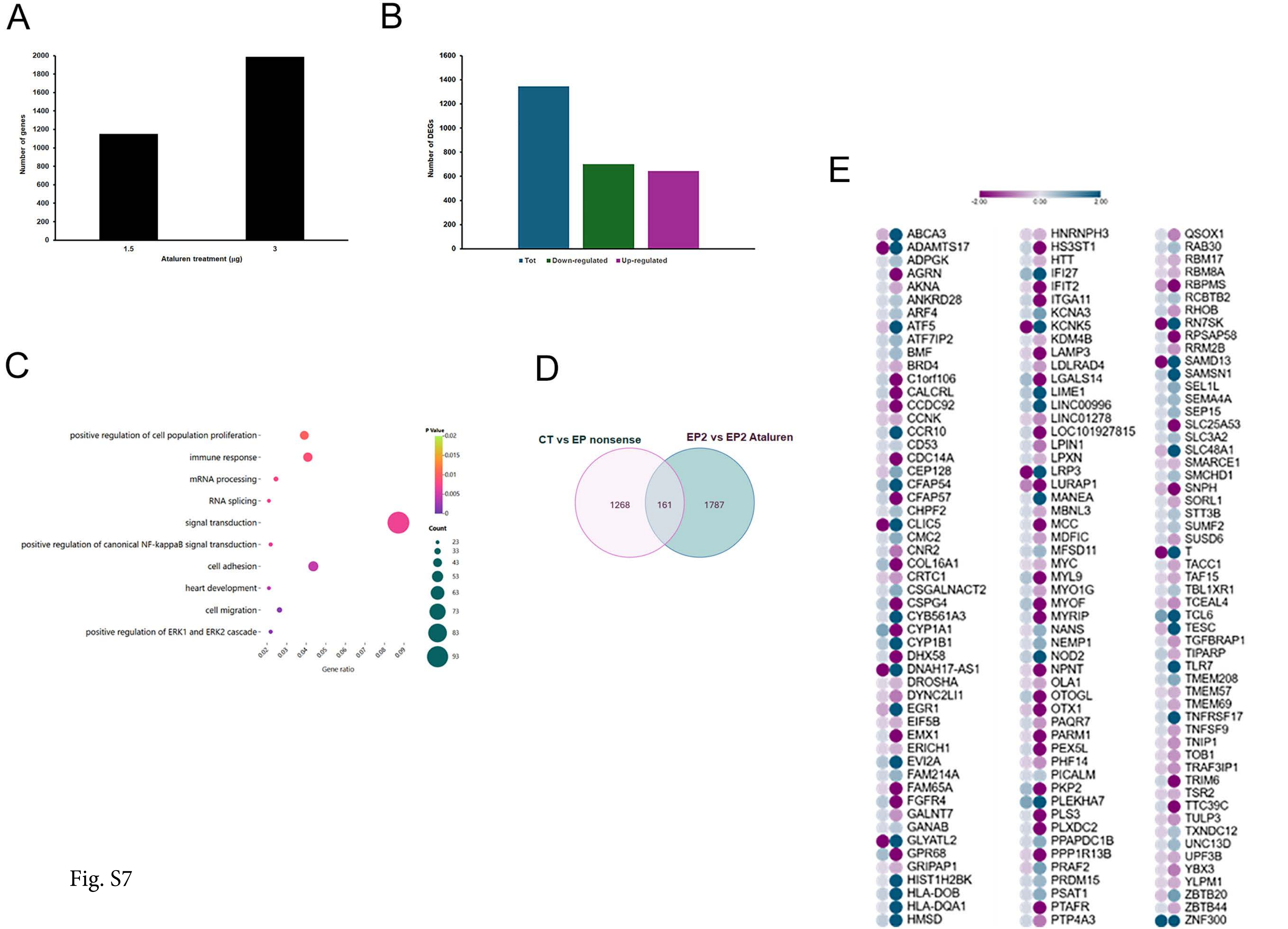
A
Number of genes
2000
1800
1600
1400
1200
1000
800
600
400
200
0
1.5
3
Ataluren treatment (µg)
B
Number of DEGs
1600
1400
1200
1000
800
600
400
200
0
Tot
Down-regulated
Up-regulated
C
positive regulation of cell population proliferation
immune response
mRNA processing
RNA splicing
signal transduction
positive regulation of canonical NF-kappaB signal transduction
cell adhesion
heart development
cell migration
positive regulation of ERK1 and ERK2 cascade
0.02
0.03
0.04
0.05
0.06
0.07
0.08
0.09
Gene ratio
P Value
0.02
0.015
0.01
0.005
0
Count
23
33
43
53
63
73
83
93
D
CT vs EP nonsense
EP2 vs EP2 Ataluren
1268
161
1787
E
-2.00
0.00
2.00
ABCA3
ADAMTS17
ADPGK
AGRN
AKNA
ANKRD28
ARF4
ATF5
ATF7IP2
BMF
BRD4
C1orf106
CALCRL
CCDC92
CCNK
CCR10
CD53
CDC14A
CEP128
CFAP54
CFAP57
CHPF2
CLIC5
CMC2
CNR2
COL16A1
CRTC1
CSGALNACT2
CSPG4
CYB561A3
CYP1A1
CYP1B1
DHX58
DNAH17-AS1
DROSHA
DYNC2LI1
EGR1
EIF5B
EMX1
ERICH1
EVI2A
FAM214A
FAM65A
FGFR4
GALNT7
GANAB
GLYATL2
GPR68
GRIPAP1
HIST1H2BK
HLA-DOB
HLA-DQA1
HMSD
HNRNPH3
HS3ST1
HTT
IFI27
IFIT2
ITGA11
KCNA3
KCNK5
KDM4B
LAMP3
LDLRAD4
LGALS14
LIME1
LINC00996
LINC01278
LOC101927815
LPIN1
LPXN
LRP3
LURAP1
MANEA
MBNL3
MCC
MDFIC
MFSD11
MYC
MYL9
MYO1G
MYOF
MYRIP
NANS
NEMP1
NOD2
NPNT
OLA1
OTOGL
OTX1
PAQR7
PARM1
PEX5L
PHF14
PICALM
PKP2
PLEKHA7
PLS3
PLXDC2
PPAPDC1B
PPP1R13B
PRAF2
PRDM15
PSAT1
PTAFR
PTP4A3
QSOX1
RAB30
RBM17
RBM8A
RBPMS
RCBTB2
RHOB
RN7SK
RPSAP58
RRM2B
SAMD13
SAMSN1
SEL1L
SEMA4A
SEP15
SLC25A53
SLC3A2
SLC48A1
SMARCE1
SMCHD1
SNPH
SORL1
STT3B
SUMF2
SUSD6
T
TACC1
TAF15
TBL1XR1
TCEAL4
TCL6
TESC
TGFBRAP1
TIPARP
TLR7
TMEM208
TMEM57
TMEM69
TNFRSF17
TNFSF9
TNIP1
TOB1
TRAF3IP1
TRIM6
TSR2
TTC39C
TULP3
TXNDC12
UNC13D
UPF3B
YBX3
YLPM1
ZBTB20
ZBTB44
ZNF300
Fig. S7

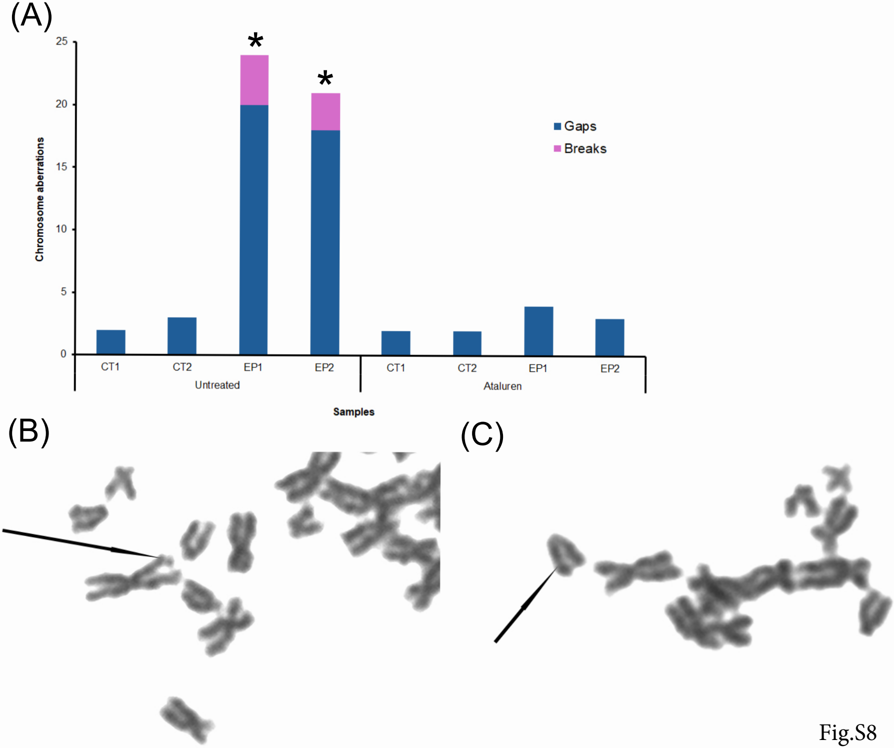

(A)
25
20
15
10
5
0
Chromosome aberrations
*
*
Gaps
Breaks
CT1
CT2
EP1
EP2
Untreated
CT1
CT2
EP1
EP2
Ataluren
Samples
(B)
(C)


Fig.S8

**SUPPLEMENTARY FIGURE LEGENDS**

**Supplementary Figure 1.** Localization of amino acid changes identified in DEE85 patients carrying variants in the *SMC1A* gene. The protein length is not in scale.

**Supplementary Figure 2.** (A) Protein blotting revealed that SMC1A levels in EP cells with frameshift variants (EP3, EP5, EP6, EP9, EP10, and EP14) were comparable to controls. (B) Cells carrying missense variants (EP8, EP12, EP13) showed the same pattern, while EP1 and EP2, harbouring nonsense variants, displayed decreased SMC1A protein levels.

**Supplementary Figure 3.** (A) Differential gene expression analysis (DGEA) comparing EP cell lines to controls identified 449 DEGs (284 were upregulated and 165 downregulated). (B) EP cell lines with frameshift variants showed 411 DEGs (251 upregulated and 160 downregulated). (C) Missense variants affected 296 genes (133 upregulated and 163 downregulated). (D) Nonsense variants caused the most extensive changes, with 1,429 misregulated genes (656 upregulated and 773 downregulated).

**Supplementary Figure 4.** RNA-seq data validation through quantitative-PCR.

**Supplementary Figure 5**. ImageJ software was employed for semiquantitative analysis of immunoblotting data. (A) Ataluren restored SMC1A protein expression in EP1 LCLs carrying the c.901C>T, p.Arg1035 nonsense variant. (B) A similar analysis in EP2 LCLs, harbouring the c.901C>T, p.Gln323* nonsense variant, confirmed ataluren-induced restoration of SMC1A protein levels, with the exception of treatment at 0.5 μg/ml.

**Supplementary Figure 6**. (A) Newly synthesized SMC1A coimmunoprecipitated with SMC3 in EP1 and (B) EP2 cell lines carrying nonsense variants. No signal was detected in the IPs using IgG-coated beads.

**Supplementary Figure 7.** Transcriptomic response to ataluren in EP2 cells carrying a nonsense variant in *SMC1A*. (A) Transcriptomic profiling of EP2 lymphoblastoid cells treated with increasing concentrations of ataluren (1.5, and 3 μg/ml for 24 hours) revealed a dose-dependent effect, with 1,152 and 1,938 DEGs, respectively. (B) Upon pooling the treatment data, we identified 1,347

DEGs (703 downregulated, 644 upregulated). (C) GO enrichment analysis of these DEGs highlighted significant involvement in biological processes such again enriched in pathways related to cell proliferation, signal transduction, cell adhesion. (D) Consistently, 161 genes were altered across doses, with 44.7% reverting toward control expression levels.

**Supplementary Figure 8.** (A) Karyotypic analysis of 100 Giemsa-stained metaphase spreads showed that EP1 and EP2 cells displayed markedly elevated chromosomal aberration frequencies (24 and 21 per 100 metaphases, respectively) compared with controls (2–3/100). Following ataluren treatment, aberration levels decreased to values comparable to controls. Representative partial metaphases illustrating chromosomal abnormalities are shown in (B) a break and (C) a gap in EP1 cells. Aberrations are indicated by arrows.

Supplementary Table 1. EP, CdLS and control cell lines used for the transcriptome analysis.

| Sample | SMC1A variant | Amino acid change | Type |
|---|---|---|---|
| EP1 | c.3103C>T | p.Arg1035* | Nonsense |
| EP2 | c.901C>T | p.Gln323* | Nonsense |
| EP3 | c.1063delC | p.Arg377Glyfs*2 | Frameshift |
| EP5 | 19.5 Kb deletion | | Frameshift |
| EP6 | c.1609delG | p.Glu1013* | Frameshift |
| EP8 | c.286C>T | p.Arg96Cys | Missense |
| EP9 | c.3046_3048delGTGindG | p.Val1016Alafs | Frameshift |
| EP10 | c.2842_2845dup | p.Asp949Glyfs*2 | Frameshift |
| EP12 | c.2948A>G | Tyr983Cys | Missense |
| EP13 | c.95C>A | Gly32Glu | Missense |
| EP14 | c.1078_1079delAG | Gln361Glyfs*42 | Frameshift |
| CdL363 | c.2077C>G | Arg693Gly | Missense |
| CdL565 | c.2046_2048delAGA | Glu683del | Deletion |
| CdL060 | c.1487G>A | Arg496His | Missense |
| LCL1 | | | Control |
| LCL3 | | | Control |
| LCL4 | | | Control |

Supplementary Table 2. Primer used for RNA-seq data validation.

| GENE | PRIMER FORWARD | PRIMER REVERSE |
|---|---|---|
| **GATA4** | CAGCAAGTGAGAAGCGAGAC | GATGGCACTGGCTGAACTT |
| **ATP11C** | TGGAAGAACTGCGTGTCCT | GCAGCAGCATCATCATCTTC |
| **AGMAT** | TCACCTTCGTGGTCATCGAC | GCAGCAGAAGGTGCAGAAAC |
| **CYP1B1** | CCTGGAGACCTTCGGCAAC | GGTGATGAAGGCGTTGTTGG |
| **SERPINB10** | GGACCTCAGCCTGACTACCA | CAGGATGAAGTCCAGGGCAT |
| **ABHD6** | TGCTGGTCATCTTCGGCTAC | GGATGACGATGACGAGGAAG |
| **HPRT** | AGCCAGACTTTGTTGGATTTG | TACTAAGCAGATGGCCACAGA |

Supplementary Table 3. Differentially expressed genes (up- and downregulated) in EP cell lines compared to controls.

Down-regulated

| Gene | log2FoldChange |
|---|---|
| PNMAL1 | -8,20827E+14 |
| GSTM1 | -7,66371E+14 |
| RPRM | -5,93135E+14 |
| FRAS1 | -5,68833E+14 |
| SEMA3A | -5,41414E+14 |
| ASB9 | -5,25452E+14 |
| PITX2 | -5,185E+14 |
| TRIM31 | -5,10712E+14 |
| MIR646HG | -4,96669E+14 |
| LOC101926935 | -4,94256E+14 |
| UGT2A3 | -4,8869E+14 |
| LINC01087 | -4,82924E+14 |
| WSCD1 | -4,77784E+14 |
| BMP3 | -4,77487E+14 |
| RBPMS | -4,76671E+14 |
| RNF217 | -4,72396E+14 |
| PLS3 | -4,71219E+14 |
| FOXG1 | -4,68042E+14 |
| ITGA2 | -4,36085E+14 |
| FAM71D | -4,35572E+14 |
| CLDN11 | -4,2429E+14 |
| LINC01426 | -4,19238E+14 |
| OR4C6 | -4,18478E+14 |
| TCF7L1 | -4,12116E+14 |
| CCDC149 | -4,02013E+14 |
| NOL4 | -3,98752E+14 |
| HOGA1 | -3,95391E+14 |
| MMP23A | -3,83062E+14 |
| FAM86B1 | -3,72323E+14 |
| DNAH14 | -3,68768E+14 |
| KCNJ2 | -3,68443E+14 |
| ANXA3 | -3,6745E+14 |
| FLJ46906 | -3,5871E+14 |
| RP1L1 | -3,56662E+14 |
| HIC1 | -3,53786E+14 |
| SOWAHC | -3,52624E+14 |
| LINC01320 | -3,49907E+14 |
| USP32P1 | -3,49847E+14 |
| SEMA5A | -3,47181E+14 |
| ST7-OT4 | -3,44031E+14 |
| C14orf132 | -3,4084E+14 |
| NR2F2 | -3,37902E+14 |

| | |
|---|---|
| IL1B | -3,3724E+14 |
| PRKCH | -3,20962E+14 |
| PTPRG | -3,18772E+14 |
| LOC102724301 | -3,16755E+14 |
| SPEF2 | -3,09783E+14 |
| GPM6A | -3,07364E+14 |
| CASC10 | -3,07233E+14 |
| TNIP3 | -3,058E+14 |
| CREB3L3 | -3,04867E+14 |
| PCDHGB4 | -3,03269E+14 |
| LOC100130298 | -3,0144E+14 |
| TEKT4P2 | -2,99621E+14 |
| AMOTL1 | -2,97156E+14 |
| LINC01252 | -2,91069E+14 |
| BANK1 | -2,88131E+14 |
| CYP1A1 | -2,78283E+14 |
| MYRF | -2,71008E+14 |
| ROBO3 | -2,65134E+14 |
| NAP1L2 | -2,61833E+14 |
| TOX | -2,58625E+14 |
| RGS6 | -2,55021E+14 |
| ADGRA3 | -2,54345E+14 |
| PMCH | -2,53014E+14 |
| MAN1C1 | -2,49914E+14 |
| LGR4 | -2,45858E+14 |
| OGDHL | -2,44852E+14 |
| FAM198B | -2,44225E+14 |
| GABRB2 | -2,41138E+14 |
| PLXNA2 | -2,37974E+14 |
| DPP4 | -2,35894E+14 |
| FCRL4 | -2,34298E+14 |
| ITGA5 | -2,33453E+14 |
| FCGRT | -2,29157E+14 |
| COL16A1 | -2,2841E+14 |
| ANKRD18A | -2,27494E+14 |
| ROBO1 | -2,2461E+14 |
| F5 | -2,21425E+14 |
| ACKR3 | -2,20762E+14 |
| HLA-DRB6 | -2,19721E+14 |
| NPIPA5 | -2,17215E+14 |
| CCL17 | -2,17102E+14 |
| PARD3 | -2,11667E+14 |
| MYH10 | -2,11612E+14 |
| SIGLEC14 | -2,09967E+14 |
| LOC729737 | -2,05041E+14 |
| HS3ST1 | -2,00454E+14 |
| STAM-AS1 | -1,98999E+14 |

| | |
|---|---|
| RAB37 | -1,98566E+14 |
| ARHGEF17 | -1,94247E+14 |
| CSRNP3 | -1,91191E+14 |
| ARRB1 | -1,9084E+14 |
| UST | -1,90104E+14 |
| EDARADD | -1,83084E+14 |
| HLA-G | -1,79918E+14 |
| DISP2 | -1,77461E+14 |
| TNFRSF19 | -1,76789E+14 |
| CR1 | -1,76746E+14 |
| RILPL1 | -1,73847E+14 |
| CFP | -1,71432E+14 |
| UBQLNL | -1,69688E+14 |
| PCGF2 | -1,64906E+14 |
| TNFSF12 | -1,62912E+14 |
| NCALD | -1,61265E+14 |
| GIMAP6 | -1,60845E+14 |
| LOC554206 | -1,58673E+14 |
| ARHGAP24 | -1,57518E+14 |
| CASKIN2 | -1,56479E+14 |
| DLG4 | -1,54236E+14 |
| TMEM229B | -1,53871E+14 |
| ZBTB47 | -1,52845E+14 |
| C1orf106 | -1,50916E+14 |
| AMZ1 | -1,5039E+14 |
| NHSL1 | -1,47954E+14 |
| ANKRD34A | -1,45973E+14 |
| PTK2 | -1,41729E+14 |
| MS4A14 | -1,40071E+14 |
| MGAT4A | -1,38696E+14 |
| MPEG1 | -1,35357E+14 |
| ID2 | -1,35285E+14 |
| ZNF362 | -1,31528E+14 |
| NUDT13 | -1,31345E+14 |
| SMIM3 | -1,30358E+14 |
| GPR68 | -1,29935E+14 |
| CCDC122 | -1,27383E+14 |
| EMID1 | -1,26106E+14 |
| KCND2 | -1,2598E+14 |
| LRP6 | -1,23211E+14 |
| TRO | -1,22209E+14 |
| MBOAT2 | -1,21753E+14 |
| ACY1 | -1,21709E+14 |
| CLIC6 | -1,17583E+14 |
| CDK5R1 | -1,17067E+14 |
| MNS1 | -1,1651E+14 |
| TMEM132A | -1,15355E+14 |

| | |
|---|---|
| KIF26B | -1,11587E+14 |
| RIMKLB | -1,07126E+14 |
| NFATC2 | -1,05016E+14 |
| SLC45A3 | -1,04539E+14 |
| IL2RB | -1,02074E+14 |
| EOMES | -5,27506E+13 |
| KCNJ12 | -5,08884E+13 |
| FCRL3 | -2,76882E+13 |
| SYNGR1 | -2,59966E+13 |
| SORBS2 | -2,16873E+13 |
| BHLHE22 | -1,76857E+13 |
| TUBA8 | -1,59371E+13 |
| WWTR1 | -1,55832E+13 |
| LIN7A | -1,51832E+13 |
| LRRIQ3 | -1,47034E+13 |
| SEPN1 | -1,43195E+13 |
| H1F0 | -1,38507E+13 |
| NEURL2 | -1,16383E+13 |
| METRN | -1,14342E+13 |
| PHC1 | -1,07823E+13 |
| NRARP | -1,02328E+13 |
| DNASE1L3 | -1,48911E+12 |
| C1QTNF9B-AS1 | -0,985454224 |
| THBS3 | -0,977874366 |
| SNHG25 | -0,960205606 |
| ZGLP1 | -0,939135092 |
| TTC39C | -0,926096682 |
| IL16 | -0,921235249 |
| FAM86B3P | -0,919168895 |
| PIK3R6 | -0,909975263 |
| TMEM136 | -0,905680193 |
| LMO7 | -0,901516508 |
| LRP5 | -0,894688134 |
| MCPH1-AS1 | -0,891687392 |
| RNF144A | -0,88431173 |
| P2RY11 | -0,85299404 |
| NT5E | -0,846372078 |
| CBLB | -0,833118839 |
| ETS2 | -0,828824273 |
| KTN1-AS1 | -0,789375954 |
| CDPF1 | -0,785252515 |
| PHLDB3 | -0,778528068 |
| RRN3P1 | -0,778491349 |
| GSAP | -0,775964618 |
| CTSC | -0,769565071 |
| HMGN5 | -0,76221232 |
| FAM86DP | -0,742537069 |

| | |
|---|---|
| LZTS2 | -0,741160553 |
| AHR | -0,731280741 |
| DHRS4L2 | -0,72720644 |
| GRAMD3 | -0,725952668 |
| SPINT1 | -0,719161926 |
| CYTH3 | -0,718206286 |
| DNAJB5 | -0,713712846 |
| EID2B | -0,697360344 |
| ENG | -0,672921847 |
| PAAF1 | -0,659604108 |
| ENO3 | -0,658241902 |
| TSEN54 | -0,65186642 |
| TESPA1 | -0,646300072 |
| TMEM198B | -0,645561989 |
| CBLN3 | -0,635158782 |
| SNX29 | -0,629907255 |
| RBM26-AS1 | -0,615843983 |
| TSPYL2 | -0,610224537 |
| TCEAL4 | -0,608368853 |
| TCEAL1 | -0,605725123 |
| IKZF2 | -0,600613951 |
| ZNF579 | -0,594709596 |
| CCDC28B | -0,594633516 |
| PSMD5-AS1 | -0,590040786 |
| CHST14 | -0,58996655 |
| FBF1 | -0,588083112 |
| TFPT | -0,544113221 |
| RAI1 | -0,534427906 |
| PCED1A | -0,52647624 |
| SGSM2 | -0,524915201 |
| SIK2 | -0,520957833 |
| INAFM2 | -0,510703922 |
| AGAP3 | -0,495296481 |
| HEATR6 | -0,495207119 |
| DHFRL1 | -0,492836059 |
| PABPN1 | -0,489830648 |
| IFT81 | -0,479398202 |
| TPCN1 | -0,47478551 |
| MAPK12 | -0,466215099 |
| ZNF512B | -0,466064391 |
| FAM78A | -0,452300942 |
| SVIP | -0,447001319 |
| ACP6 | -0,445471604 |
| SMG6 | -0,43959945 |
| ZNF496 | -0,43651371 |
| CHKA | -0,434868706 |
| DAPP1 | -0,434618158 |

| | |
|---|---|
| CDC42EP3 | -0,432809206 |
| BRD3 | -0,4302674 |
| CEPT1 | -0,427983259 |
| LRRC20 | -0,427673435 |
| ZNF74 | -0,427340412 |
| FBXL19 | -0,426918644 |
| SSBP4 | -0,42017247 |
| COPRS | -0,418211158 |
| ALKBH4 | -0,417943487 |
| MSANTD2 | -0,410055636 |
| TMEM110 | -0,405645454 |
| ZSWIM4 | -0,404179178 |
| CCDC125 | -0,389049574 |
| ZMYM3 | -0,38605345 |
| HDGFRP2 | -0,384484635 |
| MBOAT7 | -0,383439707 |
| ZNF234 | -0,376489949 |
| MAPK7 | -0,376013396 |
| RPIA | -0,374716402 |
| SCFD2 | -0,357612432 |
| TBC1D22B | -0,355402311 |
| DCAKD | -0,355095059 |
| CALM3 | -0,355023603 |
| EML3 | -0,344819967 |
| SUSD6 | -0,339704113 |
| BIVM | -0,333750078 |
| ERCC2 | -0,331373126 |
| CSK | -0,324539999 |
| TAF15 | -0,319962724 |
| GATAD2A | -0,298804748 |
| STX4 | -0,295477994 |
| TRIP6 | -0,287009852 |
| HNRNPA2B1 | -0,285270834 |
| CCDC71 | -0,283311392 |
| RPS6KA4 | -0,280735375 |
| ANP32A | -0,279886617 |
| C11orf73 | -0,278587248 |
| CARM1 | -0,27638242 |
| UCK2 | -0,270240571 |
| CDC34 | -0,262468567 |
| PBX2 | -0,259764428 |
| UCKL1 | -0,258735147 |
| INTS9 | -0,254838514 |
| HNRNPH3 | -0,252344322 |
| CTNNBL1 | -0,250302252 |
| AKT1 | -0,235450318 |
| TRIM11 | -0,22910094 |

| | |
|---|---|
| PDCD7 | -0,206054491 |
| NACC1 | -0,205113516 |
| CACTIN | -0,198329218 |
| FAM192A | -0,182825964 |
| NAA60 | -0,179087885 |
| FIP1L1 | -0,174927809 |
| ATXN2L | -0,167708907 |

Up-regulated

| Gene | log2FoldChange |
|---|---|
| CAPZA1 | 0,167452553 |
| RAB5A | 0,192710041 |
| SEC22A | 0,227184502 |
| BRF2 | 0,238598264 |
| ITFG2 | 0,248152999 |
| ERO1A | 0,257068851 |
| TM9SF3 | 0,258506768 |
| RABGEF1 | 0,258980243 |
| SELT | 0,268865392 |
| SCFD1 | 0,270459721 |
| CHMP2B | 0,280320332 |
| TMED4 | 0,288449622 |
| RANBP9 | 0,290505753 |
| GATC | 0,294139612 |
| ZNF562 | 0,309298348 |
| ATG4A | 0,311540617 |
| CASC4 | 0,316829517 |
| SDE2 | 0,326418219 |
| ATF2 | 0,328938753 |
| MAGT1 | 0,329052132 |
| PPT1 | 0,329157896 |
| TTC37 | 0,329999584 |
| PNPLA8 | 0,335605042 |
| TM2D3 | 0,3414044 |
| RETSAT | 0,341611108 |
| EMC3 | 0,342661237 |
| GZF1 | 0,344743941 |
| ZNF136 | 0,358248327 |
| CINP | 0,360494998 |
| ADPGK | 0,361570073 |
| GSKIP | 0,361748462 |
| USP48 | 0,364003615 |
| NGLY1 | 0,364877872 |
| ITFG1 | 0,367310608 |
| SLC35A5 | 0,375642186 |
| TVP23B | 0,380929974 |

| | |
|---|---|
| CD46 | 0,383746215 |
| RAP1B | 0,387287851 |
| DTWD1 | 0,388045326 |
| GBA | 0,392247219 |
| TAPT1 | 0,405607504 |
| CDKN1B | 0,426240648 |
| ENTPD4 | 0,443655426 |
| SEC24D | 0,445483947 |
| BTN3A1 | 0,448997726 |
| SARAF | 0,464832742 |
| GNE | 0,466781706 |
| CHPF2 | 0,467195274 |
| LAMP2 | 0,46961364 |
| MAP4K3 | 0,47055624 |
| ZNF709 | 0,473793226 |
| RHBDD1 | 0,476462149 |
| P2RY10 | 0,478648502 |
| JOSD2 | 0,489875628 |
| ZNF527 | 0,49313342 |
| MYO5A | 0,497522595 |
| LMAN1 | 0,499844727 |
| LONRF1 | 0,507851885 |
| POLE4 | 0,511491277 |
| ASPHD2 | 0,521598702 |
| ZXDB | 0,522668142 |
| CLIP4 | 0,53427951 |
| KCTD7 | 0,54401132 |
| C16orf54 | 0,551610009 |
| GNS | 0,556083411 |
| RELL1 | 0,56134346 |
| PPP3CC | 0,563315472 |
| C10orf32 | 0,565162272 |
| C15orf57 | 0,565246639 |
| JAK2 | 0,580831218 |
| OSTM1 | 0,584493014 |
| HERPUD1 | 0,586969845 |
| ZNF383 | 0,593804135 |
| ZNF506 | 0,594064275 |
| ELL2 | 0,601188411 |
| HSPA13 | 0,626719929 |
| HELB | 0,626944932 |
| UBE2J1 | 0,639279746 |
| ICAM2 | 0,645374304 |
| CCPG1 | 0,652301013 |
| SLC41A2 | 0,6575883 |
| PAM | 0,667039117 |
| RNF24 | 0,670392742 |

| | |
|---|---|
| SRGN | 0,676592286 |
| ANXA2 | 0,680979839 |
| WNT10A | 0,687132146 |
| ZNF784 | 0,702580297 |
| TXNDC11 | 0,733226502 |
| FRRS1 | 0,734485108 |
| ACP2 | 0,736044439 |
| PDK1 | 0,742863075 |
| PDE4DIP | 0,744638134 |
| RNF122 | 0,746093621 |
| DNLZ | 0,785079585 |
| MANEA | 0,79211581 |
| HECA | 0,811469881 |
| APOL6 | 0,831228556 |
| MIR22HG | 0,836387592 |
| PQLC3 | 0,844442345 |
| C15orf65 | 0,873848592 |
| TSTD1 | 0,890080574 |
| CPEB3 | 0,915550811 |
| ACRC | 0,935705487 |
| XBP1 | 0,945013407 |
| MAN1A1 | 0,946047149 |
| TTC22 | 0,956468831 |
| RNF103 | 0,966736689 |
| PIM2 | 0,980442728 |
| TUBB2B | 1,30946E+13 |
| CHST6 | 1,52716E+13 |
| ACOXL | 1,63318E+13 |
| FHDC1 | 1,76623E+13 |
| NUGGC | 1,82082E+13 |
| TYMP | 1,01783E+14 |
| PPP1R32 | 1,05366E+14 |
| FNDC3B | 1,074E+14 |
| SLC26A11 | 1,07688E+14 |
| TICAM2 | 1,08901E+14 |
| SNX16 | 1,12274E+14 |
| ANGPTL2 | 1,18012E+14 |
| ANP32A-IT1 | 1,21275E+14 |
| C12orf74 | 1,2164E+14 |
| GNB4 | 1,26617E+14 |
| BCAS1 | 1,34577E+14 |
| AGAP2-AS1 | 1,38337E+14 |
| LOC101927686 | 1,46109E+14 |
| TTC39A | 1,46274E+14 |
| LOC100507195 | 1,51257E+14 |
| FAAH | 1,55226E+14 |
| SSC4D | 1,55317E+14 |

| | |
|---|---|
| LOXL2 | 1,56941E+14 |
| EDN1 | 1,60079E+14 |
| MIAT | 1,63557E+14 |
| PFN2 | 1,6419E+14 |
| LMTK3 | 1,71736E+14 |
| NEUROG2 | 1,73238E+14 |
| M1AP | 1,74231E+14 |
| EPS8L1 | 1,74988E+14 |
| FAM66B | 1,81452E+14 |
| PVRL4 | 1,81613E+14 |
| DENND2C | 1,84492E+14 |
| POU4F1 | 1,94835E+14 |
| F2R | 2,01601E+14 |
| PTPRO | 2,11842E+14 |
| LOC103908605 | 2,12072E+14 |
| ADAM21 | 2,2514E+14 |
| TRPV3 | 2,2784E+14 |
| MUC20 | 2,34698E+14 |
| NRN1 | 2,36213E+14 |
| LOC102546229 | 2,46023E+14 |
| EMP2 | 2,54E+14 |
| XXYLT1-AS1 | 2,74534E+14 |
| JSRP1 | 2,75519E+14 |
| WNK4 | 2,78956E+14 |
| MYO18B | 2,799E+14 |
| LRRN2 | 2,94599E+14 |
| A2M | 3,04141E+14 |
| TMPRSS3 | 3,12831E+14 |
| PRKD1 | 3,22302E+14 |
| LOC646762 | 3,46586E+14 |
| USP44 | 3,68748E+14 |
| RGPD1 | 3,85216E+14 |
| LDLRAD2 | 4,48837E+14 |
| OVCH1-AS1 | 5,14766E+14 |
| PROSER2-AS1 | 6,66255E+14 |

Supplementary Table 4. Differentially expressed genes (up- and downregulated) in EP cell lines carrying frameshift variants compared to controls.

Down-regulated

| Gene | log2FoldChange |
|---|---|
| PNMAL1 | -8,13026E+14 |
| TDRD12 | -5,93242E+14 |
| TMEM176A | -5,6568E+14 |
| LINC01087 | -5,56304E+14 |
| CD7 | -5,50249E+14 |
| MIR646HG | -5,42462E+14 |
| LOC101926935 | -5,41395E+14 |
| RBPMS | -5,40574E+14 |
| MKRN3 | -5,10486E+14 |
| AOC1 | -5,08035E+14 |
| PLXDC2 | -4,98768E+14 |
| BMP3 | -4,92426E+14 |
| CTNNA2 | -4,90331E+14 |
| WSCD1 | -4,76387E+14 |
| FAM71D | -4,65683E+14 |
| KCNJ15 | -4,47387E+14 |
| OR4C6 | -4,43893E+14 |
| LINC01426 | -4,30409E+14 |
| GLIPR1L2 | -4,29524E+14 |
| DNAH14 | -4,28324E+14 |
| HCK | -4,19139E+14 |
| IL1B | -3,98484E+14 |
| C14orf132 | -3,75539E+14 |
| HIC1 | -3,68464E+14 |
| SNCG | -3,66472E+14 |
| NR2F2 | -3,6382E+14 |
| C2orf91 | -3,6174E+14 |
| SOWAHC | -3,55905E+14 |
| HMX3 | -3,55657E+14 |
| NOXA1 | -3,49006E+14 |
| SPEF2 | -3,44947E+14 |
| CREB3L3 | -3,3534E+14 |
| ANXA3 | -3,3493E+14 |
| GPM6A | -3,29485E+14 |
| CYP1A1 | -3,11335E+14 |
| NSG1 | -3,11244E+14 |
| ADGRE4P | -3,10358E+14 |
| AFAP1L1 | -3,00454E+14 |
| SOBP | -2,9508E+14 |
| FCRL3 | -2,90836E+14 |

| | |
|---|---|
| FILIP1 | -2,89086E+14 |
| EMILIN1 | -2,80664E+14 |
| ROBO1 | -2,73288E+14 |
| FCRL4 | -2,73143E+14 |
| CCL17 | -2,70573E+14 |
| FBN2 | -2,68981E+14 |
| DPP4 | -2,66676E+14 |
| GPR82 | -2,65285E+14 |
| COL16A1 | -2,59969E+14 |
| NAP1L2 | -2,59784E+14 |
| TEKT4P2 | -2,56724E+14 |
| HLA-DRB6 | -2,55334E+14 |
| PMCH | -2,53144E+14 |
| SYCP2 | -2,52283E+14 |
| PGLYRP4 | -2,47335E+14 |
| TOX | -2,44696E+14 |
| TULP2 | -2,43507E+14 |
| MMP9 | -2,3569E+14 |
| PLXNA2 | -2,33748E+14 |
| SORBS2 | -2,27456E+14 |
| HS3ST1 | -2,24173E+14 |
| GATA4 | -2,21829E+14 |
| FAM129B | -2,19585E+14 |
| GRPR | -2,18785E+14 |
| MAN1C1 | -2,12416E+14 |
| MAL | -2,09058E+14 |
| UST | -2,07766E+14 |
| CLEC4A | -2,05511E+14 |
| TUBA8 | -2,05199E+14 |
| HLA-G | -2,01688E+14 |
| KRT7 | -2,00855E+14 |
| OTP | -2,00472E+14 |
| MYO7B | -1,96382E+14 |
| RAB37 | -1,94885E+14 |
| NCALD | -1,91416E+14 |
| NFATC4 | -1,90353E+14 |
| DNASE1L3 | -1,87245E+14 |
| CASKIN2 | -1,81813E+14 |
| LOC100507600 | -1,80249E+14 |
| GUCY1A3 | -1,73926E+14 |
| ACKR3 | -1,72902E+14 |
| GEM | -1,7243E+14 |
| RAB38 | -1,7074E+14 |
| CMTM3 | -1,70214E+14 |
| AMZ1 | -1,65149E+14 |
| HOOK1 | -1,64103E+14 |
| LINC01094 | -1,63915E+14 |

| | |
|---|---|
| PDE6G | -1,63791E+14 |
| DLG4 | -1,59891E+14 |
| LRRIQ3 | -1,59528E+14 |
| GRIN2D | -1,57473E+14 |
| GIMAP6 | -1,56424E+14 |
| FRMD4A | -1,5498E+14 |
| CETP | -1,53291E+14 |
| IL9R | -1,49417E+14 |
| EDARADD | -1,48434E+14 |
| CCDC122 | -1,45743E+14 |
| ZNF362 | -1,43905E+14 |
| SMIM3 | -1,43654E+14 |
| ADAMTS12 | -1,42819E+14 |
| CCDC74B | -1,41299E+14 |
| CDK5R1 | -1,40381E+14 |
| CYP1B1 | -1,36007E+14 |
| ANO5 | -1,34877E+14 |
| TNFSF4 | -1,3046E+14 |
| MGAT4A | -1,30085E+14 |
| PTK2 | -1,29267E+14 |
| EMID1 | -1,28902E+14 |
| KIF26B | -1,28724E+14 |
| MBOAT2 | -1,26436E+14 |
| GPR68 | -1,2405E+14 |
| NRARP | -1,24026E+14 |
| LOC728175 | -1,23893E+14 |
| RIMKLB | -1,22963E+14 |
| CLIC6 | -1,22451E+14 |
| MNS1 | -1,20919E+14 |
| H1F0 | -1,20717E+14 |
| ZGLP1 | -1,12186E+14 |
| CNR1 | -1,11508E+14 |
| ID2 | -1,09568E+14 |
| BHLHE40 | -1,074E+14 |
| METRN | -1,04469E+14 |
| PHC1 | -1,04364E+14 |
| ILDR2 | -1,04008E+14 |
| IL16 | -1,00841E+14 |
| LTA | -1,00806E+14 |
| A4GALT | -1,00798E+14 |
| ANKRD20A12P | -4,30303E+13 |
| ST7-OT4 | -3,73117E+13 |
| IL17RE | -3,43917E+13 |
| PRKCH | -3,26926E+13 |
| GIPC3 | -3,14606E+13 |
| CDH17 | -2,92089E+13 |
| FCGRT | -2,88471E+13 |

| | |
|---|---|
| RPSAP58 | -2,22295E+13 |
| DAPK2 | -2,02859E+13 |
| LOC554206 | -1,82683E+13 |
| FES | -1,80261E+13 |
| MYH10 | -1,70983E+13 |
| SMO | -1,62077E+13 |
| SERPINB10 | -1,39906E+13 |
| TMEM132A | -1,1933E+13 |
| KCND2 | -1,05619E+13 |
| LOC100130298 | -3,26355E+12 |
| PIK3R6 | -0,97731706 |
| TK2 | -0,974380693 |
| DBN1 | -0,970460133 |
| PLA2G4C | -0,95804104 |
| GSAP | -0,935247362 |
| NFATC2 | -0,921688759 |
| STAG3 | -0,91918832 |
| CRACR2B | -0,912071053 |
| LACC1 | -0,89794391 |
| ETS2 | -0,870620006 |
| CDPF1 | -0,866129954 |
| RRN3P1 | -0,863049472 |
| SGK223 | -0,846261608 |
| TNF | -0,840522422 |
| DNAJB5 | -0,833465923 |
| NR2F6 | -0,829751157 |
| LMO7 | -0,825888171 |
| HES1 | -0,822182204 |
| PAAF1 | -0,817397845 |
| IFNGR1 | -0,814399316 |
| EXD3 | -0,811107101 |
| GRAMD3 | -0,796856545 |
| TIMP1 | -0,795848346 |
| IL2RB | -0,788950828 |
| REC8 | -0,7850489 |
| EID2B | -0,776958909 |
| TCEAL3 | -0,771233509 |
| RASSF2 | -0,767291178 |
| ALDH2 | -0,762934624 |
| LY6G5B | -0,756317762 |
| CBLB | -0,747752913 |
| CHD3 | -0,743536851 |
| RGS10 | -0,741653226 |
| CYTH3 | -0,735824403 |
| ABHD6 | -0,727781631 |
| ARMC9 | -0,727243722 |
| DHRS4L2 | -0,719747619 |

| | |
|---|---|
| IRF2BP2 | -0,718136936 |
| AGMAT | -0,713869923 |
| CCDC28B | -0,701482768 |
| TFPT | -0,700782662 |
| CHRNA5 | -0,694944781 |
| FBF1 | -0,692907695 |
| LOC100288798 | -0,691632372 |
| ZNF579 | -0,690777975 |
| PLCXD1 | -0,679839379 |
| PAQR8 | -0,677656828 |
| TCEAL4 | -0,666071621 |
| SAMD10 | -0,661109007 |
| HMGN5 | -0,660315207 |
| TSEN54 | -0,650974712 |
| ENO3 | -0,627472043 |
| TNFSF14 | -0,627095776 |
| RGS14 | -0,612808893 |
| IL27RA | -0,610075512 |
| PAPLN | -0,610045927 |
| DYNC2LI1 | -0,605865069 |
| ZYX | -0,605734265 |
| DFNA5 | -0,591353973 |
| CHST14 | -0,562117689 |
| SGSM2 | -0,560536559 |
| SNX29 | -0,559131507 |
| DHFRL1 | -0,558731672 |
| C7orf60 | -0,548820342 |
| ORAI3 | -0,542068536 |
| TSPYL2 | -0,538576114 |
| ITPK1 | -0,523947744 |
| PCED1A | -0,509294676 |
| NSMF | -0,50892269 |
| ENKD1 | -0,50647274 |
| SVIP | -0,501844564 |
| PABPN1 | -0,496986174 |
| MSANTD2 | -0,489084179 |
| PCBP4 | -0,484517461 |
| SEPT9 | -0,475717041 |
| MAPK12 | -0,468604824 |
| SSBP4 | -0,468154824 |
| C6orf47 | -0,467971865 |
| PIP5K1C | -0,43898694 |
| FAM78A | -0,432060812 |
| SYS1 | -0,424351687 |
| AGAP3 | -0,423414368 |
| PLGRKT | -0,420939534 |
| ZNF234 | -0,41369165 |

| | |
|---|---|
| DAPP1 | -0,407971125 |
| ACADS | -0,404501869 |
| LRRC45 | -0,395588688 |
| PFN1 | -0,388938227 |
| TRAPPC5 | -0,385175031 |
| CALM3 | -0,383137793 |
| CEPT1 | -0,372491989 |
| MOAP1 | -0,366765878 |
| RAI1 | -0,365456397 |
| ZNF747 | -0,364041204 |
| ERCC2 | -0,340860367 |
| TMC6 | -0,335980908 |
| SMARCE1 | -0,325172882 |
| FBXL19 | -0,317709543 |
| STX4 | -0,308278856 |
| TAF15 | -0,306720678 |
| RPS6KA4 | -0,279091063 |
| CARM1 | -0,277411759 |
| PBX2 | -0,275975009 |
| TFEB | -0,272262597 |
| PPP1R18 | -0,271449611 |
| TRAF2 | -0,227926474 |
| MED15 | -0,222251468 |

Up-regulated

| Gene | log2FoldChange |
|---|---|
| SP3 | 0,190769035 |
| CAPZA1 | 0,1979337 |
| PTPN1 | 0,23613938 |
| EZR | 0,243686068 |
| FGFR1OP2 | 0,256394129 |
| ZNF639 | 0,258830824 |
| SYVN1 | 0,266055783 |
| BRPF3 | 0,28399453 |
| RPRD1A | 0,301180111 |
| ZNF562 | 0,302910669 |
| ERO1A | 0,307180226 |
| PNPLA8 | 0,307821568 |
| ATP11C | 0,30891426 |
| PPT1 | 0,319410306 |
| GNA13 | 0,323979658 |
| MSH3 | 0,329159011 |
| CASP3 | 0,345202148 |
| NGLY1 | 0,350582607 |
| CLSTN1 | 0,354648433 |
| SDE2 | 0,362495128 |

| | |
|---|---|
| CASC4 | 0,363484763 |
| TTC37 | 0,380965531 |
| CALU | 0,382180173 |
| ZNF136 | 0,384122644 |
| CLDND1 | 0,394033835 |
| GLCCI1 | 0,398693683 |
| SEC24D | 0,399739208 |
| ELF1 | 0,399812548 |
| ARID2 | 0,409698521 |
| JOSD2 | 0,411871871 |
| LMTK2 | 0,422750971 |
| FAM91A1 | 0,422876253 |
| USP48 | 0,426079334 |
| LIMD1 | 0,436116183 |
| LAMP2 | 0,458095169 |
| HERPUD1 | 0,458497151 |
| TMEM59 | 0,462526785 |
| RAP1B | 0,470067907 |
| GNG7 | 0,47089141 |
| NLK | 0,476124539 |
| LMAN1 | 0,4768773 |
| RNF19A | 0,480257351 |
| CHPF2 | 0,486672707 |
| GNE | 0,491271448 |
| HSP90B1 | 0,494578022 |
| MTRF1L | 0,49932408 |
| ASPHD2 | 0,506343781 |
| KLHL21 | 0,514643918 |
| TRAM1 | 0,525107339 |
| CLIC4 | 0,525504501 |
| TXNRD1 | 0,525813225 |
| C15orf57 | 0,525958174 |
| AFF4 | 0,528554309 |
| SEMA4B | 0,529211218 |
| NDFIP1 | 0,535915056 |
| SEC24A | 0,535987439 |
| POLE4 | 0,539415301 |
| CCPG1 | 0,541198345 |
| IFNLR1 | 0,54648845 |
| GNS | 0,553022331 |
| ICAM2 | 0,56057534 |
| IGF2R | 0,563152733 |
| IL10RA | 0,565274258 |
| TPD52 | 0,5653677 |
| ELL2 | 0,568091756 |
| ENTPD4 | 0,570821382 |
| GCLM | 0,580705187 |

| | |
|---|---|
| CD99L2 | 0,582696818 |
| MBNL2 | 0,584380999 |
| PPP3CC | 0,589952867 |
| PTPN22 | 0,594586432 |
| FAM214A | 0,59592332 |
| UBE2J1 | 0,600524069 |
| CLIP4 | 0,600829063 |
| C16orf54 | 0,603812847 |
| MDM2 | 0,605670475 |
| HSPA13 | 0,616950905 |
| MAP4K3 | 0,633180229 |
| SLC41A2 | 0,643180423 |
| FAM69A | 0,648439813 |
| HELB | 0,650055069 |
| TSTD1 | 0,67283654 |
| CCDC134 | 0,674492078 |
| TXNDC11 | 0,676298574 |
| KIF1B | 0,688050191 |
| FRRS1 | 0,700971823 |
| HECA | 0,707740737 |
| EDEM3 | 0,724216038 |
| RPS18P9 | 0,737417108 |
| ACP2 | 0,745815929 |
| CCDC88A | 0,754318167 |
| ZNF784 | 0,772285329 |
| ERC1 | 0,773127477 |
| BACH1 | 0,774148579 |
| PRDM1 | 0,781737861 |
| PAM | 0,786873518 |
| PDK1 | 0,789907592 |
| DERL3 | 0,801829739 |
| MANEA | 0,803452733 |
| BCAS1 | 0,805571235 |
| RRAGD | 0,838170322 |
| MAN2A1 | 0,846587198 |
| MXI1 | 0,856660721 |
| XBP1 | 0,879555788 |
| MAN1A1 | 0,884890023 |
| RNF103 | 0,891837844 |
| NR1D2 | 0,895212807 |
| TYMP | 0,899483678 |
| ATXN7L1 | 0,912810828 |
| SLC26A11 | 0,916000465 |
| ERN1 | 0,98818956 |
| CPEB3 | 0,994378547 |
| SOCS2 | 1,0279E+13 |
| TTC39A | 2,13298E+13 |

| | |
|---|---|
| A2M | 3,42779E+13 |
| FNDC3B | 1,02244E+14 |
| SRXN1 | 1,02954E+14 |
| KCNA3 | 1,04621E+14 |
| KCNK6 | 1,05016E+14 |
| STAC3 | 1,0983E+14 |
| TUBB2B | 1,10287E+14 |
| FOSL2 | 1,12307E+14 |
| SNX16 | 1,25076E+14 |
| CFAP54 | 1,25514E+14 |
| FAAH | 1,29942E+14 |
| CUEDC1 | 1,30957E+14 |
| GNB4 | 1,35185E+14 |
| TMEM169 | 1,40502E+14 |
| C12orf74 | 1,40726E+14 |
| KIAA1671 | 1,45204E+14 |
| DSG2 | 1,46554E+14 |
| CES4A | 1,4667E+14 |
| POU4F1 | 1,50725E+14 |
| TRPV3 | 1,67222E+14 |
| MIAT | 1,67313E+14 |
| EPS8L1 | 1,72755E+14 |
| PFN2 | 1,80733E+14 |
| ACOXL | 1,81003E+14 |
| IFNG-AS1 | 1,96845E+14 |
| HMOX1 | 1,97876E+14 |
| NEUROG2 | 1,98359E+14 |
| RAB3B | 2,01046E+14 |
| EMP2 | 2,06236E+14 |
| NRN1 | 2,06247E+14 |
| GAS6 | 2,1093E+14 |
| ZNF208 | 2,18514E+14 |
| KIF5C | 2,21651E+14 |
| TMPRSS3 | 2,59201E+14 |
| SHTN1 | 2,59765E+14 |
| ADAM21 | 2,69173E+14 |
| SIX1 | 2,96739E+14 |
| SIX3 | 3,01118E+14 |
| C8orf31 | 3,05596E+14 |
| LRRN2 | 3,13726E+14 |
| ZNF311 | 3,18272E+14 |
| PRKD1 | 3,32053E+14 |
| COL1A2 | 3,50796E+14 |
| LOC646762 | 3,67762E+14 |
| MSR1 | 5,3289E+14 |
| PROSER2-AS1 | 6,39965E+14 |

Supplementary Table 5. Differentially expressed genes (up- and downregulated) in EP cell lines carrying missense variants compared to controls.

Down-regulated

| Gene | log2FoldChange |
|---|---|
| LCN8 | -6,15549E+14 |
| TRIM31 | -5,48946E+14 |
| SNCA | -5,43678E+14 |
| BCHE | -4,86024E+14 |
| STMND1 | -4,77016E+14 |
| CRTAM | -4,51216E+14 |
| HMX3 | -4,46263E+14 |
| OR4C6 | -4,43755E+14 |
| CTNNA2 | -4,24047E+14 |
| FKBP10 | -4,23994E+14 |
| RNF217 | -3,94098E+14 |
| HIC1 | -3,80143E+14 |
| NRP1 | -3,56545E+14 |
| OSBPL10 | -3,47246E+14 |
| MYO7B | -3,46769E+14 |
| C14orf132 | -3,37102E+14 |
| ANKRD18A | -3,36782E+14 |
| LOC100130298 | -3,3651E+14 |
| CREB5 | -3,32466E+14 |
| BMP4 | -3,2767E+14 |
| TPD52L1 | -3,27095E+14 |
| GPM6A | -3,20539E+14 |
| PDE1C | -3,18292E+14 |
| TUSC1 | -3,11627E+14 |
| ADGRA3 | -3,07795E+14 |
| FAM201A | -2,99978E+14 |
| RND3 | -2,99939E+14 |
| MYRF | -2,89436E+14 |
| PLXNA2 | -2,89244E+14 |
| MAN1C1 | -2,86863E+14 |
| VIL1 | -2,80528E+14 |
| PRKCH | -2,72996E+14 |
| NPIPA5 | -2,69786E+14 |
| APBB1 | -2,62601E+14 |
| MAST4 | -2,59738E+14 |
| DISP2 | -2,48637E+14 |
| EIF5AL1 | -2,44662E+14 |
| LOC729737 | -2,35069E+14 |
| TRPC1 | -2,33533E+14 |
| ANKRD36BP2 | -2,31329E+14 |

| | |
|---|---|
| ADGRE2 | -2,31183E+14 |
| DPP4 | -2,1843E+14 |
| NGFRAP1 | -2,16016E+14 |
| SORBS2 | -2,11036E+14 |
| C1orf54 | -2,04756E+14 |
| NHSL1 | -2,03113E+14 |
| KCND2 | -1,79905E+14 |
| AMZ1 | -1,78121E+14 |
| PTK2 | -1,78038E+14 |
| ROBO1 | -1,75476E+14 |
| PYGL | -1,73809E+14 |
| PCGF2 | -1,72379E+14 |
| CRYM | -1,69587E+14 |
| TNFRSF19 | -1,64044E+14 |
| GRPR | -1,63431E+14 |
| LYPD6B | -1,63069E+14 |
| GPRC5C | -1,62255E+14 |
| HNF4G | -1,62231E+14 |
| SDC3 | -1,61709E+14 |
| MICAL3 | -1,58098E+14 |
| SEPN1 | -1,55015E+14 |
| CLIC6 | -1,54104E+14 |
| SERINC2 | -1,5244E+14 |
| BHLHE22 | -1,50964E+14 |
| ANKRD34A | -1,36636E+14 |
| IL6R | -1,27178E+14 |
| ERICH5 | -1,21828E+14 |
| TRERF1 | -1,13436E+14 |
| GUCY1A3 | -1,11778E+14 |
| NFATC2 | -1,07335E+14 |
| BHLHE41 | -1,06532E+14 |
| LOC613266 | -1,05817E+14 |
| RNF144A | -1,01557E+14 |
| TIMP1 | -1,0024E+14 |
| MIR646HG | -4,76648E+13 |
| ITGA5 | -3,13912E+13 |
| SMO | -1,49684E+13 |
| PON2 | -0,989368902 |
| VANGL1 | -0,927698308 |
| DHRS4L2 | -0,888381534 |
| ST3GAL1 | -0,886256256 |
| ETS2 | -0,860179321 |
| ZMIZ1 | -0,820657289 |
| HMGN5 | -0,802059482 |
| PFKFB3 | -0,791483293 |
| ARHGEF5 | -0,775704715 |
| VCL | -0,773092785 |

| | |
|---|---|
| NCKAP1 | -0,758736001 |
| ARHGAP18 | -0,724148458 |
| IKZF2 | -0,718496959 |
| LMO7 | -0,717021806 |
| FAM86DP | -0,714572401 |
| CHD3 | -0,691170166 |
| AGAP3 | -0,686363679 |
| PSMD5-AS1 | -0,673754068 |
| FCGR2B | -0,645643745 |
| SMC1A | -0,637159742 |
| ERMP1 | -0,62070133 |
| BRD3 | -0,603492425 |
| CTH | -0,582570933 |
| ZNF589 | -0,542011207 |
| UNC119B | -0,525044193 |
| TNRC18 | -0,516573269 |
| UHRF1 | -0,514328742 |
| SVIP | -0,51429402 |
| SCFD2 | -0,510323389 |
| KIF14 | -0,508264381 |
| TPCN1 | -0,508003507 |
| BUB1B | -0,493982635 |
| IRF2BPL | -0,493889937 |
| TEX2 | -0,485588553 |
| FAM78A | -0,483621595 |
| SORL1 | -0,482842165 |
| CDC42EP3 | -0,473079101 |
| SKP2 | -0,470595566 |
| RAI1 | -0,468816976 |
| MCM6 | -0,455934365 |
| TRIP6 | -0,446988078 |
| SMC2 | -0,443032895 |
| PKP4 | -0,427003003 |
| TOP2A | -0,422098536 |
| TCOF1 | -0,418061745 |
| DIAPH3 | -0,414764091 |
| UCK2 | -0,411688834 |
| HMGXB4 | -0,411118669 |
| THADA | -0,404561755 |
| NCAPD3 | -0,384078127 |
| WDR62 | -0,383888238 |
| C15orf39 | -0,360846884 |
| SEPT11 | -0,351914863 |
| DCAF7 | -0,319069805 |
| SRRM1 | -0,273372949 |
| ILF3 | -0,262801058 |

Up-regulated

| Gene | log2FoldChange |
|---|---|
| TMED9 | 0,326063746 |
| P4HB | 0,363157414 |
| B2M | 0,413930347 |
| IL2RG | 0,442823991 |
| PTRHD1 | 0,453696987 |
| SKAP1 | 0,454528208 |
| GLRX | 0,456872921 |
| PGD | 0,457509852 |
| PI4K2B | 0,460693369 |
| CINP | 0,468973591 |
| GSTZ1 | 0,47780936 |
| EHMT1 | 0,48616495 |
| ZNF107 | 0,50476354 |
| PTPRC | 0,520318866 |
| FAM117A | 0,524806153 |
| PPP3CA | 0,545432944 |
| F11R | 0,55767147 |
| IQSEC1 | 0,558080954 |
| POLE4 | 0,560400363 |
| SEMA4A | 0,564425205 |
| EVI2B | 0,610880226 |
| C10orf32 | 0,633357439 |
| CDKN1A | 0,634024116 |
| LY9 | 0,6433409 |
| NAPRT | 0,657625805 |
| CYTIP | 0,675794916 |
| C4orf32 | 0,690537066 |
| FRRS1 | 0,7005104 |
| PITRM1 | 0,712581491 |
| ACYP2 | 0,713724707 |
| ZNF682 | 0,717946269 |
| ISCU | 0,719630924 |
| ZNF506 | 0,721453343 |
| WDR25 | 0,733063774 |
| HHAT | 0,73462183 |
| C15orf57 | 0,743316333 |
| NDNL2 | 0,757584994 |
| ARSA | 0,763401722 |
| CD99L2 | 0,778965538 |
| DERL3 | 0,796947154 |
| SIDT1 | 0,804266044 |
| SRGN | 0,804513057 |
| AP1S3 | 0,811854271 |
| TARSL2 | 0,826935162 |

| | |
|---|---|
| HECA | 0,834301168 |
| SERPINB9 | 0,836941016 |
| MS4A7 | 0,850784552 |
| PMAIP1 | 0,851585875 |
| HMCES | 0,864630413 |
| TMSB10 | 0,882364649 |
| MIR22HG | 0,889422535 |
| LY96 | 0,902300753 |
| SPATA20 | 0,939447315 |
| LGMN | 0,974627836 |
| UNC13B | 0,996313088 |
| RPS6KL1 | 1,62732E+12 |
| SLC26A11 | 1,16168E+13 |
| FAM83H | 1,1619E+13 |
| FADS3 | 1,24342E+13 |
| ADAM19 | 1,38572E+13 |
| PLEKHG1 | 1,38735E+13 |
| SERPINB1 | 1,53982E+13 |
| LOXL2 | 1,87985E+13 |
| SMA4 | 1,92779E+13 |
| ATAD3C | 2,01563E+13 |
| PTGER4 | 2,04475E+13 |
| CHST6 | 2,07198E+13 |
| PVRL4 | 2,62873E+13 |
| CPEB1 | 3,02677E+13 |
| LAMP5 | 3,78288E+13 |
| INPP5J | 3,85247E+13 |
| OVCH1-AS1 | 4,32124E+13 |
| ATF3 | 1,02836E+14 |
| SDSL | 1,06406E+14 |
| BLVRA | 1,06679E+14 |
| TSTD1 | 1,07382E+14 |
| GNB4 | 1,08172E+14 |
| CPEB3 | 1,10102E+14 |
| SOCS1 | 1,13021E+14 |
| ABCA3 | 1,15779E+14 |
| BMS1P20 | 1,1644E+14 |
| TYMP | 1,18224E+14 |
| CDKN2A | 1,18804E+14 |
| SAT1 | 1,19362E+14 |
| GAS7 | 1,21193E+14 |
| GSN | 1,21443E+14 |
| GAL3ST4 | 1,24229E+14 |
| PQLC3 | 1,24355E+14 |
| PIM2 | 1,24367E+14 |
| XXYLT1-AS2 | 1,26515E+14 |
| P2RX5 | 1,30409E+14 |

| | |
|---|---|
| INSIG1 | 1,31503E+14 |
| PLK2 | 1,37409E+14 |
| TTC22 | 1,39804E+14 |
| MMP25-AS1 | 1,44041E+14 |
| GTF2IRD2 | 1,48468E+14 |
| PLEKHB1 | 1,53831E+14 |
| HCAR3 | 1,54723E+14 |
| CEP70 | 1,54827E+14 |
| PTPRO | 1,58182E+14 |
| PLTP | 1,5823E+14 |
| CCL25 | 1,60507E+14 |
| ADAP2 | 1,65787E+14 |
| CELSR1 | 1,67687E+14 |
| FAM90A1 | 1,70748E+14 |
| ACOXL | 1,71269E+14 |
| TUBB2B | 1,71967E+14 |
| CEACAM1 | 1,73037E+14 |
| ADCY9 | 1,79613E+14 |
| CSPG4 | 1,80338E+14 |
| FCER1G | 1,85477E+14 |
| MUC20 | 1,9076E+14 |
| FAAH | 1,92146E+14 |
| GAB3 | 1,93697E+14 |
| SH2D4A | 1,98416E+14 |
| ZP3 | 2,0445E+14 |
| MOXD1 | 2,07397E+14 |
| TYW1B | 2,10478E+14 |
| LMTK3 | 2,10822E+14 |
| MT2A | 2,10939E+14 |
| HOMER2 | 2,1101E+14 |
| SYNPO2 | 2,12295E+14 |
| CLEC2B | 2,13951E+14 |
| TLDC2 | 2,15392E+14 |
| HAPLN3 | 2,22016E+14 |
| UNC13C | 2,23797E+14 |
| PER3 | 2,37483E+14 |
| PTPRN2 | 2,41422E+14 |
| ST5 | 2,43321E+14 |
| LINC01150 | 2,45415E+14 |
| XIRP1 | 2,48852E+14 |
| CCND1 | 2,52838E+14 |
| S100A10 | 2,54653E+14 |
| ST14 | 2,56201E+14 |
| CES4A | 2,6115E+14 |
| IFNG | 2,62545E+14 |
| RORA | 2,63154E+14 |
| AMBP | 2,647E+14 |

| | |
|---|---|
| POMC | 2,66089E+14 |
| EMP2 | 2,67399E+14 |
| B4GALNT3 | 2,68904E+14 |
| ASMT | 2,80934E+14 |
| POU4F1 | 2,81455E+14 |
| MT1E | 2,83162E+14 |
| EID3 | 2,87693E+14 |
| PRKCZ | 2,9275E+14 |
| FAM189A1 | 2,99654E+14 |
| TRPV3 | 3,00969E+14 |
| EPB41L4A | 3,10602E+14 |
| LINC00987 | 3,13081E+14 |
| ST6GALNAC2 | 3,28424E+14 |
| EEF1A2 | 3,63054E+14 |
| IL12B | 3,99896E+14 |
| LINC00908 | 4,02194E+14 |
| SOX18 | 4,06801E+14 |
| MYO18B | 4,85801E+14 |
| LAMP5-AS1 | 4,89051E+14 |
| LIX1 | 5,39895E+14 |
| CLEC4C | 5,42295E+14 |
| AKR1C3 | 5,56017E+14 |
| USP44 | 6,24218E+14 |
| ACTN3 | 6,4765E+14 |
| PROSER2-AS1 | 6,6128E+14 |

Supplementary Table 6. Differentially expressed genes (up- and downregulated) in EP cell lines carrying nonsense variants compared to controls.

Down-regulated

| Gene | log2FoldChange |
|---|---|
| PLXDC2 | -7,64409E+14 |
| TSPAN18 | -6,94157E+14 |
| PCDH7 | -6,93184E+14 |
| PLS3 | -6,44279E+14 |
| NRG3 | -6,43486E+14 |
| TBX20 | -6,34309E+14 |
| RPRM | -6,29361E+14 |
| UGT2A3 | -6,22747E+14 |
| INSM2 | -6,20868E+14 |
| WSCD1 | -5,94135E+14 |
| CADPS | -5,76505E+14 |
| CLDN10 | -5,43758E+14 |
| KCNJ3 | -5,42473E+14 |
| FLRT3 | -5,31061E+14 |
| ITGA11 | -5,27685E+14 |
| KCNJ2 | -5,26937E+14 |
| ST8SIA1 | -5,22884E+14 |
| SDPR | -5,22847E+14 |
| FAM86B1 | -5,15565E+14 |
| LINC01605 | -5,10901E+14 |
| TNIP3 | -5,05826E+14 |
| KCNJ12 | -4,93469E+14 |
| PRKCQ-AS1 | -4,91508E+14 |
| MYT1 | -4,80464E+14 |
| GAREM | -4,77918E+14 |
| EDIL3 | -4,68892E+14 |
| DLX1 | -4,63926E+14 |
| MMP7 | -4,62985E+14 |
| SLC39A12 | -4,61779E+14 |
| FRMPD3 | -4,55582E+14 |
| NTN4 | -4,45707E+14 |
| IRX6 | -4,37299E+14 |
| LINC01320 | -4,30616E+14 |
| ROBO3 | -4,12137E+14 |
| CDC42BPA | -4,04076E+14 |
| FAXDC2 | -4,02225E+14 |
| SBSN | -3,97632E+14 |
| LURAP1 | -3,94779E+14 |
| CTNNA2 | -3,92623E+14 |
| FAM198B | -3,91051E+14 |

| | |
|---|---|
| ACPP | -3,9002E+14 |
| OTOGL | -3,89295E+14 |
| MTL5 | -3,89153E+14 |
| GPC4 | -3,85801E+14 |
| SHANK2 | -3,76602E+14 |
| PCP4L1 | -3,72768E+14 |
| TOX | -3,68549E+14 |
| CFTR | -3,66814E+14 |
| RBPMS | -3,66638E+14 |
| PRKCH | -3,59192E+14 |
| SSC5D | -3,58172E+14 |
| PALLD | -3,50285E+14 |
| PMCH | -3,47724E+14 |
| MYH10 | -3,4716E+14 |
| GPR87 | -3,45042E+14 |
| SCD5 | -3,35312E+14 |
| MMP9 | -3,352E+14 |
| PDE4C | -3,24505E+14 |
| MYRIP | -3,22165E+14 |
| NRXN3 | -3,19697E+14 |
| INTU | -3,157E+14 |
| CR1 | -3,13597E+14 |
| EDARADD | -3,13397E+14 |
| PCDHGA5 | -3,1327E+14 |
| PHOSPHO1 | -3,12785E+14 |
| RPSAP58 | -3,07708E+14 |
| NAP1L3 | -3,06572E+14 |
| C2orf78 | -3,01822E+14 |
| HOXC5 | -2,99701E+14 |
| CNKSR2 | -2,97849E+14 |
| CASP14 | -2,96413E+14 |
| ABCC9 | -2,9576E+14 |
| CR2 | -2,85446E+14 |
| SH3RF3 | -2,85297E+14 |
| COLEC12 | -2,82881E+14 |
| LOC101927815 | -2,82424E+14 |
| MYO1F | -2,79807E+14 |
| LGR4 | -2,79618E+14 |
| SEMA5A | -2,75537E+14 |
| PCDHGB6 | -2,73968E+14 |
| OTX1 | -2,72894E+14 |
| HOXB6 | -2,72771E+14 |
| FAM89A | -2,71954E+14 |
| PTAFR | -2,61349E+14 |
| HS3ST1 | -2,58671E+14 |
| ID2 | -2,53349E+14 |
| MAK | -2,4839E+14 |

| | |
|---|---|
| TPM2 | -2,41021E+14 |
| IER5L | -2,38709E+14 |
| CMTM7 | -2,37734E+14 |
| MS4A14 | -2,35437E+14 |
| POU3F1 | -2,35381E+14 |
| GIMAP7 | -2,35073E+14 |
| TPBG | -2,35014E+14 |
| PDE1C | -2,33485E+14 |
| ACTN1 | -2,33227E+14 |
| MYOF | -2,32926E+14 |
| MARVELD1 | -2,29832E+14 |
| WWTR1 | -2,29652E+14 |
| CD244 | -2,26241E+14 |
| RAB37 | -2,24917E+14 |
| GIMAP1 | -2,24207E+14 |
| C15orf27 | -2,21748E+14 |
| SPINK2 | -2,21713E+14 |
| EPHB2 | -2,19775E+14 |
| DHRS3 | -2,18652E+14 |
| C17orf51 | -2,16719E+14 |
| LRRC16A | -2,15154E+14 |
| PCDHGB2 | -2,14676E+14 |
| COL16A1 | -2,13263E+14 |
| RCOR2 | -2,10961E+14 |
| PARD6G | -2,10894E+14 |
| IL2RA | -2,0989E+14 |
| CROCC | -2,08758E+14 |
| CXXC4 | -2,07925E+14 |
| GRHL3 | -2,03897E+14 |
| OR2T3 | -2,03013E+14 |
| SIX4 | -2,02701E+14 |
| PTGER2 | -2,02597E+14 |
| SNX21 | -2,01999E+14 |
| GPR153 | -1,99667E+14 |
| HOXB7 | -1,99335E+14 |
| GPR68 | -1,97819E+14 |
| ARHGEF17 | -1,97582E+14 |
| PEX5L | -1,97298E+14 |
| FCRL4 | -1,97125E+14 |
| SCARF1 | -1,96395E+14 |
| ZNF608 | -1,95891E+14 |
| WDR17 | -1,94972E+14 |
| C1orf106 | -1,94495E+14 |
| TRO | -1,92878E+14 |
| MCC | -1,92241E+14 |
| HDAC11 | -1,91184E+14 |
| P2RY1 | -1,90853E+14 |

| | |
|---|---|
| LRP1 | -1,87739E+14 |
| CALCRL | -1,85295E+14 |
| TPM1 | -1,80896E+14 |
| TLN2 | -1,79526E+14 |
| CDKL5 | -1,79495E+14 |
| ZBTB47 | -1,77898E+14 |
| TBC1D8B | -1,72797E+14 |
| TGM2 | -1,72654E+14 |
| BMP1 | -1,70568E+14 |
| ZNF469 | -1,66444E+14 |
| MNX1 | -1,64931E+14 |
| PI4KAP1 | -1,64229E+14 |
| MAP3K4 | -1,63864E+14 |
| LOC102724094 | -1,62726E+14 |
| TP53BP2 | -1,61312E+14 |
| MEX3A | -1,61304E+14 |
| METRN | -1,60957E+14 |
| RABL2A | -1,60936E+14 |
| BEND4 | -1,5988E+14 |
| GOLGA2P7 | -1,59563E+14 |
| PHC1 | -1,58781E+14 |
| CABYR | -1,58458E+14 |
| EPHA2 | -1,57883E+14 |
| FGFR1 | -1,56999E+14 |
| TGFBR2 | -1,56898E+14 |
| CSPG4 | -1,56231E+14 |
| PCGF2 | -1,55742E+14 |
| IRAK2 | -1,54285E+14 |
| MERTK | -1,52935E+14 |
| PIK3IP1 | -1,52365E+14 |
| PRDM11 | -1,51215E+14 |
| SIGLEC10 | -1,49539E+14 |
| EPB41L5 | -1,49476E+14 |
| ZNF205 | -1,47659E+14 |
| CCDC136 | -1,47369E+14 |
| SDC4 | -1,47273E+14 |
| LRRC49 | -1,47179E+14 |
| CREB5 | -1,47059E+14 |
| THBS3 | -1,44856E+14 |
| LAMP3 | -1,44123E+14 |
| MNS1 | -1,43735E+14 |
| CCDC92 | -1,43034E+14 |
| GRAMD1B | -1,42249E+14 |
| KIAA0754 | -1,41587E+14 |
| KIF16B | -1,41191E+14 |
| LMO2 | -1,40974E+14 |
| CFAP57 | -1,40464E+14 |

| | |
|---|---|
| VWCE | -1,39734E+14 |
| RP9P | -1,3947E+14 |
| TNFSF12 | -1,38935E+14 |
| STRIP2 | -1,37686E+14 |
| MRC2 | -1,37336E+14 |
| L3MBTL4 | -1,36607E+14 |
| CASKIN2 | -1,35982E+14 |
| NFATC2 | -1,35493E+14 |
| C15orf62 | -1,35336E+14 |
| C1orf198 | -1,35297E+14 |
| NRG4 | -1,35047E+14 |
| LINC00877 | -1,33476E+14 |
| ADM5 | -1,32856E+14 |
| CCL22 | -1,32626E+14 |
| SLC16A10 | -1,3242E+14 |
| KIF3C | -1,32346E+14 |
| TTC12 | -1,32158E+14 |
| KIF26B | -1,32148E+14 |
| CDC14A | -1,31899E+14 |
| SCML1 | -1,31183E+14 |
| GIPC1 | -1,30456E+14 |
| WDR63 | -1,2969E+14 |
| C2orf74 | -1,29052E+14 |
| ILDR2 | -1,28488E+14 |
| BMI1 | -1,28435E+14 |
| PSTPIP2 | -1,27324E+14 |
| SOX9 | -1,27071E+14 |
| LOC642361 | -1,26397E+14 |
| TRIM6 | -1,2582E+14 |
| SLC25A53 | -1,23475E+14 |
| SAPCD2 | -1,2343E+14 |
| SLC25A29 | -1,22089E+14 |
| DTX4 | -1,21859E+14 |
| FAM109A | -1,20525E+14 |
| LRRC56 | -1,1969E+14 |
| LINC00926 | -1,19551E+14 |
| ITGA3 | -1,19183E+14 |
| WWC3 | -1,17867E+14 |
| ACER2 | -1,17813E+14 |
| AGRN | -1,17564E+14 |
| PPP1R13B | -1,17456E+14 |
| GBE1 | -1,17166E+14 |
| PKP2 | -1,16967E+14 |
| TTYH3 | -1,16953E+14 |
| XRRA1 | -1,16294E+14 |
| ATP6AP1L | -1,1622E+14 |
| TRIB2 | -1,15771E+14 |

| | |
|---|---|
| FBXO44 | -1,1567E+14 |
| MYL9 | -1,15242E+14 |
| CBX2 | -1,14855E+14 |
| AIM1 | -1,14599E+14 |
| PCTP | -1,13648E+14 |
| PTK2 | -1,13352E+14 |
| IFIT2 | -1,13128E+14 |
| DHX58 | -1,12787E+14 |
| GAMT | -1,12072E+14 |
| PPFIBP1 | -1,1156E+14 |
| SLC19A2 | -1,11113E+14 |
| MYLIP | -1,11002E+14 |
| ETV4 | -1,1076E+14 |
| TSC22D3 | -1,10489E+14 |
| ETV5 | -1,10459E+14 |
| PVR | -1,10172E+14 |
| TTC39C | -1,09539E+14 |
| SNN | -1,09532E+14 |
| ASRGL1 | -1,08787E+14 |
| YY2 | -1,08532E+14 |
| OCLN | -1,07124E+14 |
| FAM65A | -1,06953E+14 |
| PTMS | -1,05945E+14 |
| NID1 | -1,05911E+14 |
| ITGB2-AS1 | -1,05516E+14 |
| LGALS14 | -1,0467E+14 |
| ROGDI | -1,04379E+14 |
| FAM86C2P | -1,04261E+14 |
| SYNGAP1 | -1,04106E+14 |
| ENO3 | -1,0361E+14 |
| ABTB2 | -1,0212E+14 |
| BCAT1 | -1,01847E+14 |
| PAG1 | -1,01107E+14 |
| FAM86DP | -1,0104E+14 |
| CHD3 | -1,00712E+14 |
| KSR1 | -1,00446E+14 |
| PITX2 | -6,75939E+13 |
| PRKG2 | -5,83049E+13 |
| OLFML2A | -5,08366E+13 |
| CACNA2D1 | -4,25281E+13 |
| PTK7 | -4,18247E+13 |
| GRB7 | -3,69162E+13 |
| ZNF467 | -3,65499E+13 |
| RBFOX2 | -3,02363E+13 |
| SLC35G2 | -2,94821E+13 |
| FSD1 | -2,81519E+13 |
| FILIP1 | -2,68771E+13 |

| | |
|---|---|
| DLG4 | -2,28159E+13 |
| FGFR4 | -2,265E+13 |
| CYP1A1 | -2,21372E+13 |
| RTN4RL2 | -2,11146E+13 |
| NFAM1 | -1,83983E+13 |
| PHLDA1 | -1,81166E+13 |
| MYBL1 | -1,74107E+13 |
| SNPH | -1,70321E+13 |
| NPNT | -1,66213E+13 |
| CCDC74A | -1,56851E+13 |
| DLG5 | -1,53632E+13 |
| SPR | -1,50529E+13 |
| FOXO3 | -1,48775E+13 |
| PARM1 | -1,47584E+13 |
| LOC729737 | -1,43645E+13 |
| TBKBP1 | -1,29296E+13 |
| SMIM3 | -1,24775E+13 |
| EMX1 | -1,22388E+13 |
| MFGE8 | -1,17945E+13 |
| BCL9L | -1,13904E+13 |
| CPNE4 | -4,92256E+12 |
| MREG | -0,998271446 |
| F8 | -0,997504347 |
| CCL5 | -0,99364316 |
| HMGN5 | -0,986190892 |
| REC8 | -0,986041419 |
| MAPKAPK3 | -0,985384649 |
| PAQR8 | -0,980574114 |
| LOC257396 | -0,978412484 |
| PCNXL2 | -0,967552672 |
| CNR2 | -0,949136207 |
| ITPR1 | -0,94832192 |
| MARCKSL1 | -0,947521229 |
| CLEC17A | -0,947505037 |
| SOX12 | -0,946638117 |
| KIF21A | -0,942821758 |
| LPIN1 | -0,942437944 |
| PNMA1 | -0,940695025 |
| MARVELD2 | -0,935966895 |
| TMEM198B | -0,929038761 |
| PTP4A3 | -0,916721091 |
| DYNC2LI1 | -0,909668926 |
| OCEL1 | -0,896835815 |
| MAP3K12 | -0,896278233 |
| YBX3 | -0,887094201 |
| DUSP16 | -0,884941652 |
| SIPA1L1 | -0,884619688 |

| | |
|---|---|
| RALGAPA2 | -0,884402297 |
| DCAF4 | -0,881369558 |
| HOXB4 | -0,880170956 |
| PSMD5-AS1 | -0,874522949 |
| MFI2 | -0,874411889 |
| SEMA7A | -0,873465369 |
| NEIL2 | -0,869206502 |
| KIF13A | -0,865246364 |
| CD22 | -0,854083973 |
| WDR19 | -0,850911976 |
| HAAO | -0,843597236 |
| RNF144A | -0,837906528 |
| IL16 | -0,830602351 |
| QSOX1 | -0,828599075 |
| PHLDB3 | -0,826302038 |
| STK33 | -0,82303391 |
| TET3 | -0,822926396 |
| NOL4L | -0,8215099 |
| IFT81 | -0,810138158 |
| ZNF446 | -0,808157401 |
| DYRK1B | -0,805568922 |
| CD55 | -0,804852469 |
| LUZP1 | -0,798447388 |
| RDX | -0,796201499 |
| DRAM1 | -0,787740704 |
| PACS2 | -0,787162242 |
| RALB | -0,784911039 |
| CHST14 | -0,782585327 |
| CHDH | -0,781951684 |
| ECHDC2 | -0,781684253 |
| UBXN7 | -0,780159519 |
| LINC01278 | -0,777713339 |
| ZFAS1 | -0,76949832 |
| LOC100506746 | -0,768956423 |
| SARM1 | -0,765953867 |
| FMNL3 | -0,760778183 |
| SIDT2 | -0,757495762 |
| TCEAL4 | -0,752840947 |
| ELOVL6 | -0,744665391 |
| SLC1A1 | -0,744317361 |
| CNNM4 | -0,743549185 |
| HACD2 | -0,740273087 |
| LTBP4 | -0,737256739 |
| PIDD1 | -0,736483183 |
| FAM213B | -0,732721156 |
| RBM38 | -0,732701447 |
| PHF14 | -0,729520901 |

| | |
|---|---|
| ARHGAP21 | -0,725232845 |
| ZNF496 | -0,722790373 |
| HDGFRP3 | -0,722107163 |
| SLX4IP | -0,722080336 |
| MAPK12 | -0,719155094 |
| TFAP4 | -0,718372872 |
| NUB1 | -0,715134502 |
| TRAF3IP1 | -0,713092843 |
| MIPEP | -0,711045976 |
| LRRC20 | -0,709243648 |
| NUTM2B-AS1 | -0,708292569 |
| RCSD1 | -0,706896122 |
| TNIP1 | -0,706863119 |
| CRAMP1L | -0,70483775 |
| ICK | -0,704529422 |
| CEPT1 | -0,69960848 |
| STK36 | -0,692086668 |
| TULP3 | -0,69120109 |
| GALNT7 | -0,688709468 |
| ARID5A | -0,686489957 |
| ZNRF1 | -0,685511395 |
| RRM2B | -0,684686854 |
| TTC27 | -0,684175518 |
| IRF8 | -0,679170815 |
| ITPKB | -0,677045778 |
| IFT172 | -0,674713855 |
| RHOB | -0,674407199 |
| TOB1 | -0,673805193 |
| SNX22 | -0,673783188 |
| MFHAS1 | -0,671813598 |
| JAK1 | -0,671354858 |
| MOSPD3 | -0,670813691 |
| PAQR7 | -0,668850695 |
| RABL2B | -0,666063013 |
| NFIC | -0,665680983 |
| TGFBRAP1 | -0,665298716 |
| TM7SF3 | -0,664046676 |
| CRTC1 | -0,663549386 |
| USP40 | -0,661944749 |
| ERF | -0,661698985 |
| FAM212B | -0,661651955 |
| STK3 | -0,660742212 |
| KDM6B | -0,658469705 |
| PEX26 | -0,655514237 |
| DNAJB5 | -0,655402667 |
| ZNF827 | -0,654871164 |
| SGSM2 | -0,65321742 |

| | |
|---|---|
| LCP2 | -0,651464918 |
| ETV6 | -0,650826425 |
| KLF9 | -0,650553923 |
| CPOX | -0,647952889 |
| LDLRAD4 | -0,647344858 |
| PCED1A | -0,647320296 |
| TRIM22 | -0,646817394 |
| ROCK2 | -0,646391902 |
| CSNK1G1 | -0,644233428 |
| LSP1 | -0,643545262 |
| PHLDA3 | -0,641487439 |
| LIX1L | -0,641042582 |
| F8A1 | -0,639199397 |
| ZNF609 | -0,638035302 |
| LYSMD2 | -0,637602697 |
| ZBED6CL | -0,63719646 |
| MBOAT7 | -0,631499765 |
| GNA12 | -0,628127874 |
| TMEM159 | -0,626079823 |
| RALGDS | -0,625852877 |
| BRF1 | -0,620555691 |
| ASB1 | -0,61868453 |
| PPM1F | -0,616523356 |
| HDGFRP2 | -0,6160822 |
| HEXIM1 | -0,610565446 |
| TPCN1 | -0,609146744 |
| LYRM4 | -0,607095759 |
| GADD45B | -0,606449947 |
| ZBED1 | -0,603585552 |
| ABI2 | -0,602695715 |
| VPS41 | -0,602522312 |
| SUSD6 | -0,602001439 |
| MSC | -0,601497324 |
| ARHGAP35 | -0,599582221 |
| PRKAB1 | -0,599422277 |
| MLLT10 | -0,598370022 |
| NUDT16 | -0,598348874 |
| NPIPB5 | -0,597790673 |
| MDFIC | -0,597383635 |
| TNFSF9 | -0,595929969 |
| MGAT3 | -0,594883654 |
| PIAS3 | -0,594177868 |
| ZNF768 | -0,593802132 |
| FAM160B2 | -0,593173971 |
| GSDMD | -0,589261686 |
| TRAF3IP2 | -0,58875066 |
| SASH3 | -0,58875021 |

| | |
|---|---|
| CD44 | -0,583147746 |
| VGLL4 | -0,582447537 |
| SPATA20 | -0,579806368 |
| PRRC2B | -0,578237509 |
| C9orf69 | -0,577628695 |
| SFI1 | -0,577361677 |
| TOB2 | -0,57717749 |
| STAMBP | -0,576809778 |
| WDR43 | -0,576331392 |
| ABI3 | -0,574937994 |
| PDLIM7 | -0,574139627 |
| MYO1G | -0,573473161 |
| CCNG2 | -0,573443304 |
| FBXO22 | -0,573227856 |
| MLLT4 | -0,572707931 |
| AMPD2 | -0,572316738 |
| PSIP1 | -0,5720843 |
| AMER1 | -0,571040834 |
| BAX | -0,570133633 |
| DNMT3A | -0,56994628 |
| CYFIP2 | -0,569900314 |
| WHAMM | -0,56717955 |
| ST3GAL2 | -0,566566749 |
| POM121 | -0,565977127 |
| DYNC1I2 | -0,564868371 |
| ALDH4A1 | -0,564235624 |
| ETS1 | -0,563917613 |
| PIP4K2A | -0,561486702 |
| STRN | -0,561138232 |
| CYFIP1 | -0,559160938 |
| ABR | -0,557550326 |
| PHF8 | -0,556353698 |
| TP53BP1 | -0,556034068 |
| ZNF512 | -0,5553717 |
| IKZF3 | -0,553828742 |
| SMG6 | -0,552590613 |
| MAD1L1 | -0,550273467 |
| ZNF281 | -0,550265497 |
| MS4A1 | -0,548669535 |
| CNTROB | -0,546594426 |
| PEX6 | -0,545089579 |
| CEP68 | -0,544819879 |
| OLA1 | -0,544758592 |
| PISD | -0,541151419 |
| DFFA | -0,539923722 |
| CCDC93 | -0,539473313 |
| MIDN | -0,538959972 |

| | |
|---|---|
| ATP1A1 | -0,537513361 |
| BLCAP | -0,535416521 |
| TACC1 | -0,535258664 |
| SORL1 | -0,533280941 |
| POLR1A | -0,531511871 |
| EIF4B | -0,529328045 |
| SLC16A1 | -0,526531135 |
| NCDN | -0,524814024 |
| CUX1 | -0,524706599 |
| ASMTL | -0,521286899 |
| DCAKD | -0,518654141 |
| ALDH1B1 | -0,518278058 |
| PHKA2 | -0,517849912 |
| EIF2AK4 | -0,51635349 |
| AKNA | -0,516311678 |
| TMEM69 | -0,515295622 |
| SMARCC1 | -0,513979763 |
| NFKB1 | -0,512474387 |
| CUL3 | -0,511705133 |
| ELK1 | -0,511700257 |
| TCF12 | -0,511565132 |
| MTHFD1L | -0,511191423 |
| RASGRP3 | -0,510958979 |
| MBNL3 | -0,508589606 |
| BRD4 | -0,508019631 |
| BTBD10 | -0,507733877 |
| ZBTB44 | -0,507541718 |
| DOCK2 | -0,505891124 |
| TRIM41 | -0,505230969 |
| PLEKHO2 | -0,503133963 |
| LUC7L2 | -0,502283869 |
| CCNY | -0,502112784 |
| FOXK1 | -0,502008144 |
| BATF | -0,50184515 |
| POM121C | -0,500009956 |
| DIAPH2 | -0,499434254 |
| FOCAD | -0,498186215 |
| ANP32A | -0,496264915 |
| CYTH1 | -0,496055943 |
| NISCH | -0,494423008 |
| ZFP36L2 | -0,494236391 |
| ANKRD27 | -0,491594956 |
| ANXA6 | -0,491104776 |
| TIPARP | -0,490280564 |
| THAP9-AS1 | -0,48892627 |
| TAF15 | -0,488416185 |
| MGAT5 | -0,487792752 |

| | |
|---|---|
| RBMX2 | -0,487379755 |
| UPF3B | -0,485970979 |
| MED28 | -0,484879411 |
| PFN1 | -0,483277246 |
| CCNK | -0,483119672 |
| DEGS1 | -0,480989362 |
| AP1S1 | -0,479401031 |
| EFHD2 | -0,479097251 |
| SUMO1 | -0,478556253 |
| FAM78A | -0,477309903 |
| ZC3H4 | -0,474631774 |
| GATAD2A | -0,473744103 |
| PFKM | -0,471932693 |
| MOB3A | -0,470341361 |
| OTUD4 | -0,469940109 |
| ATP11A | -0,466776949 |
| YLPM1 | -0,466501232 |
| RNF219 | -0,464745593 |
| TP53 | -0,463832577 |
| GRIPAP1 | -0,463695494 |
| NCBP2 | -0,461654762 |
| PACS1 | -0,460859825 |
| FAM168A | -0,456976466 |
| PLEKHG2 | -0,453231107 |
| CAPN15 | -0,452712108 |
| SH3KBP1 | -0,452181002 |
| STAT5B | -0,4515917 |
| RBM17 | -0,451331553 |
| KDM4B | -0,450834574 |
| C11orf57 | -0,449731123 |
| FAM13B | -0,446363382 |
| HMG20B | -0,446337537 |
| EIF5B | -0,445395308 |
| TSR2 | -0,444706733 |
| SEPT2 | -0,444347087 |
| VASP | -0,443845398 |
| STK11 | -0,440443068 |
| CNOT6 | -0,439731827 |
| RBM8A | -0,439679403 |
| TMEM57 | -0,435756786 |
| MYC | -0,435283398 |
| ST13 | -0,435224521 |
| NCOR2 | -0,43384975 |
| TAF1 | -0,433636477 |
| TTL | -0,431669515 |
| ODF2 | -0,43138823 |
| HTT | -0,431373212 |

| | |
|---|---|
| CAD | -0,43086404 |
| ATP11C | -0,430382021 |
| SARS | -0,430246973 |
| LPXN | -0,429423554 |
| RPS2 | -0,424109959 |
| MEN1 | -0,424065318 |
| CABIN1 | -0,423322582 |
| ERICH1 | -0,423218459 |
| HNRNPH3 | -0,42278067 |
| FAM168B | -0,422415893 |
| SCRIB | -0,422234197 |
| TRIP12 | -0,418801608 |
| RPS6KA4 | -0,418001447 |
| PRPF40A | -0,415835159 |
| CAPNS1 | -0,414449153 |
| REXO1 | -0,413911097 |
| MTA1 | -0,413444447 |
| IL17RA | -0,411467717 |
| GNAI2 | -0,4114011 |
| SZRD1 | -0,409399426 |
| MLLT6 | -0,40725263 |
| PIP4K2B | -0,405701919 |
| ARID3B | -0,401374328 |
| SF1 | -0,40003214 |
| DROSHA | -0,399324426 |
| EML3 | -0,395710716 |
| LRRFIP1 | -0,395517545 |
| OXSR1 | -0,394870216 |
| RFX7 | -0,389690618 |
| HDLBP | -0,38821861 |
| C9orf114 | -0,386902216 |
| SF3A1 | -0,38570875 |
| SIN3A | -0,384756777 |
| MGRN1 | -0,383932879 |
| SMARCE1 | -0,380661221 |
| DENND1C | -0,379071732 |
| MED15 | -0,377156285 |
| SUPT16H | -0,374438783 |
| GTF2F1 | -0,373087569 |
| SRRM1 | -0,364846714 |
| STAT6 | -0,36483538 |
| PPP1R18 | -0,360690614 |
| EIF3D | -0,359288711 |
| AHCYL1 | -0,355521922 |
| MSN | -0,353946259 |
| STK26 | -0,353256328 |
| MEF2C | -0,351732456 |

| | |
|---|---:|
| FLII | -0,351437744 |
| ILF3 | -0,351224364 |
| PSD4 | -0,349349345 |
| PRRC2A | -0,330883512 |
| TLN1 | -0,314104034 |

Up-regulated

| Gene | log2FoldChange |
|---|---:|
| CD53 | 0,305702767 |
| PDXK | 0,348747429 |
| MAP2K1 | 0,359567537 |
| GANAB | 0,36422689 |
| ATL3 | 0,371693872 |
| SDF4 | 0,380553728 |
| PICALM | 0,380858111 |
| CANX | 0,382526448 |
| ATP13A1 | 0,39619729 |
| CASP3 | 0,400326624 |
| HAX1 | 0,401411515 |
| B2M | 0,401536887 |
| LRPAP1 | 0,410047965 |
| ATP11B | 0,410528706 |
| SELT | 0,415061563 |
| TPP1 | 0,415499468 |
| PAK1 | 0,419008153 |
| ARF1 | 0,422001389 |
| ERGIC2 | 0,422204833 |
| KIAA2013 | 0,424666444 |
| HLA-B | 0,424714315 |
| SS18 | 0,430167042 |
| NUCB1 | 0,430484409 |
| ANKRD28 | 0,431425791 |
| SUMF2 | 0,431508842 |
| SEC23B | 0,431761276 |
| MBD1 | 0,434162033 |
| TMEM263 | 0,438943233 |
| ZMYND11 | 0,441652819 |
| CHSY1 | 0,441958425 |
| DNASE2 | 0,442384734 |
| MORF4L2 | 0,443017743 |
| HSH2D | 0,44430423 |
| HAUS6 | 0,447086363 |
| TBC1D1 | 0,447173646 |
| ATP2A3 | 0,450297278 |
| C16orf62 | 0,451161307 |
| CLPTM1 | 0,451367822 |

| | |
|---|---|
| ABI1 | 0,451847153 |
| STT3B | 0,453013464 |
| CD79A | 0,460959932 |
| RPUSD1 | 0,464439841 |
| TBL1XR1 | 0,466729655 |
| KIAA1551 | 0,468561875 |
| YIPF2 | 0,468655347 |
| GOLPH3 | 0,470983101 |
| TXNDC15 | 0,472154935 |
| KLHL5 | 0,479440515 |
| TXNDC12 | 0,481043124 |
| HSPB11 | 0,482567337 |
| ATP2A2 | 0,4826752 |
| OS9 | 0,484704968 |
| LAT2 | 0,488411895 |
| RCBTB2 | 0,489058539 |
| ARF4 | 0,4895235 |
| SLC10A3 | 0,490565387 |
| ENTPD4 | 0,491170284 |
| SFT2D1 | 0,493666024 |
| TMEM214 | 0,495437889 |
| SGSM3 | 0,495781247 |
| CLCC1 | 0,495802798 |
| NARS | 0,496212265 |
| TES | 0,496457985 |
| SEC13 | 0,49726642 |
| CCM2 | 0,49750851 |
| GOLGA3 | 0,498608257 |
| VAPA | 0,498730272 |
| COG3 | 0,503068333 |
| FGFR1OP | 0,503720835 |
| MPDU1 | 0,504311145 |
| USP48 | 0,505593416 |
| AARS | 0,50606509 |
| CIB1 | 0,508601584 |
| ATP6AP1 | 0,51009738 |
| RUFY1 | 0,511045252 |
| FANCA | 0,512102258 |
| HEXA | 0,515461109 |
| AMFR | 0,516280681 |
| GALNT1 | 0,516633615 |
| TM9SF2 | 0,518281889 |
| CCBL2 | 0,522890485 |
| PLOD1 | 0,52321147 |
| TMED4 | 0,523496984 |
| MYL12B | 0,524249218 |
| PARP2 | 0,526155619 |

| | |
|---|---|
| BAK1 | 0,527391155 |
| METTL23 | 0,527561077 |
| MIS12 | 0,527625219 |
| ADPGK | 0,527753562 |
| PREB | 0,529680494 |
| TXNL4A | 0,532095033 |
| AFF4 | 0,534179938 |
| ZNF587 | 0,538805988 |
| COLGALT1 | 0,541237016 |
| PMM1 | 0,541502481 |
| ATP2C1 | 0,541894475 |
| SLC35B3 | 0,541981016 |
| LONRF1 | 0,542561434 |
| RPS6KB2 | 0,543564484 |
| PPT1 | 0,544869043 |
| CHPF2 | 0,546180537 |
| DNAJC1 | 0,54663114 |
| RAB30 | 0,547920436 |
| SMCHD1 | 0,548186491 |
| MUT | 0,548284561 |
| RLF | 0,549186967 |
| IL12RB1 | 0,549801458 |
| GZF1 | 0,551254541 |
| NXPE3 | 0,552934771 |
| GLT8D1 | 0,558141651 |
| SSR2 | 0,559865135 |
| MBD4 | 0,560930846 |
| NAGK | 0,562014282 |
| TMED5 | 0,56641436 |
| ADNP2 | 0,568426278 |
| SLC38A2 | 0,569003394 |
| CHID1 | 0,569284887 |
| CYBA | 0,569699456 |
| MYL12A | 0,570465531 |
| CISD2 | 0,572346377 |
| ZNF652 | 0,574615329 |
| CCDC109B | 0,576186798 |
| SLC38A10 | 0,57632519 |
| EMC7 | 0,577809731 |
| ADGRE5 | 0,57797111 |
| ATP6V1A | 0,578242561 |
| SPAST | 0,580345005 |
| PNPLA8 | 0,580966944 |
| SLC3A2 | 0,581242834 |
| LAMP2 | 0,586299116 |
| MTG2 | 0,590110078 |
| PLOD3 | 0,594103202 |

| | |
|---|---|
| MGAT2 | 0,595736711 |
| SEC61G | 0,59608463 |
| AP1S3 | 0,596701345 |
| C12orf4 | 0,597086503 |
| ALG12 | 0,597719613 |
| PSMG2 | 0,59793313 |
| BMF | 0,598079701 |
| NAPG | 0,599194292 |
| NGLY1 | 0,60071926 |
| NAGA | 0,600777345 |
| EMC3 | 0,601229059 |
| CDKN1B | 0,603386525 |
| UNC13D | 0,603521127 |
| GNE | 0,604396526 |
| CREB3 | 0,606007972 |
| KLHDC10 | 0,607378284 |
| CTSD | 0,607667272 |
| LMBRD1 | 0,608425215 |
| PRDM15 | 0,608694415 |
| SEMA4A | 0,609060774 |
| GNPTG | 0,610052564 |
| ATP6V1D | 0,610101989 |
| SCPEP1 | 0,611679941 |
| ATOX1 | 0,612801499 |
| B4GALT4 | 0,613653929 |
| ING1 | 0,615090541 |
| ZNF331 | 0,616874803 |
| ADK | 0,618596574 |
| NCAPH2 | 0,619299747 |
| RPS19BP1 | 0,620514475 |
| NAA50 | 0,622101596 |
| GBA | 0,622995071 |
| GRK6 | 0,623052255 |
| MICB | 0,623935197 |
| RPRD1A | 0,624277635 |
| SRPRB | 0,624884683 |
| FAM50B | 0,625606943 |
| SLC52A2 | 0,626264262 |
| MELK | 0,627527368 |
| SPNS1 | 0,628450782 |
| ME1 | 0,629049094 |
| DXO | 0,629146803 |
| MFSD12 | 0,63018644 |
| NEMP2 | 0,63170639 |
| MFSD11 | 0,640184284 |
| NIPSNAP3A | 0,642310207 |
| GLA | 0,642465544 |

| | |
|---|---|
| RHOQ | 0,643847796 |
| SRPR | 0,644029346 |
| ATF7IP2 | 0,644639828 |
| PELO | 0,646177597 |
| GMPPA | 0,646221614 |
| SPTY2D1 | 0,646804624 |
| ST7 | 0,648288924 |
| SUB1 | 0,649363621 |
| ANKRD12 | 0,649833507 |
| P2RY10 | 0,649888256 |
| RRNAD1 | 0,651890222 |
| ALG2 | 0,652046515 |
| SEP15 | 0,65284664 |
| CAMKK2 | 0,656762046 |
| SLC35C1 | 0,658874117 |
| RAD9A | 0,660845925 |
| TTC14 | 0,661457186 |
| TMED1 | 0,661613394 |
| CD99L2 | 0,662071681 |
| TMEM39A | 0,662168634 |
| GNS | 0,667399421 |
| STS | 0,667408379 |
| ZFP36 | 0,667990471 |
| ABCB6 | 0,66809479 |
| HDHD2 | 0,669293181 |
| INSIG2 | 0,669987552 |
| NMRAL1 | 0,670854587 |
| FUCA2 | 0,674595814 |
| SLC17A9 | 0,67597793 |
| ITGA4 | 0,67783862 |
| CCDC53 | 0,679963084 |
| CENPN | 0,680935673 |
| ALG8 | 0,685074215 |
| NCKAP1 | 0,685101464 |
| ZNF275 | 0,686051579 |
| RAB24 | 0,690101265 |
| ZNF296 | 0,690622363 |
| UQCC2 | 0,691022051 |
| C10orf32 | 0,691245947 |
| SPCS1 | 0,692176476 |
| YIPF6 | 0,692352204 |
| FAM214A | 0,694351649 |
| ENTPD7 | 0,696437079 |
| ITCH | 0,698311688 |
| OSTC | 0,702850732 |
| SLC10A7 | 0,70285712 |
| ALDH3A2 | 0,705593576 |

| | |
|---|---|
| CPD | 0,70573161 |
| ALG5 | 0,705877401 |
| NANS | 0,708439541 |
| CAMK4 | 0,710264505 |
| ZNF701 | 0,711317209 |
| PLD3 | 0,712651398 |
| LYSMD3 | 0,712751805 |
| TAPT1 | 0,713970752 |
| SEC14L1 | 0,717107682 |
| DPAGT1 | 0,718077185 |
| BTLA | 0,718671294 |
| ASF1A | 0,719082637 |
| SMPD1 | 0,721703377 |
| KPTN | 0,722490595 |
| RFC3 | 0,722937515 |
| CTBS | 0,727097392 |
| FAM63B | 0,728024467 |
| ARMCX3 | 0,728501166 |
| CYB561D2 | 0,728520964 |
| EDEM2 | 0,739157735 |
| CALU | 0,741372569 |
| NDUFV2 | 0,742536036 |
| SDCBP | 0,742667836 |
| VEGFA | 0,742911482 |
| CARD11 | 0,746318802 |
| ASPHD2 | 0,747463309 |
| FNDC3A | 0,747490898 |
| NOMO1 | 0,749878758 |
| FAM8A1 | 0,751918099 |
| KIR3DX1 | 0,752485678 |
| TAP2 | 0,763991597 |
| C16orf54 | 0,765683919 |
| RHBDD1 | 0,769097798 |
| JTB | 0,770349439 |
| EIF2B3 | 0,774563925 |
| LEPROT | 0,776929891 |
| NEMP1 | 0,777287177 |
| PSENEN | 0,777577914 |
| FAM174A | 0,778112032 |
| CDK2AP2 | 0,780692994 |
| PSAT1 | 0,781027484 |
| STAMBPL1 | 0,78327099 |
| ZDHHC4 | 0,783737049 |
| SPCS3 | 0,783738144 |
| COX7A2 | 0,785776092 |
| SIL1 | 0,78599946 |
| RAB31 | 0,786958904 |

| | |
|---|---|
| HLA-DPA1 | 0,787036693 |
| CSGALNACT2 | 0,787271931 |
| CMC2 | 0,788752745 |
| TMEM184B | 0,791951519 |
| WDR45 | 0,792545101 |
| NEU1 | 0,792650784 |
| RAPGEF2 | 0,796337313 |
| AGPAT2 | 0,797331641 |
| PPAPDC1B | 0,797745196 |
| KNTC1 | 0,798610658 |
| C18orf8 | 0,799192937 |
| DALRD3 | 0,799891999 |
| LINC00467 | 0,807287807 |
| CDC25B | 0,807422525 |
| TP53I13 | 0,809249579 |
| CD320 | 0,815759578 |
| SEC24A | 0,818464376 |
| CITED2 | 0,819167674 |
| DOK3 | 0,819347875 |
| TMEM208 | 0,819624846 |
| SEMA4B | 0,820426984 |
| SEL1L | 0,822123631 |
| LPIN2 | 0,823087891 |
| ABCA7 | 0,82661397 |
| ACP2 | 0,826697364 |
| CCDC50 | 0,827843463 |
| CIITA | 0,829232303 |
| EIF2AK3 | 0,831292142 |
| FAM117A | 0,833809421 |
| PPP3CC | 0,839960614 |
| EPB41 | 0,840359976 |
| JOSD2 | 0,842146519 |
| NRIP1 | 0,842471523 |
| PRKXP1 | 0,846032382 |
| ENO2 | 0,846056586 |
| PLA2G16 | 0,847561918 |
| ZNF486 | 0,847583255 |
| PRDX1 | 0,848145153 |
| DNASE1L1 | 0,85014169 |
| POLQ | 0,850230848 |
| ZNF841 | 0,85230424 |
| ZNF397 | 0,852979944 |
| E2F2 | 0,858148365 |
| EMP3 | 0,863350804 |
| BCORL1 | 0,864789301 |
| KIAA0040 | 0,865717332 |
| CEP128 | 0,866584966 |

| | |
|---|---|
| JAK2 | 0,872699385 |
| WFS1 | 0,873525472 |
| RNF149 | 0,879325997 |
| RAD51AP1 | 0,880397818 |
| IER3IP1 | 0,88148907 |
| TAF4B | 0,88347096 |
| CDC42BPB | 0,885679304 |
| CDK14 | 0,888188511 |
| SKA1 | 0,888948677 |
| SLC41A2 | 0,891573569 |
| ATP6V0B | 0,894632554 |
| HLA-DPB1 | 0,898266629 |
| TMEM70 | 0,899514712 |
| ZNF320 | 0,899641929 |
| HSPA13 | 0,899657955 |
| SLC35B1 | 0,900363502 |
| FICD | 0,908527954 |
| EAF2 | 0,910434845 |
| SMCO4 | 0,911607334 |
| MRPL14 | 0,912995166 |
| LSR | 0,92147062 |
| TCAF2 | 0,928330158 |
| STAP1 | 0,942148593 |
| QPCTL | 0,946630135 |
| PLP2 | 0,947587257 |
| TCF19 | 0,953057183 |
| P2RX4 | 0,95447122 |
| FBXO16 | 0,95826053 |
| TMEM106A | 0,959535791 |
| C5orf30 | 0,968298049 |
| HIPK2 | 0,96961605 |
| PRAF2 | 0,971930669 |
| CLPTM1L | 0,979912259 |
| CBFA2T3 | 0,979923205 |
| HLA-DMA | 0,983894629 |
| DEF8 | 0,984950704 |
| B3GNT9 | 0,992955626 |
| GFI1 | 0,995767572 |
| SRGN | 0,996446522 |
| KCNA3 | 0,997084732 |
| HLA-DRA | 0,998476882 |
| ZBTB20 | 0,999480079 |
| GMNN | 0,999656648 |
| NEK3 | 1,34689E+12 |
| ERC1 | 1,39412E+12 |
| MUC20 | 2,81605E+12 |
| SLC6A9 | 1,04566E+13 |

| | |
|---|---|
| TPST2 | 1,08914E+13 |
| HIST1H2BK | 1,18221E+13 |
| KIAA1549L | 1,25846E+13 |
| FAM46C | 1,30318E+13 |
| EGR1 | 1,40463E+13 |
| GALC | 1,47652E+13 |
| SHROOM1 | 1,49463E+13 |
| TMEM45A | 1,50977E+13 |
| CRY1 | 1,57273E+13 |
| ENPP5 | 1,58398E+13 |
| CD72 | 1,66071E+13 |
| CPM | 1,71156E+13 |
| MIXL1 | 1,79214E+13 |
| MYH15 | 2,43173E+13 |
| TEX15 | 3,19762E+13 |
| GATA4 | 3,20173E+13 |
| LOC101928767 | 3,3115E+13 |
| ARHGEF37 | 3,36565E+13 |
| CLIC5 | 3,38792E+13 |
| SERPINA9 | 3,97592E+13 |
| SEL1L3 | 4,07977E+13 |
| T | 4,10358E+13 |
| FAM174B | 4,53935E+13 |
| DCC | 5,04839E+13 |
| ZNF354C | 5,23442E+13 |
| BMP8B | 5,43232E+13 |
| PBX4 | 5,47194E+13 |
| CFAP43 | 5,76575E+13 |
| ADAD2 | 5,8713E+13 |
| LOC646762 | 5,9904E+13 |
| GLYATL2 | 6,02308E+13 |
| NLRP11 | 6,5684E+13 |
| LDLRAD2 | 6,57565E+13 |
| PHYHD1 | 7,60877E+13 |
| XIST | 9,32236E+13 |
| SHCBP1 | 1,00119E+14 |
| CAPG | 1,00136E+14 |
| DNASE1 | 1,00178E+14 |
| SDHAP1 | 1,00837E+14 |
| MANEA | 1,01492E+14 |
| HYI | 1,0218E+14 |
| VNN2 | 1,02352E+14 |
| CEACAM21 | 1,02385E+14 |
| ATF5 | 1,02528E+14 |
| RUFY3 | 1,02552E+14 |
| INO80C | 1,02735E+14 |
| CISD3 | 1,02808E+14 |

| | |
|---|---|
| NCF4 | 1,03057E+14 |
| GGH | 1,03198E+14 |
| BIN2 | 1,03235E+14 |
| NUCB2 | 1,03374E+14 |
| ANKRD37 | 1,03654E+14 |
| CCR10 | 1,03845E+14 |
| STAP2 | 1,04631E+14 |
| IGFLR1 | 1,05247E+14 |
| GPR155 | 1,05393E+14 |
| C7orf13 | 1,06259E+14 |
| ZNF844 | 1,06977E+14 |
| HLA-DRB1 | 1,08526E+14 |
| CCPG1 | 1,09293E+14 |
| ABHD6 | 1,09322E+14 |
| ZNF714 | 1,09335E+14 |
| MSL3P1 | 1,09638E+14 |
| CYB561A3 | 1,09949E+14 |
| CYP1B1 | 1,10099E+14 |
| SRXN1 | 1,12029E+14 |
| ZFP82 | 1,1229E+14 |
| ABCD2 | 1,13015E+14 |
| TYMP | 1,13538E+14 |
| DNLZ | 1,13771E+14 |
| ANK2 | 1,13902E+14 |
| ITM2C | 1,14492E+14 |
| ARRDC3 | 1,14693E+14 |
| ASS1 | 1,15483E+14 |
| ANXA1 | 1,15874E+14 |
| SLC16A3 | 1,16007E+14 |
| KIAA0513 | 1,16176E+14 |
| PLIN2 | 1,16427E+14 |
| B3GALT4 | 1,16428E+14 |
| GPR55 | 1,17939E+14 |
| CYB561 | 1,18203E+14 |
| RNF157 | 1,18251E+14 |
| ASPH | 1,18885E+14 |
| LRRC8B | 1,19166E+14 |
| AGMAT | 1,19691E+14 |
| WNT10A | 1,19751E+14 |
| ZNF681 | 1,19806E+14 |
| TNFRSF17 | 1,20095E+14 |
| APOL1 | 1,20766E+14 |
| TWSG1 | 1,2115E+14 |
| CLEC2D | 1,2121E+14 |
| TM6SF1 | 1,22339E+14 |
| C17orf96 | 1,2252E+14 |
| CD68 | 1,23602E+14 |

| | |
|---|---|
| ZNF229 | 1,23705E+14 |
| LINC00996 | 1,23822E+14 |
| MICAL2 | 1,24012E+14 |
| RN7SK | 1,24196E+14 |
| FOXO3B | 1,24532E+14 |
| SNX18 | 1,25123E+14 |
| CLDN14 | 1,26938E+14 |
| LIME1 | 1,27743E+14 |
| LRFN4 | 1,28251E+14 |
| HLA-DOB | 1,29304E+14 |
| LINC00152 | 1,29387E+14 |
| LINC00324 | 1,29844E+14 |
| GNB4 | 1,29864E+14 |
| LTBP3 | 1,30396E+14 |
| MIR22HG | 1,31077E+14 |
| P2RX1 | 1,31495E+14 |
| RNASE6 | 1,31825E+14 |
| UNC13B | 1,32313E+14 |
| SLC48A1 | 1,3311E+14 |
| FNDC3B | 1,33928E+14 |
| BTD | 1,34094E+14 |
| NADK2 | 1,34265E+14 |
| PINLYP | 1,34913E+14 |
| GALNT3 | 1,35146E+14 |
| TCN2 | 1,36666E+14 |
| SLFN11 | 1,37528E+14 |
| MTSS1 | 1,38006E+14 |
| RWDD2A | 1,38738E+14 |
| VASH2 | 1,39006E+14 |
| ARHGEF35 | 1,39757E+14 |
| ZYG11A | 1,40058E+14 |
| NPDC1 | 1,40765E+14 |
| RNF122 | 1,41624E+14 |
| SIK1 | 1,42658E+14 |
| DDX12P | 1,43074E+14 |
| TUBB2B | 1,43424E+14 |
| HMSD | 1,43984E+14 |
| HLA-DQA1 | 1,4485E+14 |
| ARHGEF5 | 1,45046E+14 |
| SPATS2L | 1,45118E+14 |
| DHRS13 | 1,45294E+14 |
| MYO3B | 1,45321E+14 |
| SBF2-AS1 | 1,46806E+14 |
| ZNF665 | 1,47056E+14 |
| SLC26A11 | 1,48095E+14 |
| KCNK1 | 1,48763E+14 |
| PRICKLE1 | 1,49302E+14 |

| | |
|---|---|
| SERPINB10 | 1,49539E+14 |
| TLR7 | 1,49675E+14 |
| NOL3 | 1,50158E+14 |
| GPNMB | 1,5028E+14 |
| LINC01480 | 1,50576E+14 |
| MLC1 | 1,50631E+14 |
| SAMSN1 | 1,51617E+14 |
| KL | 1,51837E+14 |
| FKBP11 | 1,51957E+14 |
| CELSR2 | 1,53055E+14 |
| TFR2 | 1,5434E+14 |
| KIAA0226L | 1,54806E+14 |
| HSPA5 | 1,54847E+14 |
| F12 | 1,55407E+14 |
| HSBP1L1 | 1,55936E+14 |
| ZNF571-AS1 | 1,56902E+14 |
| DEPDC7 | 1,576E+14 |
| SLC27A2 | 1,58245E+14 |
| LBX2-AS1 | 1,58938E+14 |
| KCTD12 | 1,59026E+14 |
| IL12RB2 | 1,61501E+14 |
| TMEM44-AS1 | 1,61609E+14 |
| AHNAK | 1,61908E+14 |
| HIST1H4H | 1,6205E+14 |
| ABCA3 | 1,634E+14 |
| SLC30A4 | 1,64392E+14 |
| DNAH17 | 1,65765E+14 |
| MIR5195 | 1,67279E+14 |
| ESR2 | 1,69414E+14 |
| DCHS1 | 1,69535E+14 |
| PVRIG | 1,69885E+14 |
| SOWAHD | 1,71097E+14 |
| LOXL2 | 1,71977E+14 |
| ARHGEF34P | 1,72475E+14 |
| GPR19 | 1,73319E+14 |
| CKAP4 | 1,73326E+14 |
| SPTBN4 | 1,79171E+14 |
| ZBTB8A | 1,79582E+14 |
| NOTCH2 | 1,81185E+14 |
| LOC613266 | 1,81934E+14 |
| KIAA1217 | 1,82404E+14 |
| FCRL5 | 1,83042E+14 |
| KCNK6 | 1,83251E+14 |
| COL4A4 | 1,83694E+14 |
| LOC729603 | 1,84583E+14 |
| ZFYVE9 | 1,86495E+14 |
| FAAH | 1,86571E+14 |

| | |
|---|---|
| ERN1 | 1,86628E+14 |
| MIR4539 | 1,86677E+14 |
| C1orf162 | 1,86884E+14 |
| BTBD19 | 1,86906E+14 |
| PTPRN2 | 1,87093E+14 |
| MPZ | 1,87832E+14 |
| AEBP1 | 1,88255E+14 |
| GAB1 | 1,88263E+14 |
| NEFH | 1,88264E+14 |
| NFIX | 1,90176E+14 |
| INPP5F | 1,90904E+14 |
| EPS8L1 | 1,91165E+14 |
| HCAR3 | 1,94438E+14 |
| DIP2C | 1,97247E+14 |
| RGCC | 1,97658E+14 |
| COL4A3 | 2,01834E+14 |
| CBARP | 2,04982E+14 |
| CHST6 | 2,06348E+14 |
| CCR1 | 2,06451E+14 |
| NOD2 | 2,08574E+14 |
| IRF6 | 2,0903E+14 |
| LIPG | 2,11005E+14 |
| LRP12 | 2,11457E+14 |
| LINC00539 | 2,1209E+14 |
| BCAR3 | 2,12451E+14 |
| TMEM65 | 2,13467E+14 |
| CD9 | 2,14218E+14 |
| ZNF793-AS1 | 2,15072E+14 |
| EVI2A | 2,15727E+14 |
| MUC4 | 2,15826E+14 |
| ARHGAP42 | 2,18118E+14 |
| PDGFA | 2,19047E+14 |
| BCL2L10 | 2,1966E+14 |
| LINC00528 | 2,19945E+14 |
| CLEC2B | 2,20226E+14 |
| MT2A | 2,20264E+14 |
| CFAP54 | 2,23559E+14 |
| PDE9A | 2,25257E+14 |
| MAFF | 2,26499E+14 |
| HSPA7 | 2,269E+14 |
| BAIAP3 | 2,28385E+14 |
| SSTR3 | 2,33535E+14 |
| CCDC144B | 2,35323E+14 |
| TPTE2 | 2,44311E+14 |
| PRKCZ | 2,46299E+14 |
| LGALS3BP | 2,46327E+14 |
| ZNF711 | 2,46894E+14 |

| | |
|---|---|
| LYPD6B | 2,47619E+14 |
| PC | 2,48864E+14 |
| GLB1L2 | 2,49711E+14 |
| RIMBP2 | 2,50329E+14 |
| RGAG1 | 2,51029E+14 |
| PDE6G | 2,52863E+14 |
| CEBPA | 2,54359E+14 |
| ADAP2 | 2,5552E+14 |
| IL32 | 2,56503E+14 |
| MIR4538 | 2,56509E+14 |
| POU4F1 | 2,57061E+14 |
| TNFRSF18 | 2,59448E+14 |
| LAD1 | 2,59452E+14 |
| C17orf107 | 2,62084E+14 |
| ZBP1 | 2,65359E+14 |
| HRASLS2 | 2,67372E+14 |
| SERPINB2 | 2,70461E+14 |
| PTPRS | 2,70502E+14 |
| PLEKHA7 | 2,70597E+14 |
| MAP1LC3A | 2,74522E+14 |
| IRS2 | 2,7581E+14 |
| ESPNL | 2,78251E+14 |
| SPTA1 | 2,80273E+14 |
| RAB36 | 2,83668E+14 |
| ST14 | 2,83961E+14 |
| MYO1D | 2,84347E+14 |
| ACRBP | 2,84431E+14 |
| CTBP2 | 2,89189E+14 |
| LMCD1 | 2,91448E+14 |
| ST3GAL6 | 2,9308E+14 |
| RARRES2 | 2,93242E+14 |
| NDFIP1 | 2,93563E+14 |
| SERPINF1 | 2,95109E+14 |
| CKB | 2,97377E+14 |
| FAM109B | 2,97591E+14 |
| ITGAX | 2,97668E+14 |
| C11orf63 | 2,97846E+14 |
| JCHAIN | 2,98778E+14 |
| COTL1 | 2,99097E+14 |
| TIMD4 | 3,01162E+14 |
| TCL6 | 3,01927E+14 |
| CD180 | 3,02032E+14 |
| FAM171A1 | 3,02134E+14 |
| BTN1A1 | 3,02442E+14 |
| FHOD3 | 3,02743E+14 |
| MZB1 | 3,04149E+14 |
| NSUN7 | 3,06086E+14 |

| | |
|---|---|
| TESC | 3,09034E+14 |
| PECAM1 | 3,10503E+14 |
| INPP5J | 3,10563E+14 |
| ESRP2 | 3,10835E+14 |
| ZNF208 | 3,12503E+14 |
| PCBP3 | 3,14235E+14 |
| RNF5P1 | 3,16595E+14 |
| LDOC1L | 3,16666E+14 |
| IFI27 | 3,18555E+14 |
| CASP1 | 3,20406E+14 |
| LNX1 | 3,22206E+14 |
| ASMT | 3,23345E+14 |
| CUEDC1 | 3,23592E+14 |
| RNASE4 | 3,24453E+14 |
| PYHIN1 | 3,28529E+14 |
| LAMA5 | 3,30662E+14 |
| PROSER2 | 3,3112E+14 |
| ARHGEF10 | 3,4008E+14 |
| ZNF300 | 3,40976E+14 |
| NEURL1 | 3,41792E+14 |
| COL6A1 | 3,41969E+14 |
| DLGAP2 | 3,43122E+14 |
| ADAMTS17 | 3,43491E+14 |
| DNAH17-AS1 | 3,45317E+14 |
| TRPS1 | 3,45647E+14 |
| EVC | 3,4575E+14 |
| TRIM47 | 3,49554E+14 |
| MCOLN3 | 3,52079E+14 |
| PRR18 | 3,5403E+14 |
| FAM129C | 3,5489E+14 |
| RHBDF1 | 3,58876E+14 |
| GAS6 | 3,59898E+14 |
| LINC00242 | 3,61483E+14 |
| LOC441666 | 3,62484E+14 |
| KCNK5 | 3,66615E+14 |
| SYT12 | 3,67259E+14 |
| GABRR2 | 3,68702E+14 |
| DERL3 | 3,68918E+14 |
| GJB2 | 3,69788E+14 |
| CTGLF12P | 3,71848E+14 |
| ARHGEF10L | 3,72574E+14 |
| USP44 | 3,72745E+14 |
| RASGEF1A | 3,73061E+14 |
| MARC2 | 3,75196E+14 |
| PCSK6 | 3,83591E+14 |
| SIT1 | 3,90684E+14 |
| ITGA6 | 3,9085E+14 |

| | |
|---|---|
| IGF2BP1 | 3,93778E+14 |
| PTPN21 | 3,95753E+14 |
| CDHR1 | 3,97514E+14 |
| CRNDE | 3,99158E+14 |
| HORMAD2-AS1 | 4,05066E+14 |
| ADAMTSL2 | 4,05657E+14 |
| SCGB3A1 | 4,09664E+14 |
| MTCL1 | 4,11213E+14 |
| SYDE2 | 4,12212E+14 |
| DENND2C | 4,20681E+14 |
| ANKRD36BP2 | 4,21624E+14 |
| PDCD1 | 4,31577E+14 |
| TEAD1 | 4,41137E+14 |
| FMNL2 | 4,4727E+14 |
| HMX2 | 4,52718E+14 |
| A2M | 4,53166E+14 |
| ZNF492 | 4,54519E+14 |
| WFDC2 | 4,56199E+14 |
| PXDN | 4,60727E+14 |
| CCNI2 | 4,61603E+14 |
| KLB | 4,65206E+14 |
| XIRP1 | 4,65642E+14 |
| GPR141 | 4,66867E+14 |
| ADTRP | 4,66933E+14 |
| LHFPL1 | 4,68921E+14 |
| ZNF542P | 4,69438E+14 |
| LINC00540 | 4,79017E+14 |
| GSTM3 | 4,81769E+14 |
| SYBU | 4,83002E+14 |
| CHST4 | 4,91477E+14 |
| ITM2A | 4,92279E+14 |
| SLC6A12 | 4,94743E+14 |
| ITGB5 | 5,02846E+14 |
| SOX18 | 5,05506E+14 |
| PIEZO2 | 5,06019E+14 |
| EPB41L4A | 5,07744E+14 |
| ARSD | 5,27059E+14 |
| PPP1R27 | 5,29453E+14 |
| NUAK2 | 5,2986E+14 |
| XCL1 | 5,30707E+14 |
| CLEC4C | 5,34988E+14 |
| ACSBG1 | 5,3946E+14 |
| ADGRE1 | 5,4039E+14 |
| EDNRA | 5,40568E+14 |
| ELFN1-AS1 | 5,50508E+14 |
| AMPD1 | 5,50908E+14 |
| MARK1 | 5,54398E+14 |

| | |
|---|---|
| SCT | 5,56716E+14 |
| TSPAN1 | 5,60955E+14 |
| ACE | 5,61052E+14 |
| ANKK1 | 5,65482E+14 |
| LRRN2 | 5,67854E+14 |
| EGFR | 5,70722E+14 |
| XKRX | 5,82863E+14 |
| KGFLP1 | 5,82955E+14 |
| LONRF3 | 5,85823E+14 |
| TMEM52B | 5,88831E+14 |
| NUP62CL | 6,05523E+14 |
| LRP3 | 6,07769E+14 |
| THNSL2 | 6,0808E+14 |
| IFNG | 6,19331E+14 |
| COL4A2 | 6,21845E+14 |
| SAMD13 | 6,22488E+14 |
| TSPAN7 | 6,25838E+14 |
| LIX1 | 6,2885E+14 |
| KDELR3 | 6,33684E+14 |
| SLC8A1 | 6,36907E+14 |
| MAP1B | 6,43971E+14 |
| SLC32A1 | 6,52996E+14 |
| GIMAP4 | 6,57335E+14 |
| EDNRB | 6,70032E+14 |
| HHEX | 6,7595E+14 |
| CCR2 | 6,90284E+14 |
| APP | 7,06132E+14 |
| PRSS21 | 7,29639E+14 |
| TLE1 | 7,30108E+14 |
| TCL1B | 7,46692E+14 |

Supplementary Table 7. Differentially expressed genes (up- and downregulated) in EP compared to CdLS cell lines.

Down-regulated

| Gene | log2FoldChange |
|---|---|
| CHIA | -6,7219E+14 |
| ITGA11 | -6,29338E+14 |
| C1orf21 | -5,83974E+14 |
| PAX8-AS1 | -5,21556E+14 |
| PLCB1 | -5,18647E+14 |
| SIGLEC15 | -5,08284E+14 |
| SCHIP1 | -5,06417E+14 |
| CA4 | -4,97667E+14 |
| PPP2R2C | -4,70739E+14 |
| CRTAM | -4,68034E+14 |
| CTTNBP2 | -4,46948E+14 |
| RIN2 | -4,40295E+14 |
| MIR548D1 | -4,35559E+14 |
| CORO2A | -4,35007E+14 |
| CNTNAP3 | -4,22921E+14 |
| JAKMIP2 | -4,20219E+14 |
| FOXG1 | -4,1198E+14 |
| NPY | -4,11822E+14 |
| CBR3 | -4,11084E+14 |
| ATP10A | -4,09979E+14 |
| ZNF462 | -4,01483E+14 |
| DIP2A-IT1 | -3,99539E+14 |
| WWTR1-AS1 | -3,84611E+14 |
| SNORD33 | -3,81519E+14 |
| CALML6 | -3,77748E+14 |
| USP32P2 | -3,57384E+14 |
| LINC00977 | -3,55836E+14 |
| PRKCQ | -3,5335E+14 |
| PKP2 | -3,44477E+14 |
| TPM2 | -3,35093E+14 |
| SNORD47 | -3,26738E+14 |
| APP | -3,20254E+14 |
| TOX | -3,11856E+14 |
| PDE3B | -3,03428E+14 |
| SNORA65 | -2,97458E+14 |
| GRAP2 | -2,89353E+14 |
| RHPN1 | -2,81952E+14 |
| PEX5L | -2,79186E+14 |
| SNORD42A | -2,7587E+14 |
| C17orf97 | -2,66034E+14 |
| DAPK2 | -2,52685E+14 |
| WWC1 | -2,48382E+14 |

| | |
|---|---|
| AXIN2 | -2,43853E+14 |
| LOC101929574 | -2,42077E+14 |
| PAPSS2 | -2,40193E+14 |
| JAKMIP1 | -2,39781E+14 |
| GABRB2 | -2,39198E+14 |
| FAM89A | -2,37491E+14 |
| SPNS3 | -2,37395E+14 |
| TMEM178B | -2,37031E+14 |
| HS3ST1 | -2,32481E+14 |
| ADRB1 | -2,26531E+14 |
| FAM110B | -2,22008E+14 |
| CNKSR2 | -2,20927E+14 |
| MCC | -2,19875E+14 |
| LOC729737 | -2,09445E+14 |
| DLSTP1 | -2,04065E+14 |
| JAM3 | -2,02552E+14 |
| DHRS3 | -1,98917E+14 |
| ARHGEF17 | -1,97952E+14 |
| SNORD45C | -1,97707E+14 |
| STXBP1 | -1,87536E+14 |
| DOCK4 | -1,80635E+14 |
| ITGB3 | -1,79781E+14 |
| HEY1 | -1,7908E+14 |
| SLC23A3 | -1,78908E+14 |
| HPSE | -1,7004E+14 |
| CCDC74A | -1,6139E+14 |
| ERP27 | -1,59873E+14 |
| SYNJ2 | -1,59841E+14 |
| KLF5 | -1,54593E+14 |
| ZFP92 | -1,54378E+14 |
| WDR17 | -1,54102E+14 |
| EDARADD | -1,50885E+14 |
| PTGER4 | -1,49703E+14 |
| TP53BP2 | -1,40328E+14 |
| LOC730183 | -1,36988E+14 |
| SATB1 | -1,32142E+14 |
| AMZ1 | -1,31262E+14 |
| FAM222A | -1,31225E+14 |
| CAPN2 | -1,30142E+14 |
| ACY1 | -1,28331E+14 |
| SNORD5 | -1,26404E+14 |
| FOXD2-AS1 | -1,20733E+14 |
| APOBR | -1,20586E+14 |
| ZMIZ1 | -1,15967E+14 |
| MTHFS | -1,07525E+14 |
| EIF1AY | -1,06589E+14 |
| CDCA7 | -1,02483E+14 |

| | |
|---|---|
| ACCS | -1,01567E+14 |
| TMIGD2 | -6,1429E+13 |
| S100A9 | -5,25753E+13 |
| NCCRP1 | -4,68797E+13 |
| LOC339874 | -4,10344E+13 |
| MMEL1 | -4,01215E+13 |
| SNORD28 | -2,80383E+13 |
| MIR3191 | -1,91922E+13 |
| MIR8072 | -1,50849E+13 |
| GIMAP6 | -1,4734E+13 |
| GBGT1 | -0,991629029 |
| S1PR1 | -0,956967139 |
| CRYBB2P1 | -0,950009039 |
| PIK3C2B | -0,872317706 |
| TMTC4 | -0,871211323 |
| GTF2H2B | -0,842139439 |
| KLF12 | -0,834163393 |
| NAV2 | -0,833767628 |
| MACROD1 | -0,799665593 |
| SLC39A10 | -0,748598408 |
| RGS16 | -0,727575882 |
| E2F8 | -0,711600512 |
| SAMD4A | -0,710303595 |
| ROCK2 | -0,708501016 |
| ELOVL6 | -0,702595978 |
| ENTPD1-AS1 | -0,6790245 |
| RAI1 | -0,673841151 |
| NFATC2 | -0,663377388 |
| SLC35G1 | -0,658210214 |
| FAM72D | -0,658181103 |
| MCM6 | -0,656319861 |
| SPAG16 | -0,649405197 |
| MARCKS | -0,633470248 |
| UHRF1 | -0,628238831 |
| SLC4A7 | -0,621013878 |
| BARD1 | -0,619758636 |
| LY75 | -0,61668858 |
| CEP78 | -0,612746287 |
| ARL5A | -0,602596871 |
| TAF1B | -0,600528708 |
| DUSP7 | -0,595429284 |
| CENPL | -0,588905817 |
| E2F1 | -0,585308241 |
| MBLAC2 | -0,57927944 |
| TMPO | -0,570727056 |
| BLM | -0,570326118 |
| CORO1A | -0,568704098 |

| | |
|---|---|
| LRRC20 | -0,563134293 |
| MCM7 | -0,5609095 |
| SPTLC2 | -0,560172331 |
| RIF1 | -0,556013301 |
| POLR3G | -0,554767366 |
| CPOX | -0,553435415 |
| C4orf46 | -0,552731706 |
| LRRC58 | -0,549113019 |
| PLD6 | -0,547216895 |
| SLC20A2 | -0,546805297 |
| ARL13B | -0,545895779 |
| ENTPD5 | -0,545527125 |
| POLA1 | -0,543710035 |
| ANP32E | -0,532397138 |
| SMC4 | -0,523458889 |
| UBE2D1 | -0,516653811 |
| MSH2 | -0,510476384 |
| OSBPL8 | -0,505319755 |
| ECT2 | -0,504786457 |
| CCNF | -0,504493682 |
| CD3EAP | -0,503109428 |
| ARHGAP11A | -0,500944099 |
| SNRNP48 | -0,499649339 |
| MZT1 | -0,496486935 |
| CDCA4 | -0,493990868 |
| MTHFD1L | -0,490389793 |
| IPMK | -0,486844057 |
| ITPK1 | -0,484379833 |
| PTMA | -0,481538761 |
| SRSF1 | -0,481522789 |
| PRKAA1 | -0,471070577 |
| GINS4 | -0,46843495 |
| XPO4 | -0,466670282 |
| SCAI | -0,465920781 |
| NUCKS1 | -0,460591192 |
| CHD7 | -0,460270597 |
| PFAS | -0,456891286 |
| DCTPP1 | -0,454998493 |
| GPR180 | -0,451341394 |
| CEP135 | -0,450718636 |
| PMS1 | -0,450418728 |
| TRIM33 | -0,450080042 |
| ZNF100 | -0,448303926 |
| DPY19L4 | -0,445386016 |
| SUZ12 | -0,443781246 |
| DHX33 | -0,441760773 |
| FAM122B | -0,439882387 |

| | |
|---|---|
| GMEB1 | -0,438002706 |
| AGPS | -0,437312941 |
| SFMBT1 | -0,43662119 |
| MFNG | -0,434960724 |
| MTFMT | -0,427170515 |
| ACOT7 | -0,425746574 |
| HNRNPD | -0,425585315 |
| PARPBP | -0,425264721 |
| TRUB1 | -0,424703446 |
| GNPNAT1 | -0,424577368 |
| FAM78A | -0,424283149 |
| RANBP1 | -0,424161654 |
| MSH6 | -0,423691097 |
| FBXO45 | -0,421101264 |
| RCL1 | -0,419838133 |
| RFC2 | -0,419085923 |
| LIG3 | -0,417112216 |
| RPGR | -0,41491393 |
| KNOP1 | -0,414878998 |
| TMEM110 | -0,414151965 |
| ZDHHC17 | -0,413916619 |
| TWISTNB | -0,413555286 |
| ALG10B | -0,411350349 |
| BAG4 | -0,411014278 |
| TMEM201 | -0,409448482 |
| LIG1 | -0,408128099 |
| SRSF2 | -0,408048949 |
| GMFB | -0,406728799 |
| LMNB2 | -0,406391452 |
| GABPA | -0,405722485 |
| RRS1 | -0,405327204 |
| TMEM170A | -0,403714075 |
| YTHDC2 | -0,401268091 |
| PABPN1 | -0,397773027 |
| SCOC | -0,393840916 |
| ZNF619 | -0,393108482 |
| NAA25 | -0,3929099 |
| TP53 | -0,391801778 |
| ERI1 | -0,388812001 |
| HDAC2 | -0,386707789 |
| AEBP2 | -0,385985972 |
| PIGW | -0,383324505 |
| TTLL12 | -0,382083593 |
| SLC16A1 | -0,380917833 |
| HNRNPA3 | -0,380233192 |
| LINC00265 | -0,376345026 |
| ARID3A | -0,375638943 |

| | |
|---|---|
| NCL | -0,374772578 |
| MBNL1 | -0,373213099 |
| NOP9 | -0,37301745 |
| PTER | -0,372253594 |
| CENPB | -0,371191791 |
| IPO7 | -0,370812559 |
| TIMM23B | -0,367833653 |
| ESF1 | -0,366848074 |
| GEMIN6 | -0,365835229 |
| SLMO2 | -0,364790843 |
| FUS | -0,361208701 |
| UCK2 | -0,360257677 |
| HNRNPA2B1 | -0,359611521 |
| CASP2 | -0,35937506 |
| CELF1 | -0,357320017 |
| GOLT1B | -0,356810915 |
| TRMT5 | -0,356801385 |
| HDGF | -0,356477463 |
| SORD | -0,355657012 |
| SAP30 | -0,353838663 |
| LYPLA1 | -0,353108729 |
| YWHAQ | -0,351086167 |
| PPP1CB | -0,349338018 |
| SMNDC1 | -0,34777243 |
| TUBA1B | -0,347440496 |
| B4GALT2 | -0,347412487 |
| IKZF3 | -0,347072722 |
| LIMS1 | -0,346543956 |
| PCK2 | -0,346401843 |
| KPNA3 | -0,346393215 |
| MRTO4 | -0,345624831 |
| MOSPD1 | -0,345550025 |
| KLHL42 | -0,343325411 |
| FANCL | -0,343299808 |
| HN1L | -0,343159327 |
| CABLES2 | -0,342926027 |
| PAPD5 | -0,341784389 |
| SHMT2 | -0,340914844 |
| RAD18 | -0,339883777 |
| DPP8 | -0,339433326 |
| PURB | -0,337797471 |
| SREK1IP1 | -0,337710272 |
| TSEN15 | -0,337398792 |
| PGGT1B | -0,337196583 |
| GNA11 | -0,336801964 |
| NIPA2 | -0,33676931 |
| TRA2B | -0,335313075 |

| | |
|---|---|
| C12orf65 | -0,331914499 |
| BAZ1A | -0,328261361 |
| TRA2A | -0,328069719 |
| PROSER1 | -0,326103484 |
| UNG | -0,324203963 |
| RBM14 | -0,323540928 |
| PKNOX1 | -0,323074623 |
| SLC35A3 | -0,322590142 |
| NUDT21 | -0,319794458 |
| ZNF326 | -0,319059419 |
| MAZ | -0,318449726 |
| SET | -0,31838869 |
| KPNB1 | -0,318180142 |
| GATAD2A | -0,317679043 |
| HNRNPH3 | -0,317550057 |
| ANP32A | -0,315048635 |
| MED27 | -0,313143576 |
| PA2G4 | -0,31207199 |
| SF3B3 | -0,311863679 |
| HNRNPM | -0,311780647 |
| KIAA0020 | -0,311769566 |
| C9orf114 | -0,311605089 |
| HNRNPR | -0,307811364 |
| MRE11A | -0,307417133 |
| TTC33 | -0,307174056 |
| C2orf44 | -0,300951227 |
| PDAP1 | -0,300509748 |
| AKT1 | -0,300054401 |
| SDCCAG3 | -0,29615538 |
| WDR12 | -0,295096678 |
| FUBP1 | -0,294266401 |
| TAF6 | -0,290059763 |
| SLC25A51 | -0,288499004 |
| HNRNPH1 | -0,287497977 |
| TFAM | -0,287468175 |
| GABPB1 | -0,287289063 |
| CREB1 | -0,287116951 |
| NSUN2 | -0,286045763 |
| NCOA5 | -0,284218838 |
| EWSR1 | -0,283765598 |
| NUP50 | -0,283637038 |
| TSNAX | -0,283417069 |
| PPAT | -0,281538135 |
| ILF3 | -0,280306185 |
| NIP7 | -0,278486214 |
| HNRNPUL2 | -0,27814224 |
| BYSL | -0,277854261 |

| | |
|---|---|
| MTF2 | -0,27739736 |
| CCT5 | -0,277197626 |
| LSM12 | -0,273987254 |
| DR1 | -0,270576109 |
| MRPL42 | -0,269280811 |
| UBE2K | -0,268788359 |
| LSM14A | -0,268717567 |
| CBX3 | -0,267958069 |
| MAT2A | -0,267358165 |
| ZRANB2 | -0,267090443 |
| SRSF10 | -0,26557541 |
| ATAD1 | -0,262817798 |
| MPRIP | -0,260403428 |
| CARM1 | -0,260141561 |
| BLMH | -0,257950709 |
| ACTR2 | -0,25786633 |
| SNRNP40 | -0,254062935 |
| ABCE1 | -0,253881921 |
| STK35 | -0,253678501 |
| SPIDR | -0,253594616 |
| SERBP1 | -0,250668161 |
| STAM | -0,248468307 |
| HIRA | -0,246906641 |
| HNRNPU | -0,244841927 |
| DAZAP1 | -0,243981978 |
| CPSF3 | -0,243542386 |
| NUDCD1 | -0,243206144 |
| FAM210A | -0,242402172 |
| PPP1R8 | -0,242014076 |
| CPSF6 | -0,237890375 |
| TFCP2 | -0,234396276 |
| SF3A3 | -0,233179425 |
| HAUS2 | -0,228367805 |
| SRF | -0,221293197 |
| LEO1 | -0,220931086 |
| NONO | -0,219601351 |
| PCNP | -0,21786925 |
| SYNCRIP | -0,215750532 |
| NRAS | -0,212779754 |
| THUMPD1 | -0,211427783 |
| RNPS1 | -0,207032912 |
| GTPBP4 | -0,205096639 |
| CDC123 | -0,194097337 |
| PITPNB | -0,185785986 |
| RAVER1 | -0,176385949 |
| WBP11 | -0,166448471 |
| KHDRBS1 | -0,164665512 |

| | |
|---|---|
| ATXN2L | -0,157866285 |
| AKT2 | -0,153035792 |
| ZNF207 | -0,151296938 |

Up-regulated

| Gene | log2FoldChange |
|---|---|
| GPBP1 | 0,179066855 |
| TMBIM6 | 0,199276014 |
| UBC | 0,209094956 |
| SNX17 | 0,25197902 |
| ZKSCAN5 | 0,253953306 |
| C9orf156 | 0,254478608 |
| SQRDL | 0,259028717 |
| NDEL1 | 0,266253807 |
| SPG7 | 0,269191442 |
| TMEM115 | 0,273171189 |
| ARL2BP | 0,274456701 |
| COQ5 | 0,28227352 |
| SNAPIN | 0,283147497 |
| ASPSCR1 | 0,293232865 |
| CCDC22 | 0,30076485 |
| CINP | 0,303480176 |
| MGAT4B | 0,305428324 |
| NR1H2 | 0,305549829 |
| NDUFA4 | 0,311315334 |
| KIAA1191 | 0,313273853 |
| ITFG2 | 0,317875412 |
| SYNGR2 | 0,318577766 |
| DEDD2 | 0,319045021 |
| B4GALT7 | 0,327183626 |
| R3HDM4 | 0,329359259 |
| TM2D3 | 0,330183097 |
| ZNF761 | 0,344056185 |
| ZNF717 | 0,349973424 |
| PNPLA8 | 0,351699975 |
| EMC3 | 0,357530178 |
| ANKRD42 | 0,359686002 |
| TCTA | 0,362925215 |
| NDUFB2 | 0,365871878 |
| RNF14 | 0,371488434 |
| C19orf60 | 0,373407639 |
| CD99L2 | 0,373897033 |
| ORMDL2 | 0,380335303 |
| BAD | 0,385604917 |
| PARP6 | 0,390301558 |
| TMC6 | 0,397735638 |

| | |
|---|---|
| ZNF18 | 0,403280189 |
| PDE4DIP | 0,40427584 |
| MFSD11 | 0,406371587 |
| TMEM129 | 0,406970298 |
| FBXL15 | 0,409599968 |
| POLE4 | 0,413665152 |
| FAM98C | 0,419866877 |
| CCM2 | 0,431687012 |
| RMND5B | 0,434931873 |
| WDR45 | 0,439467946 |
| MTIF3 | 0,443617305 |
| PAK1 | 0,444503822 |
| TRPT1 | 0,44534882 |
| SERINC1 | 0,445743159 |
| ZNF671 | 0,453578901 |
| TMED9 | 0,464015731 |
| GNS | 0,465882783 |
| WDR83OS | 0,466177282 |
| PLA2G6 | 0,466632511 |
| TMEM216 | 0,466985824 |
| TPP1 | 0,472377255 |
| KPTN | 0,473817969 |
| LOC100131564 | 0,486787645 |
| IFT43 | 0,493751186 |
| SLC46A3 | 0,494004461 |
| ZNF700 | 0,494225053 |
| TPT1-AS1 | 0,494371119 |
| PLA2G15 | 0,495872847 |
| PSTPIP1 | 0,497234298 |
| KCTD21 | 0,497654194 |
| LOC285074 | 0,4994021 |
| EVI5 | 0,501755839 |
| PGAP3 | 0,503574612 |
| DLEU1 | 0,504804516 |
| PHTF1 | 0,514603403 |
| CCDC159 | 0,524519161 |
| TBC1D17 | 0,529338359 |
| PSENEN | 0,529991417 |
| TMEM99 | 0,532242163 |
| LIPT1 | 0,533560477 |
| CHPF2 | 0,533778623 |
| ACADM | 0,535654522 |
| PHF1 | 0,53650866 |
| NUDT17 | 0,54069888 |
| FLJ37453 | 0,54260512 |
| BIN1 | 0,548284219 |
| CYB5D2 | 0,559042317 |

| | |
|---|---|
| PNPLA4 | 0,559175501 |
| SGSH | 0,562063463 |
| GM2A | 0,574749745 |
| SLC31A2 | 0,578986153 |
| H1FX | 0,579506583 |
| LYSMD1 | 0,581430976 |
| TCAF2 | 0,583032423 |
| GLT8D1 | 0,585066405 |
| SPATA2L | 0,593172974 |
| DHRS12 | 0,611162473 |
| VAT1 | 0,613400512 |
| C2orf81 | 0,618423613 |
| IGFLR1 | 0,622942195 |
| C5orf45 | 0,629883031 |
| DGKQ | 0,630661218 |
| NDNL2 | 0,637426347 |
| SQSTM1 | 0,655592913 |
| ZNF559 | 0,659155845 |
| WASH3P | 0,6616276 |
| PPP2R5B | 0,667897385 |
| ZNF837 | 0,719438836 |
| MAP3K14-AS1 | 0,721112007 |
| FLJ20021 | 0,722141401 |
| LOC728743 | 0,72578161 |
| JOSD2 | 0,741221238 |
| MIR22HG | 0,775774481 |
| ABCA2 | 0,784173897 |
| SDCBP2-AS1 | 0,809526402 |
| MYL5 | 0,81621216 |
| CTTN | 0,827992972 |
| TYMP | 0,831850757 |
| ZNF763 | 0,836741024 |
| ZNF699 | 0,84655015 |
| SCAMP1-AS1 | 0,881764631 |
| B3GALT4 | 0,887743786 |
| ABHD4 | 0,896938644 |
| GPNMB | 0,921696884 |
| PKIG | 0,926865177 |
| GABARAPL1 | 0,931549418 |
| EMILIN2 | 0,951420352 |
| TMEM44-AS1 | 0,956187168 |
| NPIPB4 | 0,971274593 |
| KIAA0226L | 0,991814487 |
| DDR1 | 0,996368592 |
| RUSC2 | 1,51855E+12 |
| ETV2 | 1,14632E+13 |
| GSTA4 | 1,5133E+13 |

| | |
|---|---|
| TPTE2 | 1,59254E+13 |
| HHLA2 | 2,33951E+13 |
| PRODH | 2,34508E+13 |
| CX3CL1 | 4,01769E+13 |
| CPEB3 | 1,00112E+14 |
| SLC26A11 | 1,02564E+14 |
| ANGPTL2 | 1,03295E+14 |
| OSER1-AS1 | 1,03853E+14 |
| RAP2C-AS1 | 1,04362E+14 |
| LIN7B | 1,06578E+14 |
| ZNF880 | 1,07334E+14 |
| C14orf79 | 1,08429E+14 |
| MIR5195 | 1,0846E+14 |
| LOC100507283 | 1,09463E+14 |
| SLC16A5 | 1,09877E+14 |
| KLHL6 | 1,1056E+14 |
| LY6G5C | 1,10722E+14 |
| UXT-AS1 | 1,1103E+14 |
| LOC728392 | 1,15851E+14 |
| TTC22 | 1,22495E+14 |
| TTC39A | 1,37953E+14 |
| GPR157 | 1,38069E+14 |
| SLC7A7 | 1,38112E+14 |
| YTHDF3-AS1 | 1,39581E+14 |
| RRAD | 1,4116E+14 |
| MIR3064 | 1,41531E+14 |
| HEPH | 1,47215E+14 |
| HMOX1 | 1,48438E+14 |
| PVRL4 | 1,50655E+14 |
| PFN4 | 1,51551E+14 |
| PTPRF | 1,53362E+14 |
| KIAA0895 | 1,55647E+14 |
| ITGAM | 1,57596E+14 |
| DNM1P46 | 1,62663E+14 |
| SH3BGRL2 | 1,64137E+14 |
| CHRNE | 1,65273E+14 |
| CACNA1E | 1,65475E+14 |
| FAM167B | 1,66498E+14 |
| TMEM51 | 1,66767E+14 |
| C20orf194 | 1,70689E+14 |
| PLEKHG6 | 1,7102E+14 |
| EDN1 | 1,71541E+14 |
| POU4F1 | 1,7326E+14 |
| GABBR1 | 1,73868E+14 |
| HCN2 | 1,87329E+14 |
| LARP6 | 1,90091E+14 |
| GUCY2C | 1,98495E+14 |

| | |
|---|---|
| DENND2C | 2,05185E+14 |
| INCA1 | 2,05339E+14 |
| SEMA3B | 2,08052E+14 |
| PIWIL2 | 2,21341E+14 |
| TTC7B | 2,26177E+14 |
| SGK2 | 2,30811E+14 |
| HPCAL4 | 2,3325E+14 |
| LINC00540 | 2,37784E+14 |
| NRN1 | 2,38082E+14 |
| PCDH8 | 2,40565E+14 |
| OSBPL6 | 2,43551E+14 |
| LINC00925 | 2,43788E+14 |
| HAL | 2,50636E+14 |
| UTS2R | 2,51E+14 |
| ADGRA3 | 2,70363E+14 |
| DRC7 | 2,72599E+14 |
| SEPP1 | 2,83571E+14 |
| RASD1 | 2,8955E+14 |
| LOC730102 | 2,90396E+14 |
| ARHGEF10L | 2,99544E+14 |
| TCTE1 | 3,02987E+14 |
| PAX9 | 3,34621E+14 |
| FAM46B | 3,35461E+14 |
| EGFL6 | 3,58334E+14 |
| LOC100506860 | 3,58852E+14 |
| PAX3 | 3,7936E+14 |
| KIF17 | 3,79976E+14 |
| INHBB | 4,01232E+14 |
| GUSBP3 | 4,03097E+14 |
| AHSG | 4,10712E+14 |
| TMEM176B | 5,25843E+14 |
| LINC00221 | 9,60697E+14 |

Supplementary Table 8. Differentially expressed genes (up- and downregulated) in EP versus CdLS cell lines, both carrying missense variants.

Down-regulated

| Gene | log2FoldChange |
|---|---|
| LINC01115 | -7,49312E+14 |
| TLE1 | -6,93177E+14 |
| GZMH | -6,61912E+14 |
| NETO1 | -6,09623E+14 |
| EML5 | -5,94021E+14 |
| KCTD15 | -5,93567E+14 |
| CBR3 | -5,62118E+14 |
| ZFX-AS1 | -5,56557E+14 |
| CRTAM | -5,16042E+14 |
| MAGI2 | -4,7322E+14 |
| GRIA1 | -4,6603E+14 |
| SNORD47 | -4,53412E+14 |
| AXIN2 | -4,34965E+14 |
| C17orf97 | -4,17491E+14 |
| SPTSSB | -3,49986E+14 |
| IL17RC | -3,44633E+14 |
| DLSTP1 | -2,70394E+14 |
| LOC729737 | -2,6578E+14 |
| SPNS3 | -2,6196E+14 |
| DOCK4 | -2,10965E+14 |
| PHKA1 | -2,09937E+14 |
| FCGR2C | -2,09611E+14 |
| MAN1C1 | -2,04229E+14 |
| KLHL13 | -1,9062E+14 |
| HLA-DQB1 | -1,86144E+14 |
| PALD1 | -1,86005E+14 |
| ZNF818P | -1,74512E+14 |
| NLRP2 | -1,66248E+14 |
| FAM86B3P | -1,63769E+14 |
| HLA-DQA1 | -1,54727E+14 |
| AMZ1 | -1,53514E+14 |
| WDR17 | -1,51911E+14 |
| ITGB3 | -1,49035E+14 |
| STC2 | -1,27302E+14 |
| KCTD12 | -1,11262E+14 |
| ZNF584 | -1,08477E+14 |
| MYB | -1,02139E+14 |
| JAKMIP1 | -3,19922E+13 |
| EIF5AL1 | -2,38558E+13 |
| CTH | -0,998662919 |

| | |
|---|---|
| PHGDH | -0,979837369 |
| PSAT1 | -0,968122819 |
| UHRF1 | -0,904708037 |
| CCDC126 | -0,901470442 |
| HDAC4 | -0,898122368 |
| NDC1 | -0,857162636 |
| SPAG16 | -0,849866252 |
| PKP4 | -0,832765622 |
| MCM7 | -0,827767061 |
| TFAP4 | -0,807011831 |
| TAPBPL | -0,805193861 |
| SLC25A10 | -0,802819493 |
| SMC4 | -0,797174425 |
| RBL1 | -0,79430949 |
| SLC19A1 | -0,793968727 |
| SMC2 | -0,792414163 |
| LZIC | -0,792189711 |
| DNAJC15 | -0,786470876 |
| CEP78 | -0,781968075 |
| MMS22L | -0,772762245 |
| POLA1 | -0,770340951 |
| MCM3 | -0,758595957 |
| TMPO | -0,757591077 |
| RIF1 | -0,754040057 |
| ARHGAP11A | -0,753330268 |
| MCM4 | -0,736658475 |
| MZT1 | -0,735469051 |
| SLC4A7 | -0,726032675 |
| NUP155 | -0,719818523 |
| PRDM10 | -0,719268622 |
| PFAS | -0,71746258 |
| XPO4 | -0,713952009 |
| SRSF1 | -0,706830422 |
| MZT2A | -0,706150832 |
| CBFB | -0,704266057 |
| CEP57L1 | -0,69979738 |
| ARHGAP19 | -0,695034906 |
| CASC5 | -0,692039054 |
| MKI67 | -0,691483333 |
| WDR76 | -0,689425128 |
| LRRC58 | -0,687356993 |
| CHML | -0,684050777 |
| MCM5 | -0,675909032 |
| ST3GAL1 | -0,67478489 |
| MCMBP | -0,670748887 |
| MARCKS | -0,666214144 |
| COLGALT1 | -0,664882004 |

| | |
|---|---|
| PAG1 | -0,662280758 |
| ITPRIPL1 | -0,66082516 |
| ANP32E | -0,654057258 |
| FAM122B | -0,64563338 |
| TOP2A | -0,644914381 |
| IRAK1 | -0,642328471 |
| WDR4 | -0,641819102 |
| NAA15 | -0,640278059 |
| WHSC1 | -0,631384427 |
| CENPU | -0,631322422 |
| MIPEP | -0,629554865 |
| TCOF1 | -0,62097737 |
| MFNG | -0,620383692 |
| XPOT | -0,616611679 |
| MCM2 | -0,615932743 |
| RNF138 | -0,610128797 |
| MTHFD1L | -0,608551052 |
| HNRNPD | -0,601290636 |
| NAA50 | -0,597949516 |
| SLC7A1 | -0,596215735 |
| NCL | -0,595900491 |
| SLC7A5 | -0,595667647 |
| NOL8 | -0,590613007 |
| APIP | -0,590031205 |
| HNRNPA3 | -0,576455512 |
| BRIP1 | -0,573440444 |
| RFC2 | -0,572909455 |
| OSBPL8 | -0,572878708 |
| PARPBP | -0,57267797 |
| RAI1 | -0,569237959 |
| PRKDC | -0,568630678 |
| DIAPH3 | -0,56840732 |
| SMC1A | -0,567399222 |
| HNRNPA2B1 | -0,566760236 |
| DDX21 | -0,56556733 |
| GPR180 | -0,559515283 |
| AMD1 | -0,558464895 |
| DHX33 | -0,558084017 |
| NUP160 | -0,557786124 |
| C10orf2 | -0,552601798 |
| MEF2A | -0,546614095 |
| SUZ12 | -0,541928541 |
| NUCKS1 | -0,535665488 |
| MAZ | -0,530164395 |
| TCERG1 | -0,527909101 |
| LIG3 | -0,525520194 |
| BAG4 | -0,524720734 |

| | |
|---|---|
| GMFB | -0,521276696 |
| DHX9 | -0,511409529 |
| PSPH | -0,5098762 |
| ANP32B | -0,508083613 |
| ESF1 | -0,507905886 |
| DOCK8 | -0,506175699 |
| HK2 | -0,504540134 |
| TTLL12 | -0,503033128 |
| TP53 | -0,498264991 |
| DOCK7 | -0,496504844 |
| SRSF10 | -0,49390015 |
| TRA2B | -0,493535436 |
| XPO1 | -0,492474328 |
| TTLL4 | -0,49242609 |
| SF3B3 | -0,491139379 |
| SSRP1 | -0,48705857 |
| LIG1 | -0,486436385 |
| FBXO41 | -0,485087738 |
| HDGF | -0,478749471 |
| LPGAT1 | -0,473975854 |
| BOP1 | -0,470458557 |
| RUVBL1 | -0,469690828 |
| TMEM123 | -0,468823805 |
| NUP153 | -0,464783258 |
| BAZ1A | -0,463753307 |
| KIAA0196 | -0,462732666 |
| KPNA3 | -0,462006717 |
| SET | -0,46083482 |
| PPP1CB | -0,459418269 |
| ZNHIT6 | -0,453222226 |
| NOL11 | -0,453206888 |
| NOLC1 | -0,452368264 |
| CCT5 | -0,44384123 |
| RNF219 | -0,440263208 |
| HNRNPH3 | -0,437512944 |
| IPO7 | -0,437440272 |
| UCHL5 | -0,437128039 |
| ARMC10 | -0,436448853 |
| KHSRP | -0,433488124 |
| STX7 | -0,433465753 |
| GPATCH4 | -0,433201232 |
| MBNL1 | -0,430874311 |
| PURB | -0,430318723 |
| TCP1 | -0,424575147 |
| RBM14 | -0,424014547 |
| CELF1 | -0,423737092 |
| HNRNPM | -0,422172466 |

| | |
|---|---|
| WDR36 | -0,422043224 |
| LARP4 | -0,42126076 |
| IDH3A | -0,420450265 |
| KPNB1 | -0,420208835 |
| SNRNP27 | -0,419994913 |
| CBX3 | -0,419118741 |
| HNRNPR | -0,418360907 |
| WDR3 | -0,413650576 |
| ILF3 | -0,41108704 |
| HSPD1 | -0,41105277 |
| EIF1AX | -0,410583855 |
| BAZ1B | -0,409584366 |
| ARHGAP18 | -0,408361908 |
| LSM14A | -0,407470969 |
| IPO4 | -0,402176896 |
| RBBP8 | -0,400975812 |
| MPRIP | -0,398891077 |
| HNRNPH1 | -0,396581442 |
| KIAA0020 | -0,390772644 |
| HSP90AA1 | -0,390421524 |
| RBM25 | -0,388539108 |
| PES1 | -0,384958896 |
| NUP50 | -0,374819341 |
| SERBP1 | -0,372476699 |
| CACTIN | -0,369392874 |
| NPM1 | -0,36850778 |
| C15orf39 | -0,35855081 |
| SRRM1 | -0,354573492 |
| SENP1 | -0,353357761 |
| IARS | -0,353102556 |
| NUPL1 | -0,3504704 |
| DAZAP1 | -0,346783096 |
| HNRNPU | -0,345991534 |
| ABCF2 | -0,345937112 |
| UBE2K | -0,341531823 |
| ZRANB2 | -0,340712929 |
| EIF2S2 | -0,339664288 |
| NACC1 | -0,336104371 |
| SF3A3 | -0,331109719 |

Up-regulated

| Gene | log2FoldChange |
|---|---|
| CTSA | 0,384553823 |
| SLC25A1 | 0,389285919 |
| GPX4 | 0,410899146 |
| TRAFD1 | 0,424447721 |

| | |
|---|---|
| NCSTN | 0,448821905 |
| SHISA5 | 0,453262697 |
| NDEL1 | 0,455169458 |
| PSENEN | 0,460976937 |
| IRF9 | 0,466435819 |
| CCND2 | 0,475330582 |
| F11R | 0,478185587 |
| IL2RG | 0,482963624 |
| HLA-E | 0,486188913 |
| MCOLN2 | 0,493717168 |
| AVEN | 0,497362547 |
| CST3 | 0,502490068 |
| TRAF1 | 0,517186497 |
| PNOC | 0,527698073 |
| EHMT1 | 0,539252259 |
| SKAP1 | 0,543128761 |
| TTYH3 | 0,545837538 |
| ICAM3 | 0,545985471 |
| ACP2 | 0,546309276 |
| SERINC1 | 0,547289579 |
| THEMIS2 | 0,548280521 |
| PVT1 | 0,578494413 |
| LAPTM5 | 0,579700429 |
| ZNF581 | 0,584963139 |
| INPP1 | 0,596203513 |
| BBC3 | 0,599580867 |
| CD99L2 | 0,603136887 |
| SPRYD4 | 0,604025768 |
| DTX3 | 0,608800272 |
| ALOX5AP | 0,612229331 |
| LIPA | 0,616615429 |
| TMEM164 | 0,618431558 |
| MICAL1 | 0,620282441 |
| PIGV | 0,624810353 |
| ZNF331 | 0,629877047 |
| CFLAR | 0,636966145 |
| RGS20 | 0,640097101 |
| MVD | 0,640864208 |
| RAB9A | 0,643151636 |
| ACADM | 0,652926943 |
| DNAJB2 | 0,65918492 |
| HAAO | 0,659373664 |
| B4GALT3 | 0,668237155 |
| FCER2 | 0,689213705 |
| FAM117A | 0,703190049 |
| SERPINB8 | 0,714867166 |
| TMOD1 | 0,718556538 |

| | |
|---|---|
| TM7SF2 | 0,725378884 |
| SGSH | 0,727856603 |
| MKNK2 | 0,730338218 |
| ARSA | 0,747934452 |
| CRTC1 | 0,755508352 |
| IGFLR1 | 0,759851953 |
| ZC3H12D | 0,765013324 |
| RGL1 | 0,779926731 |
| CCDC102A | 0,781546323 |
| CCDC64 | 0,781846696 |
| PNKD | 0,790636788 |
| TNFSF13B | 0,797379593 |
| ATHL1 | 0,799672081 |
| IKZF1 | 0,805957917 |
| RASGRP1 | 0,809690346 |
| CDKN1A | 0,816454132 |
| ATF3 | 0,823814064 |
| LGALS3 | 0,830816468 |
| ABHD4 | 0,832787057 |
| CHRNB1 | 0,84768966 |
| FDXR | 0,850291947 |
| HMCES | 0,851230807 |
| TBC1D17 | 0,857495232 |
| PITRM1 | 0,87425056 |
| FBXO44 | 0,876883032 |
| CD83 | 0,901424804 |
| RN7SK | 0,903248363 |
| ISCU | 0,9056246 |
| PHLDA3 | 0,907688788 |
| SOCS1 | 0,908678614 |
| CHDH | 0,911694008 |
| ASRGL1 | 0,91517423 |
| KLK1 | 0,921746687 |
| CTTN | 0,922670371 |
| YPEL3 | 0,935515301 |
| STARD10 | 0,95129929 |
| RTN2 | 0,955289226 |
| TUBB2A | 0,957732164 |
| PIM2 | 0,96023619 |
| HHAT | 0,986226803 |
| ENDOV | 1,0039E+13 |
| TMEM25 | 1,15974E+13 |
| PLXNB1 | 1,23626E+13 |
| CAB39L | 1,24806E+13 |
| CNR1 | 1,26652E+13 |
| B3GALT4 | 1,28699E+13 |
| B3GNT9 | 1,29385E+13 |

| | |
|---|---|
| HECW2 | 1,36148E+13 |
| PLTP | 1,37574E+13 |
| ANXA1 | 1,82192E+13 |
| BCAS1 | 2,05096E+13 |
| SLC32A1 | 2,44528E+13 |
| ZNF503 | 2,44947E+13 |
| STEAP2 | 2,63505E+13 |
| MSRB3 | 2,79704E+13 |
| SNPH | 2,90132E+13 |
| ESPN | 4,39682E+13 |
| SDPR | 6,64161E+13 |
| AOC1 | 1,00279E+14 |
| QPRT | 1,0091E+14 |
| ASTN2 | 1,02234E+14 |
| KIAA1217 | 1,04637E+14 |
| GABARAPL1 | 1,08344E+14 |
| MYL5 | 1,10241E+14 |
| ST3GAL6 | 1,11665E+14 |
| PKIG | 1,13491E+14 |
| SULF2 | 1,13669E+14 |
| MAP3K12 | 1,14642E+14 |
| SYNPO | 1,15293E+14 |
| CLECL1 | 1,16775E+14 |
| TNFSF4 | 1,16906E+14 |
| LACC1 | 1,17266E+14 |
| PMS2P5 | 1,18248E+14 |
| SDCBP2-AS1 | 1,20721E+14 |
| EVA1B | 1,22118E+14 |
| SHF | 1,22868E+14 |
| GAMT | 1,24572E+14 |
| SCAMP1-AS1 | 1,26357E+14 |
| SPATA20 | 1,28518E+14 |
| NTRK2 | 1,296E+14 |
| APOL1 | 1,3035E+14 |
| GPR15 | 1,32007E+14 |
| WNT10A | 1,32471E+14 |
| LINC00936 | 1,35197E+14 |
| ETV7 | 1,37009E+14 |
| CLIP3 | 1,37178E+14 |
| ABCA3 | 1,37198E+14 |
| TMEM140 | 1,38226E+14 |
| TK2 | 1,39383E+14 |
| KLHL6 | 1,39405E+14 |
| CCR7 | 1,42287E+14 |
| CKB | 1,4235E+14 |
| GAL3ST4 | 1,43763E+14 |
| GUCY1A3 | 1,44927E+14 |

| | |
|---|---|
| XXYLT1-AS2 | 1,45229E+14 |
| ADAM19 | 1,47516E+14 |
| S100A4 | 1,49702E+14 |
| SLC7A7 | 1,53637E+14 |
| ZNF880 | 1,53871E+14 |
| PERP | 1,5482E+14 |
| DOK4 | 1,57577E+14 |
| ST3GAL6-AS1 | 1,58159E+14 |
| IGFBP4 | 1,58517E+14 |
| FAM46A | 1,58799E+14 |
| CHST6 | 1,59808E+14 |
| GAREML | 1,60466E+14 |
| MOXD1 | 1,62391E+14 |
| MT2A | 1,6443E+14 |
| CDKN2A | 1,64929E+14 |
| PLK2 | 1,65587E+14 |
| CSPG4 | 1,6677E+14 |
| LNX1 | 1,6804E+14 |
| F2R | 1,70841E+14 |
| LTBR | 1,72081E+14 |
| MAB21L3 | 1,73492E+14 |
| TTC22 | 1,74416E+14 |
| ABCA9 | 1,7489E+14 |
| CYP1B1 | 1,75789E+14 |
| ELF3 | 1,79125E+14 |
| LIPH | 1,81358E+14 |
| MAL | 1,83069E+14 |
| RRAD | 1,859E+14 |
| EMX1 | 1,91103E+14 |
| HCN2 | 1,92528E+14 |
| SPINT2 | 1,96425E+14 |
| EPHA2 | 1,97373E+14 |
| HES2 | 1,98092E+14 |
| SERPINB1 | 1,99894E+14 |
| ENPP2 | 2,0518E+14 |
| GLIS3 | 2,05922E+14 |
| TLN2 | 2,08662E+14 |
| CLEC2B | 2,09397E+14 |
| OSBPL6 | 2,1087E+14 |
| TCN2 | 2,12553E+14 |
| C10orf10 | 2,14355E+14 |
| CYSLTR2 | 2,15994E+14 |
| PDE6G | 2,1643E+14 |
| STEAP1 | 2,1927E+14 |
| CXCR5 | 2,19998E+14 |
| JUP | 2,27343E+14 |
| C20orf194 | 2,28079E+14 |

| | |
|---|---|
| EPHB1 | 2,28595E+14 |
| TIGIT | 2,28694E+14 |
| SERPINB10 | 2,29404E+14 |
| ITGAM | 2,354E+14 |
| EDN1 | 2,36738E+14 |
| IFNG | 2,37152E+14 |
| LOC100190986 | 2,37491E+14 |
| LAG3 | 2,38014E+14 |
| PLEKHG6 | 2,39778E+14 |
| TNFRSF4 | 2,41079E+14 |
| MUC13 | 2,4137E+14 |
| NAALADL1 | 2,44462E+14 |
| MAP7D2 | 2,52512E+14 |
| ABCA12 | 2,52872E+14 |
| ST14 | 2,58061E+14 |
| GTF2IRD2 | 2,59027E+14 |
| SYTL2 | 2,59253E+14 |
| ZNF578 | 2,59406E+14 |
| HLA-DQA2 | 2,5996E+14 |
| ADGRA3 | 2,63012E+14 |
| DLGAP1 | 2,63472E+14 |
| LINC00176 | 2,6573E+14 |
| ANO3 | 2,67899E+14 |
| DDR2 | 2,69253E+14 |
| RUSC2 | 2,69692E+14 |
| SEPP1 | 2,82432E+14 |
| CACNB2 | 2,8313E+14 |
| POU4F1 | 2,84463E+14 |
| FAM167B | 2,86236E+14 |
| PCDHGB5 | 2,88506E+14 |
| CALD1 | 2,88992E+14 |
| DTHD1 | 2,90801E+14 |
| OR2T3 | 2,90935E+14 |
| MOCS1 | 2,91326E+14 |
| PLCL1 | 2,96139E+14 |
| HOMER3 | 3,02053E+14 |
| LINC01150 | 3,02983E+14 |
| RYR3 | 3,04053E+14 |
| PHEX | 3,06481E+14 |
| LINC01258 | 3,16131E+14 |
| PRODH | 3,16626E+14 |
| HPCAL4 | 3,16888E+14 |
| LOC101930010 | 3,24743E+14 |
| AMBP | 3,26739E+14 |
| NINL | 3,27754E+14 |
| LINC00987 | 3,30934E+14 |
| SLIT1 | 3,35207E+14 |

| | |
|---|---|
| ASPA | 3,40634E+14 |
| SLC4A4 | 3,45134E+14 |
| HHLA2 | 3,52924E+14 |
| CREB3L3 | 3,58671E+14 |
| NAP1L3 | 3,67292E+14 |
| LINC01320 | 3,68264E+14 |
| POMC | 3,76991E+14 |
| LOC730102 | 3,91074E+14 |
| LHFPL3-AS2 | 3,97057E+14 |
| LRRN3 | 3,98183E+14 |
| WFDC2 | 4,00476E+14 |
| WNT7B | 4,07489E+14 |
| CPA4 | 4,34219E+14 |
| MIR4507 | 4,51656E+14 |
| LINC00689 | 5,20609E+14 |
| KIF17 | 5,30967E+14 |
| THEMIS | 5,3812E+14 |
| CX3CL1 | 5,49747E+14 |
| OR5H6 | 6,2858E+14 |
| CLEC4C | 6,33978E+14 |
| ACTN3 | 6,88642E+14 |

Supplementary Table 9. Dysregulated genes (both downregulated and upregulated) identified upon comparison of ataluren EP1-treated cells versus untreated controls.

Down-regulated

| Gene | log2FoldChange2 |
|---|---|
| MIR4785 | -6,3E+14 |
| LOC283440 | -5,7E+14 |
| SNORD18B | -5,4E+14 |
| LDB2 | -5,2E+14 |
| EFCAB9 | -5E+14 |
| C1orf189 | -5E+14 |
| CASC19 | -5E+14 |
| PDCD1 | -4,8E+14 |
| LOC101929473 | -4,8E+14 |
| LOC102723517 | -4,8E+14 |
| ABCG1 | -3,1E+14 |
| CXCR2P1 | -2,9E+14 |
| RBM5-AS1 | -2,9E+14 |
| KCNQ5-IT1 | -2,8E+14 |
| CCL3L1 | -2,7E+14 |
| LST1 | -2,5E+14 |
| INSM1 | -2,4E+14 |
| LOC100130872 | -2,4E+14 |
| XCL1 | -2,2E+14 |
| CYP51A1-AS1 | -2,1E+14 |
| SREBF1 | -2,1E+14 |
| OLMALINC | -2E+14 |
| C1orf228 | -2E+14 |
| PTGER2 | -2E+14 |
| RUFY4 | -1,9E+14 |
| LINC01176 | -1,9E+14 |
| PIPOX | -1,9E+14 |
| ULBP2 | -1,8E+14 |
| MIR6774 | -1,8E+14 |
| TAS2R4 | -1,8E+14 |
| MIR4517 | -1,7E+14 |
| SCD | -1,7E+14 |
| NPW | -1,7E+14 |
| PNPLA3 | -1,7E+14 |
| LOC100506801 | -1,6E+14 |
| MST1P2 | -1,6E+14 |
| TMEM191B | -1,6E+14 |
| GRAMD1B | -1,6E+14 |
| KLHL29 | -1,6E+14 |
| LOC100506457 | -1,5E+14 |

| | |
|---|---|
| TSNAXIP1 | -1,5E+14 |
| SCML1 | -1,5E+14 |
| SNORD14D | -1,5E+14 |
| EDN1 | -1,5E+14 |
| KCNA1 | -1,5E+14 |
| LOC102724596 | -1,4E+14 |
| GPR84 | -1,4E+14 |
| EGR3 | -1,4E+14 |
| SETBP1 | -1,4E+14 |
| SLC29A2 | -1,4E+14 |
| PDGFA | -1,4E+14 |
| LOC646471 | -1,4E+14 |
| HOXB9 | -1,4E+14 |
| LINC01126 | -1,4E+14 |
| DHRS9 | -1,4E+14 |
| THY1 | -1,4E+14 |
| AK1 | -1,4E+14 |
| SNHG25 | -1,3E+14 |
| PCYT1B | -1,3E+14 |
| TPTE2P5 | -1,3E+14 |
| HES6 | -1,2E+14 |
| ZBED3-AS1 | -1,2E+14 |
| HIST4H4 | -1,2E+14 |
| ALDH8A1 | -1,2E+14 |
| SPON2 | -1,2E+14 |
| PCSK4 | -1,2E+14 |
| CD4 | -1,2E+14 |
| FAM129C | -1,2E+14 |
| SEMA6C | -1,2E+14 |
| RAB33A | -1,2E+14 |
| CHRNA10 | -1,2E+14 |
| LINC00106 | -1,2E+14 |
| SSSCA1-AS1 | -1,2E+14 |
| LINC00504 | -1,2E+14 |
| CFAP46 | -1,2E+14 |
| DLGAP4-AS1 | -1,1E+14 |
| LOC105377348 | -1,1E+14 |
| MYO1F | -1,1E+14 |
| C1QTNF6 | -1,1E+14 |
| AK4 | -1,1E+14 |
| FCRL5 | -1,1E+14 |
| CCDC24 | -1,1E+14 |
| TMEM145 | -1,1E+14 |
| EFEMP2 | -1,1E+14 |
| NPIPB15 | -1,1E+14 |
| BMPR1A | -1,1E+14 |

| | |
|---|---|
| SMPDL3B | -1,1E+14 |
| FCRL3 | -1,1E+14 |
| MC1R | -1,1E+14 |
| NRARP | -1,1E+14 |
| PLAUR | -1,1E+14 |
| MIR34A | -1,1E+14 |
| MOK | -1,1E+14 |
| GATA3-AS1 | -1,1E+14 |
| PLEKHG6 | -1,1E+14 |
| SYT17 | -1,1E+14 |
| NPHP1 | -1,1E+14 |
| WHAMMP1 | -1,1E+14 |
| SNAI3-AS1 | -1E+14 |
| SNHG19 | -1E+14 |
| HCN3 | -1E+14 |
| CCDC74A | -1E+14 |
| FBXL8 | -1E+14 |
| IL13RA1 | -1E+14 |
| NPFF | -1E+14 |
| CFHR4 | -5E+13 |
| C11orf91 | -5E+13 |
| LOC100130417 | -4,9E+13 |
| H1FX-AS1 | -2,5E+13 |
| LOC103908605 | -2,2E+13 |
| ZDHHC1 | -1,8E+13 |
| SNORA63 | -1,5E+13 |
| GALM | -1,3E+13 |
| LINC00957 | -1,2E+13 |
| FSCN2 | -1,2E+13 |
| RIMKLB | -1,1E+13 |
| NPPA-AS1 | -1,1E+13 |
| ACSS2 | -1,1E+13 |
| HIST1H1E | -1E+13 |
| AK7 | -1E+13 |
| LOC401557 | -5,1E+12 |
| GRIN1 | -1,6E+12 |
| NR4A1 | -1,2E+12 |
| PALD1 | -1,2E+12 |
| ANKRD6 | -0,99729 |
| BMS1P5 | -0,99588 |
| TMEM198 | -0,99401 |
| KLHDC1 | -0,98807 |
| CLCN6 | -0,98612 |
| TTLL3 | -0,98312 |
| PFKFB4 | -0,98254 |
| C5AR1 | -0,98082 |

| | |
|---|---|
| LINC01311 | -0,98021 |
| GUCA1B | -0,97816 |
| TMEM9B-AS1 | -0,97179 |
| EPHB2 | -0,97155 |
| ACAD11 | -0,96445 |
| MFSD2A | -0,96381 |
| CPNE7 | -0,96024 |
| MIR4292 | -0,95737 |
| ARHGAP24 | -0,9571 |
| GEM | -0,95511 |
| MIR3064 | -0,95479 |
| URB1-AS1 | -0,94771 |
| CD69 | -0,94597 |
| LBHD1 | -0,94098 |
| SNORD104 | -0,94009 |
| FER1L4 | -0,93252 |
| SNORD2 | -0,92929 |
| KIF6 | -0,92884 |
| KIAA0226L | -0,92837 |
| GABBR1 | -0,92188 |
| TUBB3 | -0,91684 |
| RBMS1 | -0,90975 |
| LOC100270804 | -0,90964 |
| MIR1914 | -0,90543 |
| LOC644656 | -0,9044 |
| ARHGAP39 | -0,89967 |
| ANKRD24 | -0,8973 |
| RARG | -0,89269 |
| TSPAN32 | -0,88891 |
| TPBG | -0,88889 |
| ETV7 | -0,88784 |
| PAN3-AS1 | -0,88729 |
| RGS3 | -0,88634 |
| MYCBPAP | -0,88533 |
| SPTBN5 | -0,87613 |
| WHAMMP3 | -0,87531 |
| GIPR | -0,87079 |
| TMEM44-AS1 | -0,8686 |
| RHOV | -0,86688 |
| TMEM150A | -0,86478 |
| COL4A3 | -0,86319 |
| DUOX1 | -0,85959 |
| MST1R | -0,85922 |
| HAPLN3 | -0,85667 |
| TGM1 | -0,85361 |
| LOC101927740 | -0,84712 |

| | |
|---|---|
| INTS6-AS1 | -0,83914 |
| RGS6 | -0,83764 |
| PRICKLE4 | -0,8357 |
| ZNRD1-AS1 | -0,82704 |
| BTNL9 | -0,82687 |
| CTC-338M12,4 | -0,81819 |
| ZBTB10 | -0,81789 |
| ANKRD13B | -0,81415 |
| LINC00494 | -0,81186 |
| TMEM136 | -0,80692 |
| PROCA1 | -0,80676 |
| RPSAP9 | -0,80364 |
| MIR22HG | -0,80029 |
| KIAA1875 | -0,79802 |
| CELF6 | -0,78288 |
| CCNI2 | -0,78074 |
| C8orf44 | -0,77889 |
| DBNDD1 | -0,77699 |
| MNDA | -0,77404 |
| SNHG9 | -0,77401 |
| COL9A2 | -0,76887 |
| ICAM5 | -0,76836 |
| IDUA | -0,76762 |
| FAM53A | -0,76638 |
| PPP1R32 | -0,76413 |
| DHRS2 | -0,76394 |
| HSD17B7 | -0,7619 |
| SLCO4C1 | -0,7615 |
| CORO1B | -0,76017 |
| HAGHL | -0,75547 |
| SLC26A11 | -0,75382 |
| ZSWIM6 | -0,75323 |
| LZTFL1 | -0,74866 |
| PLEKHG1 | -0,74704 |
| ARRDC3-AS1 | -0,74251 |
| SNORD58C | -0,74235 |
| CBLN3 | -0,7399 |
| C14orf79 | -0,73896 |
| CREB5 | -0,73833 |
| WBP1 | -0,73111 |
| CD72 | -0,72962 |
| TUBB2B | -0,72893 |
| GOLGA6L10 | -0,72862 |
| ZC3H12C | -0,72626 |
| C19orf71 | -0,72413 |
| QRICH2 | -0,72072 |

| | |
|---|---|
| AMY2B | -0,7198 |
| TCEB3-AS1 | -0,71901 |
| RPARP-AS1 | -0,71639 |
| AMT | -0,7148 |
| APTR | -0,71328 |
| TNFAIP2 | -0,71192 |
| TFAP2A-AS1 | -0,70991 |
| CAPN3 | -0,70807 |
| BCORP1 | -0,70455 |
| SERPINB1 | -0,7016 |
| NLGN2 | -0,69965 |
| LMO2 | -0,69948 |
| GSTM2 | -0,69706 |
| MAP3K14-AS1 | -0,69482 |
| MXI1 | -0,69435 |
| MORN2 | -0,6943 |
| RPL36A | -0,69316 |
| LOC102724814 | -0,69173 |
| NPDC1 | -0,69137 |
| PRR7 | -0,69006 |
| ZNF846 | -0,68737 |
| NPIPA1 | -0,68463 |
| C16orf93 | -0,68444 |
| RTKN | -0,68013 |
| PATL2 | -0,67845 |
| SAP25 | -0,67624 |
| ASMTL-AS1 | -0,67192 |
| LOC100506127 | -0,66885 |
| CD40 | -0,66871 |
| SENP8 | -0,66619 |
| LINC01138 | -0,66502 |
| ST20 | -0,66477 |
| CBWD3 | -0,66181 |
| C12orf77 | -0,66163 |
| SNHG11 | -0,65937 |
| KCNC4 | -0,65892 |
| C12orf79 | -0,65823 |
| PRORSD1P | -0,65396 |
| PAQR6 | -0,65359 |
| NT5M | -0,6488 |
| NPHP3 | -0,64747 |
| MZF1-AS1 | -0,64283 |
| FSTL3 | -0,64214 |
| SPAG4 | -0,64206 |
| NFYC-AS1 | -0,64023 |
| RDH14 | -0,63985 |

| | |
|---|---|
| PPT2 | -0,63955 |
| IFT20 | -0,63786 |
| RAB11B-AS1 | -0,63675 |
| HIST1H3E | -0,6361 |
| DNHD1 | -0,63609 |
| SRI | -0,63403 |
| TSPO | -0,63201 |
| MFSD10 | -0,62945 |
| IDH1 | -0,6285 |
| MERTK | -0,6279 |
| SNHG10 | -0,62502 |
| LINC01089 | -0,62321 |
| CD200R1 | -0,6228 |
| GPR89B | -0,62075 |
| ANKDD1A | -0,61754 |
| PTK2 | -0,61501 |
| ELMO3 | -0,61495 |
| LOC100049716 | -0,61471 |
| MYH3 | -0,61289 |
| DLL1 | -0,6127 |
| ODF3B | -0,61263 |
| TNFRSF9 | -0,61223 |
| LINC01004 | -0,6109 |
| SMG1P7 | -0,60983 |
| THBS3 | -0,6091 |
| CROCCP3 | -0,60705 |
| NSDHL | -0,60642 |
| STK19 | -0,60613 |
| PAX6 | -0,60461 |
| LOC100506258 | -0,60113 |
| ANKHD1 | -0,60083 |
| ALOX12P2 | -0,6007 |
| LINC01534 | -0,60057 |
| SLC2A6 | -0,60057 |
| THEMIS2 | -0,59979 |
| EMILIN2 | -0,59624 |
| LONRF1 | -0,59564 |
| L3HYPDH | -0,59401 |
| LRRC26 | -0,59276 |
| PPP1R3E | -0,59095 |
| RAD51-AS1 | -0,58957 |
| SORBS3 | -0,58939 |
| CRIP1 | -0,58718 |
| SFN | -0,58449 |
| PCYT2 | -0,58336 |
| ARRDC1 | -0,58222 |

| | |
|---|---|
| RABGGTB | -0,58114 |
| LOC101929709 | -0,58018 |
| CTTN | -0,57873 |
| FAHD2CP | -0,57857 |
| LOC728323 | -0,57816 |
| SCAMP1-AS1 | -0,57508 |
| RRAGD | -0,57482 |
| LINC00674 | -0,57101 |
| LYSMD4 | -0,57071 |
| ZNF529-AS1 | -0,56933 |
| PTOV1-AS2 | -0,56909 |
| RPL32P3 | -0,56704 |
| PPM1K | -0,56684 |
| PLEKHM1P | -0,56605 |
| COL11A2 | -0,5654 |
| DND1 | -0,56511 |
| SQLE | -0,56496 |
| WRAP73 | -0,56465 |
| BOLA1 | -0,56415 |
| FBXL15 | -0,56395 |
| C2orf76 | -0,56339 |
| NPIPA5 | -0,56313 |
| TCF7 | -0,56257 |
| LINC01160 | -0,56248 |
| PCBP1-AS1 | -0,56241 |
| NFKBIA | -0,56155 |
| RPH3AL | -0,56068 |
| TPCN2 | -0,56014 |
| LOC102606465 | -0,55911 |
| CIB1 | -0,55892 |
| MPST | -0,55825 |
| DDX26B | -0,55825 |
| PARGP1 | -0,55771 |
| DBI | -0,55716 |
| ANKRD16 | -0,55673 |
| LOC100128398 | -0,55648 |
| FAM216A | -0,55354 |
| ZGLP1 | -0,55289 |
| CEBPB | -0,55272 |
| ZFYVE19 | -0,55264 |
| LOC728613 | -0,55243 |
| LOC729737 | -0,55179 |
| MAPKAPK5-AS1 | -0,55086 |
| LOC100507006 | -0,55064 |
| MAMDC4 | -0,5505 |
| ENTPD2 | -0,55016 |

| | |
|---|---|
| SEMA4C | -0,54789 |
| TRIP10 | -0,54642 |
| DPM3 | -0,54604 |
| RPL23AP82 | -0,54495 |
| CLCF1 | -0,54448 |
| IER2 | -0,53554 |
| TRIM52 | -0,53508 |
| LTB4R2 | -0,53347 |
| MAPK11 | -0,53254 |
| STARD4 | -0,53101 |
| GTF2IP20 | -0,52954 |
| SNHG4 | -0,5253 |
| SLC26A6 | -0,52415 |
| SUZ12P1 | -0,52394 |
| MIR142 | -0,52365 |
| LOC155060 | -0,52227 |
| ABCA2 | -0,52164 |
| PDXDC2P | -0,52056 |
| SLC27A1 | -0,51812 |
| SUSD3 | -0,51713 |
| SPATA24 | -0,51702 |
| ARHGEF17 | -0,51617 |
| MMP25-AS1 | -0,51604 |
| LHX4-AS1 | -0,51542 |
| HMGCR | -0,51524 |
| CCDC120 | -0,51334 |
| RNF215 | -0,51315 |
| EHD1 | -0,51284 |
| PIK3AP1 | -0,51275 |
| TNF | -0,51153 |
| RRN3P1 | -0,51032 |
| ZFP3 | -0,5093 |
| SPSB3 | -0,50822 |
| TPT1-AS1 | -0,50821 |
| NFKB2 | -0,50727 |
| SLC50A1 | -0,50571 |
| SEC61A2 | -0,50564 |
| PIGBOS1 | -0,50532 |
| RASSF4 | -0,50491 |
| AGAP9 | -0,50445 |
| ACHE | -0,50417 |
| PIK3IP1 | -0,50391 |
| TOPORS-AS1 | -0,5014 |
| SNORA40 | -0,50108 |
| ALOX12-AS1 | -0,50102 |
| TMEM129 | -0,50045 |

| | |
|---|---|
| ZSCAN16-AS1 | -0,49951 |
| C2orf81 | -0,49892 |
| MAP3K8 | -0,49862 |
| TNFRSF14 | -0,49767 |
| SLC29A3 | -0,49731 |
| CCDC136 | -0,49703 |
| RSAD2 | -0,49683 |
| IFNGR1 | -0,49671 |
| FBXO6 | -0,49576 |
| GLUD1P3 | -0,49545 |
| MKNK2 | -0,49542 |
| AGER | -0,49333 |
| DUSP10 | -0,49105 |
| FBXL19-AS1 | -0,49083 |
| CSF1 | -0,49046 |
| PIM3 | -0,48934 |
| PHYKPL | -0,48926 |
| LRRC27 | -0,48839 |
| EPHB4 | -0,48643 |
| NPR2 | -0,4864 |
| TRPV1 | -0,48628 |
| RPL17 | -0,48419 |
| RRN3P2 | -0,48239 |
| UPB1 | -0,48192 |
| NFKBIE | -0,48187 |
| IL4R | -0,48099 |
| OVCA2 | -0,48097 |
| LRMP | -0,48003 |
| TMEM161B-AS1 | -0,47972 |
| MIR17HG | -0,47912 |
| TMEM80 | -0,47838 |
| UNC119 | -0,47592 |
| VPS9D1 | -0,47514 |
| LGALS3 | -0,4749 |
| DMPK | -0,47479 |
| S100A6 | -0,47433 |
| KCTD13 | -0,47385 |
| P2RX5 | -0,47177 |
| GPR75 | -0,47114 |
| STX1A | -0,47032 |
| LMBR1L | -0,46976 |
| ABTB2 | -0,46974 |
| ZBTB32 | -0,46944 |
| CSTB | -0,46832 |
| EMID1 | -0,46769 |
| IFI44L | -0,46747 |

| | |
|---|---|
| ZNF222 | -0,46722 |
| MAFIP | -0,46695 |
| SNHG7 | -0,46516 |
| TRADD | -0,46426 |
| CASC10 | -0,46255 |
| ATAT1 | -0,46229 |
| LINC00493 | -0,46225 |
| ATG16L2 | -0,46143 |
| CLIC4 | -0,46046 |
| SIAH2 | -0,46034 |
| PSMG3 | -0,45952 |
| ZNF593 | -0,4582 |
| ADAT2 | -0,45793 |
| ERV3-1 | -0,45708 |
| LUC7L | -0,45456 |
| RAB11FIP1 | -0,45332 |
| ID2 | -0,45292 |
| IFITM1 | -0,45223 |
| DKFZP434I0714 | -0,45176 |
| HLA-L | -0,4517 |
| HERC2P9 | -0,45063 |
| TARBP1 | -0,45062 |
| NBR2 | -0,45048 |
| C15orf40 | -0,44945 |
| SRGN | -0,44785 |
| ADAP1 | -0,44703 |
| TP53I13 | -0,44573 |
| MAP4K3 | -0,4443 |
| PMS2P3 | -0,44409 |
| IL6R | -0,4438 |
| C11orf71 | -0,44335 |
| MIR3916 | -0,44334 |
| FBXW9 | -0,44265 |
| TNFAIP3 | -0,44259 |
| DVL1 | -0,4421 |
| GRAMD1A | -0,44166 |
| MICA | -0,44103 |
| POR | -0,43983 |
| TNFRSF13C | -0,43946 |
| HOOK2 | -0,43894 |
| LYSMD2 | -0,43842 |
| MCOLN2 | -0,43841 |
| DGKQ | -0,43836 |
| COMMD3 | -0,43782 |
| GGA1 | -0,43777 |
| ARRDC1-AS1 | -0,43774 |

| | |
|---|---|
| DEXI | -0,43772 |
| KPTN | -0,43744 |
| ABCA10 | -0,43736 |
| TMEM55B | -0,43714 |
| SH3BP2 | -0,43707 |
| METTL22 | -0,43604 |
| TCTN1 | -0,43541 |
| DPP7 | -0,43345 |
| NUPL2 | -0,43266 |
| PGBD2 | -0,43192 |
| IFIT2 | -0,4312 |
| C5orf45 | -0,4302 |
| PCGF1 | -0,4301 |
| ANKZF1 | -0,42956 |
| IRX6 | -0,42722 |
| PLXNA3 | -0,42588 |
| ARID5A | -0,42519 |
| ERO1B | -0,42504 |
| PHC1 | -0,4247 |
| TESK2 | -0,42468 |
| EBI3 | -0,4238 |
| LIN37 | -0,42371 |
| PPIEL | -0,42369 |
| OSBPL7 | -0,42348 |
| HOXB3 | -0,42317 |
| PGBD4 | -0,42285 |
| TSPAN15 | -0,42281 |
| DNAAF2 | -0,42249 |
| FKBP14 | -0,42103 |
| SNHG15 | -0,41992 |
| PDE6D | -0,41928 |
| PTRH2 | -0,4182 |
| FAM73B | -0,41737 |
| GRHPR | -0,41722 |
| CD58 | -0,41675 |
| PLD2 | -0,41674 |
| ZC3H12A | -0,41649 |
| WASH7P | -0,41638 |
| C19orf66 | -0,41596 |
| ANKRD23 | -0,41591 |
| VIMP | -0,4148 |
| CD46 | -0,41464 |
| NECAB3 | -0,41251 |
| NSMF | -0,41196 |
| EBP | -0,41149 |
| LINC-PINT | -0,41094 |

| | |
|---|---|
| ELP5 | -0,41087 |
| DHRS1 | -0,41082 |
| NPRL2 | -0,41068 |
| PPP1R12C | -0,41053 |
| CTSZ | -0,41016 |
| NAT14 | -0,40799 |
| PLXNB1 | -0,40751 |
| RSAD1 | -0,40742 |
| LOC441242 | -0,40736 |
| C9orf72 | -0,40732 |
| ATP6AP1L | -0,40359 |
| VAMP5 | -0,40336 |
| ZNF446 | -0,4031 |
| C21orf91 | -0,40278 |
| RFNG | -0,40249 |
| ARHGAP33 | -0,40241 |
| BISPR | -0,40228 |
| TMSB10 | -0,40202 |
| PSMG4 | -0,40187 |
| RTN2 | -0,40141 |
| TFAP2A | -0,401 |
| OSGEPL1 | -0,4009 |
| GUSBP1 | -0,40027 |
| SYNGAP1 | -0,40009 |
| DPY19L2P2 | -0,3999 |
| SS18L2 | -0,39963 |
| ABCA7 | -0,39699 |
| DENND3 | -0,395 |
| LZTR1 | -0,39425 |
| KLC4 | -0,39415 |
| CDKN2A | -0,39336 |
| DYNC2LI1 | -0,39315 |
| INAFM2 | -0,39197 |
| COA5 | -0,39178 |
| FAM173A | -0,39139 |
| SS18L1 | -0,39116 |
| NAT9 | -0,39043 |
| ALPK1 | -0,38891 |
| HNRNPU-AS1 | -0,38863 |
| CYTH2 | -0,38861 |
| YPEL3 | -0,38846 |
| DUS4L | -0,3876 |
| FASTKD3 | -0,38666 |
| UAP1L1 | -0,38598 |
| SNHG5 | -0,38545 |
| TRIB3 | -0,38505 |

| | |
|---|---|
| KDM5D | -0,38392 |
| RRAGB | -0,38321 |
| DUSP11 | -0,38142 |
| TMEM243 | -0,38071 |
| ZNF839 | -0,38051 |
| ISG20 | -0,37991 |
| EIF1 | -0,37987 |
| KIAA1407 | -0,37922 |
| BTN2A2 | -0,3788 |
| VMAC | -0,37865 |
| CCDC92 | -0,37848 |
| TMEM63B | -0,37732 |
| HDHD3 | -0,37716 |
| MIF-AS1 | -0,37683 |
| TSNARE1 | -0,37588 |
| LOC440434 | -0,37585 |
| FAM60A | -0,37562 |
| ECHDC2 | -0,37552 |
| WDR19 | -0,37526 |
| COQ10A | -0,37498 |
| MED7 | -0,37484 |
| LINS | -0,37472 |
| SUPT7L | -0,37427 |
| PPP4R1L | -0,37411 |
| FAM89B | -0,37399 |
| NR2C1 | -0,37371 |
| RRAS | -0,37333 |
| STAG3L4 | -0,37281 |
| NFKBID | -0,37235 |
| CALCOCO1 | -0,37019 |
| TAGLN | -0,36962 |
| WASH1 | -0,36938 |
| ERMARD | -0,36858 |
| HTRA2 | -0,36822 |
| LOC100507195 | -0,3677 |
| BSDC1 | -0,36668 |
| CCNG2 | -0,36564 |
| QPCTL | -0,36554 |
| ACTR1B | -0,36532 |
| PKD1P6 | -0,36484 |
| FASTK | -0,36462 |
| HSH2D | -0,3645 |
| TMEM216 | -0,36398 |
| FADS3 | -0,36385 |
| TAZ | -0,36339 |
| C6orf1 | -0,36259 |

| | |
|---|---|
| GPR137B | -0,36222 |
| NUMBL | -0,36163 |
| CLK2 | -0,36149 |
| P4HTM | -0,3611 |
| USP18 | -0,36026 |
| DHX58 | -0,36026 |
| PCMTD2 | -0,35915 |
| FLYWCH2 | -0,35909 |
| EZH1 | -0,35817 |
| ELMOD3 | -0,35771 |
| NCBP2-AS2 | -0,35753 |
| IFT140 | -0,35742 |
| FAM195B | -0,35676 |
| MAPKBP1 | -0,35672 |
| TRIM41 | -0,35529 |
| ENDOG | -0,35526 |
| DCUN1D2 | -0,35486 |
| XPNPEP1 | -0,35455 |
| ORMDL1 | -0,35422 |
| TMEM175 | -0,35402 |
| HIST1H2AC | -0,35372 |
| LRP8 | -0,35361 |
| SLC25A37 | -0,35319 |
| CD22 | -0,35275 |
| TYMP | -0,35248 |
| SFI1 | -0,35246 |
| LAT2 | -0,35168 |
| SH3YL1 | -0,35162 |
| P2RX4 | -0,34995 |
| AFMID | -0,34984 |
| ZDHHC8 | -0,34892 |
| FLOT1 | -0,34878 |
| POP5 | -0,34826 |
| N4BP2L2 | -0,34773 |
| ago-04 | -0,34734 |
| CYP4V2 | -0,34697 |
| PPAPDC1B | -0,34627 |
| SLC12A9 | -0,34624 |
| WDR5B | -0,34576 |
| PEX16 | -0,34533 |
| DDX5 | -0,34518 |
| NADSYN1 | -0,34495 |
| REC8 | -0,34479 |
| MAT2A | -0,34468 |
| STARD3NL | -0,34431 |
| FBXO44 | -0,34428 |

| | |
|---|---|
| HSBP1 | -0,34398 |
| C9orf142 | -0,34396 |
| GALE | -0,34393 |
| ZFC3H1 | -0,34336 |
| CD80 | -0,34228 |
| CYB5A | -0,34145 |
| STAT6 | -0,34107 |
| NOP10 | -0,34103 |
| NUDT16L1 | -0,34076 |
| RANGRF | -0,34055 |
| LINC01215 | -0,33959 |
| BCS1L | -0,33916 |
| GBA2 | -0,33906 |
| CD274 | -0,33881 |
| HMG20B | -0,33809 |
| LHX2 | -0,3379 |
| POLM | -0,33783 |
| UTP6 | -0,33727 |
| PDCD5 | -0,33719 |
| PGS1 | -0,33716 |
| LINC00623 | -0,33697 |
| FANCL | -0,33675 |
| TJAP1 | -0,33635 |
| OFD1 | -0,33593 |
| ACSF2 | -0,33573 |
| ZNRD1 | -0,33555 |
| CCDC142 | -0,33545 |
| PAX8-AS1 | -0,33533 |
| COX10-AS1 | -0,33453 |
| RPL22L1 | -0,33412 |
| TRMT10B | -0,33341 |
| FAHD2A | -0,33226 |
| UBL5 | -0,33224 |
| CCDC85B | -0,33071 |
| RPL39 | -0,33054 |
| ANKRD13D | -0,33035 |
| LRSAM1 | -0,33017 |
| ZNF75D | -0,33016 |
| OXLD1 | -0,32945 |
| ZNF251 | -0,32936 |
| LAMTOR2 | -0,32899 |
| ACAP3 | -0,32883 |
| C19orf60 | -0,32687 |
| KAT2A | -0,32684 |
| ZNF581 | -0,32653 |
| WASH3P | -0,32646 |

| | |
|---|---|
| SLAMF1 | -0,32595 |
| RBM48 | -0,32593 |
| PTS | -0,32559 |
| MAP3K10 | -0,32454 |
| MARCH9 | -0,32378 |
| ZFAS1 | -0,3236 |
| TIMM23B | -0,32341 |
| RNF170 | -0,32273 |
| ACYP1 | -0,32251 |
| SGTB | -0,32166 |
| RAB40C | -0,32122 |
| CHMP5 | -0,32114 |
| ZBTB26 | -0,32092 |
| NBPF8 | -0,32008 |
| IKBKE | -0,31991 |
| WDR91 | -0,31972 |
| ARFIP2 | -0,31955 |
| ARNTL | -0,31842 |
| NR6A1 | -0,31822 |
| RRN3P3 | -0,31819 |
| MPG | -0,31748 |
| CCDC159 | -0,3171 |
| ANKRD10 | -0,31689 |
| CBWD5 | -0,31657 |
| CCDC14 | -0,31637 |
| NBPF11 | -0,31637 |
| IP6K2 | -0,31527 |
| PSMA3-AS1 | -0,31504 |
| UGCG | -0,31492 |
| N4BP2L1 | -0,31434 |
| PI4KAP2 | -0,31411 |
| TUBE1 | -0,31402 |
| EMC6 | -0,31375 |
| DALRD3 | -0,31348 |
| PARP6 | -0,31336 |
| NIT1 | -0,31321 |
| SELO | -0,31311 |
| TRAF4 | -0,31277 |
| BTAF1 | -0,31275 |
| TRMT61B | -0,3126 |
| C11orf49 | -0,31259 |
| WDR45 | -0,31211 |
| MRPL14 | -0,31095 |
| BTN2A1 | -0,31089 |
| RPL36 | -0,31058 |
| PLSCR1 | -0,3098 |

| | |
|---|---|
| PLEKHF2 | -0,30947 |
| MRPL33 | -0,30942 |
| MXD4 | -0,30867 |
| NEK8 | -0,30862 |
| CHPT1 | -0,3086 |
| DIMT1 | -0,30848 |
| MPLKIP | -0,30797 |
| TBCK | -0,30502 |
| ALKBH6 | -0,30473 |
| ZNF83 | -0,30343 |
| FBXO9 | -0,3028 |
| GEMIN7 | -0,30257 |
| IFI44 | -0,30185 |
| LINC00909 | -0,30181 |
| ARL16 | -0,30179 |
| QTRT1 | -0,30161 |
| MAGEF1 | -0,3016 |
| CCDC84 | -0,3015 |
| USMG5 | -0,30112 |
| FCHSD1 | -0,30094 |
| TMEM43 | -0,30082 |
| ALKBH7 | -0,30066 |
| C17orf75 | -0,30021 |
| ABCC10 | -0,30002 |
| RHOQ | -0,30002 |
| C21orf59 | -0,29898 |
| AKTIP | -0,29874 |
| SNRPD2 | -0,29841 |
| ISG15 | -0,29716 |
| DNAJC19 | -0,29654 |
| SRSF5 | -0,29634 |
| ANAPC10 | -0,29561 |
| AP4M1 | -0,29531 |
| RPL35 | -0,29522 |
| TIMM8B | -0,29485 |
| CRYBB2P1 | -0,29457 |
| MRPS21 | -0,2944 |
| MICAL1 | -0,29393 |
| RIC8B | -0,2937 |
| RRP7BP | -0,2936 |
| RBX1 | -0,29329 |
| MKS1 | -0,2929 |
| MED30 | -0,29283 |
| RAB11FIP3 | -0,29281 |
| ZNF226 | -0,29199 |
| CLSTN3 | -0,29144 |

| | |
|---|---|
| APBA3 | -0,29108 |
| FNBP4 | -0,29088 |
| GSKIP | -0,29054 |
| C16orf13 | -0,29048 |
| CDK7 | -0,29048 |
| DFFB | -0,29017 |
| C21orf33 | -0,28979 |
| SPATA20 | -0,28964 |
| GSTK1 | -0,2892 |
| TMEM134 | -0,28897 |
| TMEM161A | -0,28873 |
| ASB16-AS1 | -0,2887 |
| RBCK1 | -0,28862 |
| ARV1 | -0,28856 |
| ZNF428 | -0,28845 |
| LINC00094 | -0,28831 |
| BBS2 | -0,28812 |
| SAT2 | -0,28672 |
| DNAJB9 | -0,28596 |
| APIP | -0,28578 |
| TRABD | -0,28577 |
| AFG3L1P | -0,28515 |
| AKR1A1 | -0,28496 |
| PPP3CC | -0,28483 |
| IKBKB | -0,28432 |
| ANKLE1 | -0,28419 |
| AUP1 | -0,28414 |
| ATP6V1F | -0,28401 |
| ZNF639 | -0,28393 |
| GABARAPL2 | -0,28362 |
| DUSP12 | -0,2836 |
| PRKAB2 | -0,2834 |
| VCPKMT | -0,28333 |
| ZNF558 | -0,28293 |
| UBE2B | -0,28288 |
| MYO19 | -0,28261 |
| TMEM205 | -0,28257 |
| IRF7 | -0,28182 |
| TSPAN31 | -0,28084 |
| ORAOV1 | -0,28002 |
| ATP8B2 | -0,27966 |
| CREBZF | -0,27917 |
| UBXN11 | -0,27881 |
| ZNF783 | -0,27858 |
| C1orf27 | -0,2785 |
| DTX3 | -0,27811 |

| | |
|---|---|
| WDR59 | -0,27749 |
| POLR2L | -0,2772 |
| TMEM161B | -0,27717 |
| SNAPC4 | -0,27672 |
| PIGQ | -0,27619 |
| HIP1R | -0,27595 |
| CSNK1G2 | -0,27533 |
| QSOX2 | -0,27491 |
| E4F1 | -0,2743 |
| ATP5J2 | -0,27414 |
| C8orf59 | -0,27399 |
| GAS5 | -0,27388 |
| PTRHD1 | -0,27374 |
| CAPRIN2 | -0,27373 |
| NIPAL2 | -0,27306 |
| DCAKD | -0,27276 |
| AKAP17A | -0,2726 |
| PPCS | -0,27253 |
| TRMT2A | -0,27184 |
| TRAF5 | -0,27166 |
| TRMU | -0,27071 |
| TOP1MT | -0,27032 |
| BIN2 | -0,26995 |
| GTPBP2 | -0,26901 |
| C9orf16 | -0,26893 |
| ADAM8 | -0,26796 |
| ZNF500 | -0,26753 |
| FAM58A | -0,267 |
| LINC00938 | -0,26698 |
| ZNF589 | -0,2668 |
| SDCCAG3 | -0,26661 |
| STX2 | -0,26607 |
| DHPS | -0,26606 |
| C2orf47 | -0,26591 |
| IL17RB | -0,26536 |
| DDX17 | -0,26493 |
| SPG7 | -0,26471 |
| DXO | -0,26466 |
| NXF1 | -0,26436 |
| IRF9 | -0,26426 |
| POLL | -0,2641 |
| ISL2 | -0,26372 |
| UCKL1 | -0,2636 |
| MUS81 | -0,26342 |
| C19orf25 | -0,26312 |
| MRPL54 | -0,26293 |

| | |
|---|---|
| GFOD2 | -0,26272 |
| ADCK4 | -0,26255 |
| ARHGEF1 | -0,26251 |
| NSUN5 | -0,26244 |
| OAZ2 | -0,26115 |
| HOXB4 | -0,26054 |
| LARP1B | -0,26027 |
| PAN2 | -0,25996 |
| TBRG1 | -0,25929 |
| CASZ1 | -0,25913 |
| SDCBP | -0,2584 |
| C19orf24 | -0,25838 |
| ANKRD49 | -0,25701 |
| SLC25A28 | -0,25636 |
| MIEN1 | -0,25584 |
| P3H1 | -0,2555 |
| NT5C | -0,2551 |
| UGGT2 | -0,2549 |
| DCAF8 | -0,25486 |
| TRIT1 | -0,25436 |
| MOAP1 | -0,25405 |
| TAF10 | -0,25373 |
| NPHP4 | -0,25346 |
| RPL29 | -0,25285 |
| TBCC | -0,2528 |
| TMEM120A | -0,2525 |
| PSMB8-AS1 | -0,25247 |
| USP45 | -0,25239 |
| CBWD2 | -0,25237 |
| LTBP4 | -0,25227 |
| UNC50 | -0,25099 |
| JRKL | -0,25086 |
| MPV17 | -0,25045 |
| IRF3 | -0,24985 |
| RPL38 | -0,24943 |
| ZNF600 | -0,24927 |
| SIN3B | -0,24892 |
| RPGRIP1L | -0,24853 |
| EXOSC5 | -0,24837 |
| LRRC14 | -0,24806 |
| CCDC66 | -0,24771 |
| RBM5 | -0,24743 |
| CHMP1B | -0,24718 |
| C5orf24 | -0,24683 |
| MIB2 | -0,24674 |
| PIKFYVE | -0,2465 |

| | |
|---|---|
| ZNF655 | -0,24572 |
| PRPSAP2 | -0,24562 |
| CCDC88B | -0,24523 |
| KRCC1 | -0,24514 |
| POMGNT1 | -0,24502 |
| KIAA0141 | -0,24477 |
| THOP1 | -0,24456 |
| OCIAD2 | -0,24442 |
| CNTNAP1 | -0,24393 |
| RPS18 | -0,24392 |
| ALG13 | -0,2438 |
| AP1G2 | -0,24374 |
| MTG1 | -0,24337 |
| RPL31 | -0,24262 |
| LYPLAL1 | -0,24205 |
| ZNF580 | -0,24167 |
| CCNDBP1 | -0,24154 |
| TBCB | -0,24153 |
| FKBP3 | -0,24149 |
| RBM6 | -0,24114 |
| ZBED5 | -0,24098 |
| IFFO1 | -0,24057 |
| TAF11 | -0,24023 |
| GLT8D1 | -0,24017 |
| LSM7 | -0,23909 |
| KATNB1 | -0,23903 |
| PLEKHJ1 | -0,23888 |
| SSSCA1 | -0,23855 |
| NXT1 | -0,23847 |
| POLR2I | -0,23842 |
| SLC25A29 | -0,23811 |
| EBLN3 | -0,23805 |
| XAF1 | -0,238 |
| SH3GLB2 | -0,23752 |
| COMMD6 | -0,23739 |
| PNPLA2 | -0,23661 |
| ILKAP | -0,23659 |
| RAB11FIP2 | -0,23628 |
| RAB24 | -0,23597 |
| FLAD1 | -0,23592 |
| POLD4 | -0,23589 |
| CD48 | -0,23531 |
| NECAP1 | -0,23524 |
| TOP3B | -0,2342 |
| ATAD3B | -0,23419 |
| ZRANB2 | -0,23281 |

| | |
|---|---|
| STK25 | -0,23233 |
| GRAP | -0,23214 |
| GTPBP3 | -0,23206 |
| RPL32 | -0,23198 |
| RPL18A | -0,23191 |
| C19orf53 | -0,2315 |
| ZNHIT1 | -0,23125 |
| VPS28 | -0,23111 |
| NUDT22 | -0,23074 |
| PAXBP1 | -0,23069 |
| PFDN5 | -0,2293 |
| ZNF76 | -0,22919 |
| ZNF830 | -0,22879 |
| ZNF512B | -0,22853 |
| CHCHD7 | -0,22784 |
| RCCD1 | -0,22763 |
| DMAP1 | -0,22696 |
| CLCN7 | -0,22679 |
| DGUOK | -0,22654 |
| PNKP | -0,22598 |
| IQCB1 | -0,22562 |
| DHX34 | -0,22538 |
| TOMM6 | -0,22533 |
| ACTR10 | -0,22507 |
| NDUFAF7 | -0,2246 |
| INTS3 | -0,22439 |
| IFI6 | -0,22422 |
| ULK1 | -0,22371 |
| RXRB | -0,2237 |
| TNFRSF10B | -0,22353 |
| MRPL43 | -0,22349 |
| TXNDC9 | -0,2233 |
| PLK3 | -0,22327 |
| MRPS25 | -0,22237 |
| SNX22 | -0,22148 |
| RNF19A | -0,22122 |
| NGRN | -0,22107 |
| RPL37 | -0,22082 |
| SNHG16 | -0,22021 |
| VMP1 | -0,21982 |
| ATP6V1E1 | -0,21944 |
| ACAA1 | -0,21914 |
| CISD2 | -0,21815 |
| PRPF39 | -0,21799 |
| GNL3 | -0,21722 |
| MTERF3 | -0,21711 |

| | |
|---|---|
| CHFR | -0,21687 |
| CD70 | -0,21568 |
| MAP3K13 | -0,21528 |
| C19orf48 | -0,21498 |
| RPL41 | -0,21442 |
| ABCC5 | -0,21439 |
| SHARPIN | -0,21265 |
| STX8 | -0,21252 |
| GPR108 | -0,21201 |
| TAF7 | -0,20999 |
| COPS7B | -0,20942 |
| CD19 | -0,20911 |
| DRG2 | -0,20542 |
| ATG4B | -0,20529 |
| COX6B1 | -0,20454 |
| RPL12 | -0,20433 |
| PHF11 | -0,20408 |
| HAX1 | -0,20222 |
| CPNE1 | -0,20155 |
| LINC00667 | -0,20056 |
| COX7A2 | -0,20038 |
| ATG16L1 | -0,20004 |
| OAZ1 | -0,19943 |
| RPS21 | -0,19919 |
| WDR11 | -0,19849 |
| EMC4 | -0,19717 |
| PPIA | -0,19706 |
| IGSF8 | -0,19573 |
| JAK3 | -0,19485 |
| TKFC | -0,19457 |
| RNF126 | -0,19455 |
| ZBTB40 | -0,19424 |
| NDUFB7 | -0,19386 |
| EXOSC9 | -0,19298 |
| PPP1R21 | -0,19115 |
| WDR74 | -0,19044 |
| CHD1L | -0,18931 |
| DYNLL1 | -0,18841 |
| MRPL38 | -0,18786 |
| RBM39 | -0,18772 |
| MTERF4 | -0,18644 |
| THOC1 | -0,18412 |
| ARHGAP4 | -0,18309 |
| PPP6R2 | -0,18147 |
| C7orf73 | -0,18094 |
| LIG4 | -0,18087 |

| | |
|---|---|
| RELT | -0,18033 |
| PSMB10 | -0,1796 |
| ERCC3 | -0,1783 |
| GSDMD | -0,17448 |
| RPS27 | -0,17394 |
| TRIM22 | -0,17367 |
| MCRS1 | -0,17358 |
| LIMD2 | -0,17315 |
| ZC3H11A | -0,17314 |
| SRSF11 | -0,1718 |
| MFF | -0,16973 |
| LILRB1 | -0,16815 |
| EML3 | -0,16317 |
| COX7B | -0,15908 |
| PPP5C | -0,15824 |

Up-regulated

| Gene | log2FoldChange |
|---|---|
| LARP1 | 0,138036716 |
| WASF2 | 0,139405955 |
| KPNB1 | 0,139409952 |
| SNX29 | 0,139845894 |
| API5 | 0,140742285 |
| KLF13 | 0,142972624 |
| KHDRBS1 | 0,144399429 |
| SUZ12 | 0,145034067 |
| SEPT11 | 0,146677341 |
| CAD | 0,148254218 |
| SETD1A | 0,149252754 |
| WDR82 | 0,149348754 |
| KCTD20 | 0,150359206 |
| ATP2A2 | 0,150495085 |
| LONP1 | 0,152143157 |
| IPO5 | 0,152596706 |
| RRM2B | 0,152615555 |
| GTF3C1 | 0,153206703 |
| KRR1 | 0,153703361 |
| SEC63 | 0,155025741 |
| SMCR8 | 0,155616674 |
| GALNT2 | 0,157172778 |
| DUSP4 | 0,158361605 |
| STK10 | 0,15893962 |
| GATAD2A | 0,159934103 |
| CUL3 | 0,160324022 |

| | |
|---|---|
| WDFY4 | 0,160327658 |
| NUP153 | 0,160816747 |
| SLC38A1 | 0,161050589 |
| SHOC2 | 0,161258776 |
| IBTK | 0,161333958 |
| CRTC3 | 0,161771369 |
| CNTRL | 0,162022186 |
| ICMT | 0,163499937 |
| SYNCRIP | 0,16365999 |
| ACTR2 | 0,164058351 |
| SUPT16H | 0,164200637 |
| CDK12 | 0,164479029 |
| RAVER1 | 0,164944257 |
| PAK2 | 0,16533603 |
| TNPO3 | 0,165660853 |
| USP9X | 0,165666411 |
| YWHAH | 0,165720796 |
| HDGF | 0,165844565 |
| BTBD10 | 0,166003314 |
| MICU1 | 0,166111511 |
| CREBBP | 0,166119035 |
| LSP1 | 0,166168736 |
| BMPR2 | 0,166434936 |
| SLC7A1 | 0,166501618 |
| CHST11 | 0,167170275 |
| ATF7 | 0,167902059 |
| NOM1 | 0,168287705 |
| ELF4 | 0,16916826 |
| PRRC1 | 0,169503608 |
| SLC16A1 | 0,169800209 |
| TRIP12 | 0,169907692 |
| MTHFD1 | 0,170139269 |
| TFDP2 | 0,1701595 |
| ARHGEF12 | 0,170626981 |
| BCL9L | 0,170665499 |
| MED16 | 0,170704694 |
| FAF2 | 0,17100597 |
| ANAPC1 | 0,171245534 |
| PREX1 | 0,171339223 |
| APC | 0,171504555 |
| SAP130 | 0,172592683 |
| G3BP2 | 0,172742182 |
| RNF219 | 0,17339556 |
| MTA2 | 0,174316658 |
| USP7 | 0,174738793 |
| B4GALT1 | 0,175403543 |

| | |
|---|---|
| CLTC | 0,175647503 |
| CAND1 | 0,175657181 |
| MED13 | 0,176360438 |
| PDE4B | 0,176722936 |
| STAG2 | 0,176826588 |
| TNRC18 | 0,176831488 |
| PTPN7 | 0,177077841 |
| SF3B3 | 0,177512935 |
| HSPD1 | 0,177598236 |
| PRRC2B | 0,178138615 |
| AARS | 0,178283752 |
| GTF3C4 | 0,178354844 |
| C15orf39 | 0,178446135 |
| KIAA0100 | 0,1795532 |
| GIGYF2 | 0,179970099 |
| CDK6 | 0,180130957 |
| RAB3GAP1 | 0,18081978 |
| CADM1 | 0,181026711 |
| POM121 | 0,181215273 |
| UBTF | 0,18177924 |
| ANKRD52 | 0,182255009 |
| GOT2 | 0,183109066 |
| STAU1 | 0,183293608 |
| GPR107 | 0,183695288 |
| WIPF2 | 0,183695392 |
| PJA2 | 0,184561131 |
| NUCB1 | 0,185149863 |
| PIK3R1 | 0,185445943 |
| PVRL1 | 0,185793927 |
| GPI | 0,186070126 |
| PTBP3 | 0,186621436 |
| SSH2 | 0,186916819 |
| POLR2A | 0,187045404 |
| SH3KBP1 | 0,187305959 |
| LAPTM4A | 0,189226775 |
| SPTLC2 | 0,18923193 |
| PDK3 | 0,189251902 |
| ORAI2 | 0,189838958 |
| ZAK | 0,190242973 |
| RPRD2 | 0,190991213 |
| QSER1 | 0,191035826 |
| TRIP11 | 0,19105732 |
| FOCAD | 0,191086285 |
| LMAN2 | 0,191223837 |
| NUP188 | 0,191564216 |
| RANBP2 | 0,192874619 |

| | |
|---|---|
| TAF4 | 0,193779477 |
| KIF1B | 0,193826714 |
| CACYBP | 0,19400155 |
| ZNF766 | 0,194290028 |
| IQGAP2 | 0,194326685 |
| SUPT6H | 0,194344558 |
| RAPGEF6 | 0,194489913 |
| SON | 0,194701539 |
| HIRA | 0,195593957 |
| CAMSAP2 | 0,195802315 |
| NUDT21 | 0,196389178 |
| STAG1 | 0,196600654 |
| SF3B4 | 0,196803485 |
| RAB3D | 0,197569589 |
| IQSEC1 | 0,197658382 |
| CYB5R3 | 0,197896156 |
| TBC1D5 | 0,19792294 |
| NCOR2 | 0,198100206 |
| CHD8 | 0,198115891 |
| LDHA | 0,198200505 |
| SBNO1 | 0,1984543 |
| GBF1 | 0,19851371 |
| SPTAN1 | 0,198636299 |
| SLC9A3R1 | 0,198745723 |
| WBSCR16 | 0,198868812 |
| EIF4EBP2 | 0,198904092 |
| LRRC58 | 0,199548638 |
| RERE | 0,199672074 |
| ARHGAP1 | 0,199806231 |
| KDM3B | 0,200033493 |
| SERTAD2 | 0,20037047 |
| POLR1A | 0,200756354 |
| ZYX | 0,201874429 |
| NXPE3 | 0,20222112 |
| SLC7A11 | 0,202985238 |
| ANKRD17 | 0,202989956 |
| SENP1 | 0,203055058 |
| GPATCH8 | 0,203734668 |
| RIF1 | 0,204620309 |
| ZFAT | 0,204703593 |
| CLPB | 0,205004021 |
| HEG1 | 0,205203085 |
| KDM5A | 0,205209563 |
| PIGN | 0,205729885 |
| TRAF3IP2 | 0,206318393 |
| PER2 | 0,206481828 |

| | |
|---|---|
| TRIM24 | 0,206545401 |
| MAP3K1 | 0,206829439 |
| SMG6 | 0,20700636 |
| AMER1 | 0,207072033 |
| SLK | 0,207259803 |
| PGM1 | 0,207436524 |
| SP1 | 0,207444092 |
| RFWD3 | 0,208036731 |
| ZC3H4 | 0,208948918 |
| RBM27 | 0,208994025 |
| DESI2 | 0,209010835 |
| SMG7 | 0,209036891 |
| GNS | 0,209075412 |
| SNX10 | 0,209285658 |
| MIB1 | 0,209459128 |
| RBPJ | 0,209545216 |
| ARRB1 | 0,209785854 |
| TNIK | 0,209794775 |
| MBNL1 | 0,209868025 |
| SP3 | 0,210118514 |
| SELL | 0,210629937 |
| CREB1 | 0,210655063 |
| CELF2 | 0,210787719 |
| PPT1 | 0,210890353 |
| TAF15 | 0,211090938 |
| UHRF1BP1 | 0,211166847 |
| IPO8 | 0,211404874 |
| ARID1A | 0,211536015 |
| C1orf216 | 0,211558842 |
| LIMS1 | 0,211590611 |
| MARCH8 | 0,211693199 |
| AKAP13 | 0,212671741 |
| ago-01 | 0,214073074 |
| RPN2 | 0,214133632 |
| GEMIN4 | 0,214333004 |
| HELZ | 0,214498879 |
| TRIM32 | 0,214917388 |
| NRF1 | 0,215932518 |
| NAV2 | 0,217239011 |
| KIAA0586 | 0,217930965 |
| H2AFZ | 0,218283209 |
| CSE1L | 0,218362092 |
| HSP90AA1 | 0,218703702 |
| PBRM1 | 0,218724378 |
| UTP20 | 0,219194557 |
| PARP1 | 0,220303781 |

| | |
|---|---|
| PEX5L | 0,220949601 |
| KIF21B | 0,221685537 |
| ARID2 | 0,22230467 |
| MYH9 | 0,222525209 |
| NR3C1 | 0,222977018 |
| PARP4 | 0,224505453 |
| HNRNPM | 0,224770672 |
| TUBB | 0,225226615 |
| MBTPS2 | 0,225293632 |
| ZMAT3 | 0,225388369 |
| ENC1 | 0,225601756 |
| ZBED1 | 0,225747727 |
| ALDH5A1 | 0,225926295 |
| CEP55 | 0,225935214 |
| CREB3L2 | 0,226577323 |
| MED1 | 0,226984669 |
| POM121C | 0,227180873 |
| BRI3BP | 0,227788607 |
| ago-02 | 0,227855139 |
| SMARCC1 | 0,228299631 |
| CCNK | 0,228713592 |
| DIAPH1 | 0,228946206 |
| FAM129A | 0,229206698 |
| BCOR | 0,229387379 |
| HTATSF1 | 0,229473758 |
| F8A1 | 0,229715582 |
| TIMP1 | 0,229826575 |
| UBR4 | 0,23016631 |
| TMEM41A | 0,230433924 |
| MECP2 | 0,230485723 |
| NF1 | 0,230635719 |
| KIAA0040 | 0,230679925 |
| SEC62 | 0,230688878 |
| TACC1 | 0,230782 |
| INO80D | 0,230795364 |
| TLN1 | 0,231293533 |
| SPEN | 0,231313932 |
| CDS2 | 0,231465712 |
| IKZF3 | 0,232143002 |
| TOR1AIP1 | 0,232726674 |
| TRIM56 | 0,232754224 |
| GMPS | 0,233449231 |
| CTDSPL2 | 0,233507838 |
| IL17RA | 0,233968579 |
| CPOX | 0,234424376 |
| KMT2E | 0,234844752 |

| | |
|---|---|
| PHGDH | 0,234854451 |
| CNOT1 | 0,235204161 |
| MFSD6 | 0,235680036 |
| C19orf47 | 0,235757818 |
| NCOR1 | 0,235933576 |
| ZNF28 | 0,23624676 |
| NDST1 | 0,236580403 |
| EP300 | 0,236669037 |
| IQGAP1 | 0,236822266 |
| HNRNPU | 0,23687303 |
| PRRC2C | 0,238269821 |
| HNRNPUL1 | 0,238830389 |
| TTYH3 | 0,238905078 |
| ALKBH8 | 0,23903266 |
| GDE1 | 0,239644322 |
| FAM168A | 0,23977653 |
| TNRC6C | 0,241532394 |
| SLC30A6 | 0,24170496 |
| ZNF609 | 0,242196011 |
| GOLIM4 | 0,242203628 |
| CLP1 | 0,242683096 |
| PIGK | 0,243314587 |
| ADCY1 | 0,243342789 |
| PDZD8 | 0,243375713 |
| RNF157 | 0,24358185 |
| CNKSR3 | 0,243599282 |
| TMEM245 | 0,244396157 |
| ZNF124 | 0,244444954 |
| LRR1 | 0,245264374 |
| GVINP1 | 0,245825708 |
| ACAT1 | 0,246139133 |
| PLD1 | 0,246504518 |
| ESYT1 | 0,246996033 |
| USP37 | 0,24788872 |
| FECH | 0,248135184 |
| IFNAR1 | 0,248371157 |
| CRTAP | 0,248924967 |
| PLK1 | 0,249016992 |
| SRBD1 | 0,249131981 |
| DCLRE1B | 0,249294906 |
| CPD | 0,249766411 |
| SETX | 0,25036826 |
| PTCH1 | 0,250871135 |
| PRR12 | 0,251002473 |
| CHAF1B | 0,251224771 |
| MACC1 | 0,252463464 |

| | |
|---|---|
| IGF2R | 0,252592259 |
| MLH1 | 0,252918919 |
| NDC1 | 0,253826607 |
| DOCK8 | 0,254464153 |
| KCTD1 | 0,254550598 |
| CBL | 0,255209506 |
| TMEM201 | 0,255384724 |
| CHD9 | 0,255585672 |
| YLPM1 | 0,256563518 |
| POLA1 | 0,257027013 |
| GUCY1A3 | 0,257147682 |
| HMMR | 0,257366447 |
| PRPF8 | 0,257653355 |
| GPD2 | 0,257888511 |
| DIP2B | 0,258129316 |
| FAM13B | 0,258423205 |
| SEC16A | 0,258615361 |
| NIPBL | 0,259090589 |
| ARHGEF6 | 0,259193099 |
| PIK3R3 | 0,259203734 |
| SACS | 0,259990635 |
| NUP210 | 0,261084054 |
| NBAS | 0,261629828 |
| MYO18A | 0,261714159 |
| LMNB1 | 0,262970609 |
| PPDPF | 0,265559348 |
| KIAA1671 | 0,265790098 |
| NUP155 | 0,266193001 |
| WDR7 | 0,266206642 |
| MEF2D | 0,266553647 |
| GTF2A1 | 0,266759 |
| SMAD5 | 0,267105242 |
| NCAPD2 | 0,267111136 |
| POLD3 | 0,267229132 |
| MPP6 | 0,268811183 |
| TTC39C | 0,268985507 |
| MSN | 0,269058295 |
| C10orf54 | 0,26979358 |
| PLP2 | 0,270327123 |
| EI24 | 0,270412873 |
| HMGN5 | 0,270591422 |
| TXNRD1 | 0,270987166 |
| UHRF1 | 0,271038374 |
| WNK1 | 0,271815161 |
| NOMO2 | 0,27275246 |
| CHAMP1 | 0,274126389 |

| | |
|---|---|
| LRBA | 0,274331611 |
| IRS1 | 0,275044471 |
| MAP3K9 | 0,275058057 |
| APOBEC3B | 0,275885616 |
| MGA | 0,275996762 |
| LIMA1 | 0,27640792 |
| IPO11 | 0,276734671 |
| TUBB6 | 0,277116269 |
| MCM6 | 0,277134295 |
| NFIC | 0,277157362 |
| MAD2L1 | 0,277261598 |
| LASP1 | 0,2775517 |
| RAD54L2 | 0,278624398 |
| SLC1A4 | 0,27890272 |
| COPG2 | 0,279303168 |
| CALM3 | 0,279683731 |
| DSG2 | 0,280008951 |
| ZNF407 | 0,280177619 |
| IKZF2 | 0,281356935 |
| SRCAP | 0,281390844 |
| NEDD4 | 0,282238177 |
| EVI2B | 0,282259236 |
| MACF1 | 0,28292974 |
| CALU | 0,283883282 |
| ANP32E | 0,284624139 |
| NCOA2 | 0,285171103 |
| TMX4 | 0,287033973 |
| BAZ1B | 0,287707965 |
| MYCBP2 | 0,287893891 |
| ARHGAP35 | 0,289295967 |
| CABLES2 | 0,289586133 |
| NET1 | 0,290512023 |
| HPSE | 0,291368405 |
| HIPK2 | 0,29166724 |
| HNRNPD | 0,292183882 |
| ZNF675 | 0,292316246 |
| ARHGAP26 | 0,292747372 |
| KATNAL1 | 0,294557569 |
| SLC25A10 | 0,295000442 |
| USP13 | 0,29520992 |
| TNRC6B | 0,295342475 |
| NTRK2 | 0,296670417 |
| ABHD2 | 0,296987002 |
| TLE3 | 0,297001046 |
| FLNA | 0,29721847 |
| PDIA6 | 0,297528055 |

| | |
|---|---|
| SEPT9 | 0,297629111 |
| MDC1 | 0,297817082 |
| CXorf21 | 0,29794868 |
| ENDOD1 | 0,298071994 |
| WDR1 | 0,298121563 |
| C3orf58 | 0,298314461 |
| AGA | 0,298316408 |
| IPCEF1 | 0,298901542 |
| KLF12 | 0,299500456 |
| FUT8 | 0,300312569 |
| SMARCA4 | 0,300594415 |
| MDN1 | 0,300768941 |
| TFRC | 0,301206393 |
| RNF144A | 0,301230922 |
| USP34 | 0,301693763 |
| AKAP11 | 0,30172819 |
| SEC24A | 0,301886123 |
| ZZEF1 | 0,303646968 |
| RACGAP1 | 0,303733568 |
| KIF14 | 0,304135027 |
| CEP128 | 0,304305443 |
| CCNA2 | 0,304580806 |
| RNASEH2A | 0,304702023 |
| SPRY2 | 0,305502779 |
| RNF26 | 0,305870155 |
| FLI1 | 0,305927722 |
| HNRNPUL2 | 0,306399254 |
| NUP214 | 0,306529074 |
| PIK3CG | 0,307054164 |
| KCNJ3 | 0,307363592 |
| CDH2 | 0,307378911 |
| CTNNAL1 | 0,307508345 |
| FNIP2 | 0,307676547 |
| KAT6A | 0,308228812 |
| DDI2 | 0,308492399 |
| TNFRSF11A | 0,308619365 |
| KIAA0513 | 0,308897161 |
| KIAA2018 | 0,309034867 |
| MGAT3 | 0,309125239 |
| HDHD1 | 0,309289662 |
| EP400 | 0,310751544 |
| UGGT1 | 0,311527213 |
| PAFAH2 | 0,313376428 |
| DACT1 | 0,313422091 |
| GIMAP6 | 0,313596225 |
| CEP350 | 0,315760644 |

| | |
|---|---|
| ANXA11 | 0,315887858 |
| SYNJ2 | 0,316084807 |
| GPAT3 | 0,316575295 |
| FBXW7 | 0,317482159 |
| ADPRH | 0,317869494 |
| RSC1A1 | 0,319881595 |
| INCENP | 0,32000712 |
| SYNE3 | 0,321584708 |
| CTCF | 0,321836122 |
| C1R | 0,322126659 |
| HS3ST1 | 0,322934086 |
| CENPL | 0,325227504 |
| MCM2 | 0,326387344 |
| ERMP1 | 0,327569613 |
| CEND1 | 0,327657536 |
| MCC | 0,328563432 |
| LUZP1 | 0,330741381 |
| PAG1 | 0,330957467 |
| NCOA3 | 0,331196671 |
| LAPTM5 | 0,331357786 |
| CDC25B | 0,331754531 |
| HCFC1 | 0,331785254 |
| FTL | 0,331803066 |
| SORL1 | 0,331847208 |
| ZNF880 | 0,333405793 |
| DCP2 | 0,333685963 |
| TMTC1 | 0,334125571 |
| NSD1 | 0,336245448 |
| POU2F1 | 0,337377561 |
| C10orf12 | 0,338101408 |
| ORC1 | 0,338438994 |
| DYNC1H1 | 0,3388466 |
| DST | 0,339365735 |
| SIPA1L3 | 0,33997626 |
| REV3L | 0,341919297 |
| HTT | 0,343485261 |
| TULP4 | 0,343825345 |
| ANK1 | 0,344037934 |
| HDAC9 | 0,344299909 |
| GIMAP8 | 0,34507651 |
| ZNF385A | 0,345312376 |
| TUBA1B | 0,345548331 |
| DTL | 0,345878872 |
| MMS22L | 0,347858156 |
| KLK1 | 0,349053625 |
| ABL2 | 0,349274579 |

| | |
|---|---|
| CNOT6L | 0,349559348 |
| TPX2 | 0,349739072 |
| ZNF106 | 0,34985516 |
| RBFOX2 | 0,350638496 |
| PPP3CA | 0,351176296 |
| ASCC3 | 0,351478825 |
| DHFR | 0,351593599 |
| SLF1 | 0,351981355 |
| ALG10 | 0,352427248 |
| MYBPC2 | 0,352832132 |
| BIRC6 | 0,352873212 |
| DSCC1 | 0,353375353 |
| HERC2 | 0,35394011 |
| ETS1 | 0,355554822 |
| HERC1 | 0,356024023 |
| NUDT15 | 0,356028894 |
| SPTY2D1 | 0,357644829 |
| MFHAS1 | 0,359041541 |
| TOP2A | 0,359736885 |
| SKA3 | 0,361545549 |
| ESCO2 | 0,362419163 |
| WHSC1 | 0,363658987 |
| OTUD1 | 0,363670018 |
| SHISA5 | 0,364239869 |
| FOXRED2 | 0,36429712 |
| GALNT7 | 0,365061832 |
| ALMS1 | 0,365204736 |
| AKAP5 | 0,365701446 |
| ZFHX3 | 0,36601546 |
| NNT | 0,36672497 |
| PRR11 | 0,366778422 |
| SPTBN1 | 0,367814982 |
| PINK1-AS | 0,368783768 |
| CKAP2L | 0,369458163 |
| CDKN3 | 0,370264996 |
| RN7SL2 | 0,370535093 |
| PRKDC | 0,372230122 |
| PRKACB | 0,374406363 |
| SMC1A | 0,376526408 |
| KCTD12 | 0,376975436 |
| TBC1D9 | 0,377779899 |
| KIF4A | 0,379077133 |
| SHCBP1 | 0,379616476 |
| IKZF1 | 0,380661002 |
| CD84 | 0,382227634 |
| LRP6 | 0,382360146 |

| | |
|---|---|
| UTRN | 0,384102533 |
| RAB30 | 0,385942865 |
| ZNF829 | 0,386218034 |
| FLJ42627 | 0,386289337 |
| HIRIP3 | 0,387262127 |
| FRYL | 0,387989092 |
| CDCA2 | 0,390569267 |
| APOBEC3C | 0,39197726 |
| SASH1 | 0,392159973 |
| PRDM1 | 0,392379809 |
| MYH10 | 0,393204324 |
| RBL1 | 0,393501352 |
| CCDC15 | 0,394962694 |
| FOXN3 | 0,395018362 |
| KMT2A | 0,397752086 |
| GGH | 0,397836447 |
| MBNL3 | 0,398507202 |
| VANGL1 | 0,401410551 |
| SLX4IP | 0,404557381 |
| LRRC16A | 0,404948112 |
| SMC2 | 0,405605844 |
| RRM1 | 0,40610047 |
| CORO2B | 0,407315862 |
| FAM111B | 0,408730119 |
| KMT2D | 0,408769899 |
| VPS13D | 0,410055132 |
| ITGB3 | 0,410462241 |
| TLR9 | 0,411887975 |
| ANK3 | 0,412044443 |
| PCDHGC3 | 0,412600388 |
| RAD51 | 0,418275309 |
| STARD13 | 0,418483526 |
| PTPRK | 0,421787451 |
| ROBO1 | 0,421997618 |
| CRIM1 | 0,423022946 |
| SEMA4A | 0,424387341 |
| CCDC141 | 0,424499901 |
| TRRAP | 0,425104015 |
| CPM | 0,430331532 |
| LOC103611081 | 0,433018302 |
| ARHGAP11A | 0,434141688 |
| ITPR2 | 0,436287834 |
| AHNAK | 0,43731253 |
| FRMPD3 | 0,438450574 |
| TREML2 | 0,438972337 |
| LIPH | 0,443949475 |

| | |
|---|---|
| CLSPN | 0,444403126 |
| ZNF462 | 0,444515162 |
| ZFP36L2 | 0,446363541 |
| CENPF | 0,446462019 |
| GLIPR1 | 0,448618574 |
| RIMS3 | 0,449432917 |
| FRAS1 | 0,450209156 |
| ILDR2 | 0,452412151 |
| MYBL2 | 0,455757366 |
| SCARB2 | 0,458066948 |
| KMT2C | 0,459995733 |
| LOC101927550 | 0,461196391 |
| CD109 | 0,461661733 |
| KCNV1 | 0,462853238 |
| ZBTB37 | 0,466374808 |
| TLN2 | 0,467561961 |
| CDC42BPA | 0,469387929 |
| KCNN3 | 0,469951894 |
| NEO1 | 0,480723242 |
| PKI55 | 0,485921833 |
| FBN1 | 0,485944931 |
| IER5L | 0,486528163 |
| SPSB1 | 0,486948805 |
| PLXDC2 | 0,487807466 |
| CABLES1 | 0,492040072 |
| WFS1 | 0,492815354 |
| KANK2 | 0,493540028 |
| PRTFDC1 | 0,499260637 |
| SOGA1 | 0,500762333 |
| PALLD | 0,502664486 |
| ARHGAP5 | 0,503110886 |
| TUBA4A | 0,508258218 |
| RGL1 | 0,514234055 |
| AMICA1 | 0,518699208 |
| NOTCH2 | 0,519277759 |
| FOXO3 | 0,519303691 |
| SMIM3 | 0,523934056 |
| AAK1 | 0,52681521 |
| WDFY3 | 0,527862041 |
| SEMA3D | 0,529337884 |
| TPRG1 | 0,529461791 |
| CREB3L1 | 0,53188822 |
| FOXO1 | 0,532219291 |
| CA5B | 0,533761651 |
| FILIP1 | 0,534477489 |
| TTN | 0,536944782 |

| | |
|---|---|
| GDF15 | 0,539752866 |
| ACER2 | 0,542979232 |
| ZNF784 | 0,548077011 |
| MICAL2 | 0,549224617 |
| KLLN | 0,551161768 |
| CBX5 | 0,551501338 |
| RNF213 | 0,553529481 |
| TLR3 | 0,555484049 |
| CPT1A | 0,556717085 |
| GPC4 | 0,556980981 |
| AMOTL1 | 0,557203682 |
| KLF5 | 0,559207191 |
| SMPD3 | 0,570731609 |
| CDCP1 | 0,571704308 |
| TMEM2 | 0,571739233 |
| NCKAP5 | 0,58064654 |
| PEG10 | 0,582335503 |
| CTTNBP2 | 0,596873461 |
| CCNE2 | 0,597078031 |
| MTUS1 | 0,598557557 |
| FZD7 | 0,602660361 |
| MITF | 0,612372162 |
| BMP2 | 0,620878928 |
| LINC01358 | 0,633244719 |
| FAT1 | 0,637849668 |
| FN1 | 0,638124098 |
| NKAIN2 | 0,64592732 |
| CADPS2 | 0,649986322 |
| MYRIP | 0,659399274 |
| KIAA2022 | 0,662856631 |
| TCAF2 | 0,664151973 |
| ARHGAP23 | 0,66510804 |
| AMZ1 | 0,670797804 |
| TENM2 | 0,681531833 |
| LOC102723373 | 0,682695202 |
| UBASH3B | 0,684929775 |
| ANXA1 | 0,693468905 |
| PLXNA2 | 0,713807996 |
| F2RL3 | 0,722596431 |
| SIGLEC15 | 0,736953118 |
| ZNF703 | 0,741713643 |
| CCNJL | 0,759155402 |
| PCDH7 | 0,767222781 |
| MFAP3L | 0,783636654 |
| LRCH2 | 0,786829051 |
| GNAQ | 0,788342851 |

| | |
|---|---|
| MAP1A | 0,791375931 |
| ADCY9 | 0,804152332 |
| PTPN14 | 0,817743693 |
| LIMD1-AS1 | 0,822805495 |
| FGL2 | 0,837940237 |
| RASA4B | 0,843530833 |
| LRP1 | 0,865149758 |
| ARHGAP31-AS1 | 0,869710647 |
| IL1B | 0,872835596 |
| DGCR11 | 0,875390708 |
| PAPSS2 | 0,878785995 |
| IGFBP3 | 0,893119388 |
| SLC24A3 | 0,895408435 |
| MIR5195 | 0,900245601 |
| SNTA1 | 0,937785681 |
| AGAP2-AS1 | 0,941692896 |
| MAGI2 | 0,966153315 |
| PTRF | 0,972831557 |
| CELSR1 | 0,982844974 |
| FOXP3 | 0,986968455 |
| ADAMTS8 | 3,07345E+12 |
| ZBED2 | 1,07315E+13 |
| RNA28S5 | 1,14566E+13 |
| NEBL | 1,1462E+13 |
| SLC26A7 | 1,36196E+13 |
| TGFBI | 1,41458E+13 |
| BMPR1B | 1,52477E+13 |
| UNC13C | 1,69251E+13 |
| CNTN6 | 1,88843E+13 |
| ROS1 | 2,03975E+13 |
| TRHDE-AS1 | 2,15835E+13 |
| LINC00508 | 2,35767E+13 |
| FST | 2,52123E+13 |
| ZNF99 | 2,5691E+13 |
| PCDHB11 | 2,60248E+13 |
| KRT9 | 2,69092E+13 |
| LOC101927769 | 2,71113E+13 |
| LOC100996291 | 2,75932E+13 |
| TMEM92 | 3,19726E+13 |
| KIAA1210 | 3,4517E+13 |
| C1orf137 | 4,0428E+13 |
| PRKG1-AS1 | 4,0428E+13 |
| PWRN1 | 4,0428E+13 |
| LILRA6 | 4,0428E+13 |
| SULT1C4 | 4,0428E+13 |

| | |
|---|---|
| PCK1 | 4,0428E+13 |
| LOC101928269 | 4,0428E+13 |
| SNTN | 4,0428E+13 |
| LINC01054 | 4,84847E+13 |
| LINC00488 | 1,00626E+14 |
| CYP1A1 | 1,02529E+14 |
| RNA45S5 | 1,03291E+14 |
| ADGRV1 | 1,04146E+14 |
| MYBPH | 1,04784E+14 |
| COL4A5 | 1,05106E+14 |
| SV2B | 1,0774E+14 |
| C14orf132 | 1,10162E+14 |
| GRIA3 | 1,11999E+14 |
| EHD3 | 1,12364E+14 |
| NR5A2 | 1,12436E+14 |
| F5 | 1,12667E+14 |
| HIST1H2BL | 1,12692E+14 |
| MMRN1 | 1,12791E+14 |
| ARMC2 | 1,13978E+14 |
| NWD1 | 1,1442E+14 |
| C1orf226 | 1,1567E+14 |
| ENPEP | 1,1603E+14 |
| SLC8A1 | 1,18006E+14 |
| OSBPL1A | 1,19598E+14 |
| VIL1 | 1,21682E+14 |
| EFCAB5 | 1,25611E+14 |
| THEMIS | 1,28939E+14 |
| BMP3 | 1,29414E+14 |
| AKR1C3 | 1,31754E+14 |
| ARHGEF10 | 1,32413E+14 |
| SCARNA7 | 1,34101E+14 |
| ST14 | 1,36711E+14 |
| GAS1 | 1,37114E+14 |
| MYOM3 | 1,38417E+14 |
| KIRREL3 | 1,39436E+14 |
| GNAT2 | 1,45562E+14 |
| HMCN2 | 1,46675E+14 |
| CRTC3-AS1 | 1,48841E+14 |
| FREM1 | 1,54575E+14 |
| CDK5R2 | 1,54782E+14 |
| EREG | 1,56322E+14 |
| SLC4A10 | 1,56419E+14 |
| CCBE1 | 1,59293E+14 |
| PLXDC1 | 1,59697E+14 |
| SCUBE3 | 1,63158E+14 |
| FLT4 | 1,63441E+14 |

| | |
|---|---|
| MET | 1,67698E+14 |
| LINC01234 | 1,70268E+14 |
| VSTM4 | 1,71646E+14 |
| SLC34A2 | 1,75359E+14 |
| DRAXIN | 1,75739E+14 |
| PAPPA | 1,7826E+14 |
| PLEKHD1 | 1,79384E+14 |
| DEAR | 1,84483E+14 |
| DLGAP2 | 1,87596E+14 |
| OR2T2 | 1,91259E+14 |
| THSD4 | 1,91631E+14 |
| GLI2 | 1,94076E+14 |
| LPPR5 | 1,94518E+14 |
| IL1A | 1,95692E+14 |
| ERICH6 | 1,96152E+14 |
| C1QL1 | 1,99842E+14 |
| MTMR7 | 2,00677E+14 |
| NRG1 | 2,04113E+14 |
| SPARCL1 | 2,04564E+14 |
| CUX2 | 2,05922E+14 |
| STEAP4 | 2,05968E+14 |
| ZNF704 | 2,06277E+14 |
| LOC414300 | 2,07163E+14 |
| MEP1B | 2,10392E+14 |
| ANKRD35 | 2,10466E+14 |
| PCDHGC4 | 2,11199E+14 |
| CCDC129 | 2,14811E+14 |
| HSD3BP4 | 2,15472E+14 |
| PDE10A | 2,16771E+14 |
| BIN3-IT1 | 2,17067E+14 |
| ANKK1 | 2,17901E+14 |
| PIWIL1 | 2,18959E+14 |
| ALX4 | 2,19432E+14 |
| ALDH1A2 | 2,19432E+14 |
| C1orf94 | 2,25643E+14 |
| PLSCR2 | 2,25943E+14 |
| ASPG | 2,26415E+14 |
| PEAR1 | 2,27049E+14 |
| LOC100129697 | 2,28466E+14 |
| FLG2 | 2,32867E+14 |
| KCNH7 | 2,33404E+14 |
| GALNT15 | 2,35776E+14 |
| IQCA1L | 2,41613E+14 |
| LIN28B | 2,41979E+14 |
| LINC00521 | 2,42689E+14 |
| RADIL | 2,42689E+14 |

| | |
|---|---|
| ADGRF1 | 2,43408E+14 |
| CDH18 | 2,44297E+14 |
| CDHR3 | 2,47275E+14 |
| GNA14 | 2,50783E+14 |
| SIGLEC8 | 2,5122E+14 |
| PLD5 | 2,5176E+14 |
| ADGRB3 | 2,52869E+14 |
| TECTB | 2,55361E+14 |
| JPH3 | 2,55885E+14 |
| LPCAT2 | 2,57457E+14 |
| KCNB1 | 2,59937E+14 |
| CPN2 | 2,60186E+14 |
| PDZD3 | 2,62604E+14 |
| CYP4F22 | 2,62809E+14 |
| LOC101926962 | 2,63406E+14 |
| COLEC11 | 2,63826E+14 |
| CLCA4 | 2,64302E+14 |
| KRT80 | 2,65543E+14 |
| BEST3 | 2,67166E+14 |
| C11orf96 | 2,68177E+14 |
| GOLGA6L17P | 2,69475E+14 |
| TPH2 | 2,69585E+14 |
| CSMD2-AS1 | 2,70215E+14 |
| ZNF806 | 2,721E+14 |
| KLHL31 | 2,74614E+14 |
| CA12 | 2,76267E+14 |
| S100B | 2,77366E+14 |
| SNORD36B | 2,77366E+14 |
| AGBL1 | 2,77798E+14 |
| LINC01182 | 2,77798E+14 |
| LOC401242 | 2,77798E+14 |
| CXorf36 | 2,81207E+14 |
| SPINK5 | 2,83041E+14 |
| DNAJC22 | 2,84193E+14 |
| LOC442132 | 2,86738E+14 |
| LOC101928519 | 2,87715E+14 |
| TDRD6 | 2,87715E+14 |
| LOC100133077 | 2,87715E+14 |
| PAX7 | 2,87769E+14 |
| SLC5A8 | 2,89819E+14 |
| VAX1 | 2,94274E+14 |
| CLCA3P | 2,97267E+14 |
| ZDHHC15 | 3,00262E+14 |
| DCX | 3,00363E+14 |
| KCNJ5 | 3,00557E+14 |
| LOC100128531 | 3,00557E+14 |

| | |
|---|---|
| CYR61 | 3,03592E+14 |
| OTOP3 | 3,03592E+14 |
| ST7-OT3 | 3,03663E+14 |
| TMC4 | 3,06887E+14 |
| KCNK2 | 3,09578E+14 |
| SERPINA4 | 3,12848E+14 |
| CLRN1 | 3,12848E+14 |
| PNLIPRP1 | 3,1475E+14 |
| GNAT1 | 3,1475E+14 |
| ZNF804B | 3,15388E+14 |
| TMEM47 | 3,18172E+14 |
| CFAP221 | 3,22969E+14 |
| CADM3-AS1 | 3,24434E+14 |
| LINC01180 | 3,25655E+14 |
| RBM46 | 3,2688E+14 |
| SPOCK3 | 3,2688E+14 |
| TRIM43 | 3,30067E+14 |
| LOC643542 | 3,34029E+14 |
| CD1E | 3,3517E+14 |
| RNU6ATAC | 3,4014E+14 |
| ADH7 | 3,45286E+14 |
| MMP10 | 3,47609E+14 |
| SMOC2 | 3,54992E+14 |
| CDC14C | 3,58002E+14 |
| LINC01222 | 3,60231E+14 |
| LOC283299 | 3,60231E+14 |
| SACS-AS1 | 3,60231E+14 |
| REREP3 | 3,60231E+14 |
| RNU6-10P | 3,60231E+14 |
| PRDM14 | 3,64614E+14 |
| LOC284950 | 3,65955E+14 |
| NPTX2 | 3,66065E+14 |
| LINC00964 | 3,70812E+14 |
| ADRB3 | 3,72559E+14 |
| LINC01492 | 3,72559E+14 |
| LEFTY2 | 3,78001E+14 |
| AKR1B10 | 3,82179E+14 |
| EPCAM | 3,89829E+14 |
| WNT8A | 3,89829E+14 |
| LINC01375 | 3,90265E+14 |
| TRIM49C | 3,90627E+14 |
| SIGLECL1 | 3,96655E+14 |
| GPR37 | 3,96655E+14 |
| DLX3 | 4,09747E+14 |
| LOC100506085 | 4,09747E+14 |
| AFAP1-AS1 | 4,16788E+14 |

| | |
|---|---|
| OR10A3 | 4,20275E+14 |
| LOC101928778 | 4,22253E+14 |
| CD3G | 4,22253E+14 |
| PSG5 | 4,22409E+14 |
| PRSS48 | 4,22409E+14 |
| PLCZ1 | 4,38597E+14 |
| SULT1B1 | 4,38597E+14 |
| WNT16 | 4,38597E+14 |
| CLEC12B | 4,39108E+14 |
| LOC101927653 | 4,39108E+14 |
| GYPE | 4,53214E+14 |
| ARSE | 4,53214E+14 |
| LINC00554 | 4,54186E+14 |
| MGC27382 | 4,66533E+14 |
| KLHL40 | 4,66533E+14 |
| SERP2 | 4,67827E+14 |
| TCP10L2 | 4,67827E+14 |
| CD2 | 4,78764E+14 |
| OMP | 4,80281E+14 |
| MIR30E | 4,89315E+14 |
| PDZK1IP1 | 4,89315E+14 |
| LOC100996263 | 4,89315E+14 |
| OR51A7 | 4,89315E+14 |
| GPHA2 | 4,89315E+14 |
| FAM181A | 4,89315E+14 |
| LINC00483 | 4,89315E+14 |
| MIR216B | 4,89315E+14 |
| LINC00489 | 4,89315E+14 |
| SPINT3 | 4,89315E+14 |
| LOC101929412 | 4,89315E+14 |
| NUPR1L | 4,89315E+14 |
| TAS2R38 | 4,89315E+14 |
| DEFA6 | 4,89315E+14 |
| LINC00208 | 4,89315E+14 |
| LINC00548 | 4,98479E+14 |
| KRT4 | 5,08601E+14 |
| LINC01143 | 5,08601E+14 |
| KRTAP13-1 | 5,08601E+14 |
| ARHGEF3-AS1 | 5,08601E+14 |
| OTOL1 | 5,08601E+14 |
| TMED11P | 5,08601E+14 |
| LOC102477328 | 5,08601E+14 |
| LOC100128993 | 5,08601E+14 |
| KLRF2 | 5,21472E+14 |
| HIST2H2BA | 5,2561E+14 |
| FAM163A | 5,2561E+14 |

| | |
|---|---|
| LOC101927787 | 5,2561E+14 |
| THPO | 5,2561E+14 |
| MEP1A | 5,2823E+14 |
| IFNL4 | 5,40823E+14 |
| SSX5 | 5,54585E+14 |
| OLAH | 5,67147E+14 |

Supplementary Table 10. Dysregulated genes (both downregulated and upregulated) identified upon comparison of ataluren EP2-treated cells versus untreated controls.

Down-regulated

| Gene | log2FoldChange |
|---|---|
| NELL2 | -6,93587E+14 |
| CRX | -6,91785E+14 |
| GPA33 | -6,84302E+14 |
| ADGRF5 | -6,84302E+14 |
| HYDIN2 | -6,79683E+14 |
| SLC9C1 | -6,73048E+14 |
| ADAM33 | -6,72833E+14 |
| PPP1R9A | -6,72833E+14 |
| SLC14A2-AS1 | -6,71866E+14 |
| AMBN | -6,71866E+14 |
| LOC101927666 | -6,68217E+14 |
| LINC01492 | -6,66947E+14 |
| ZNF366 | -6,64592E+14 |
| HEPHL1 | -6,6428E+14 |
| CDH3 | -6,6217E+14 |
| SLC26A5 | -6,62063E+14 |
| ATP1A2 | -6,5955E+14 |
| MOG | -6,58244E+14 |
| GLRA2 | -6,55143E+14 |
| TSPYL6 | -6,52904E+14 |
| LOC442028 | -6,52904E+14 |
| NDST3 | -6,52904E+14 |
| ENPP3 | -6,52904E+14 |
| BPIFB1 | -6,51506E+14 |
| KNG1 | -6,51506E+14 |
| LOC101927766 | -6,50233E+14 |
| WFDC1 | -6,50068E+14 |
| XIST | -6,50068E+14 |
| ALS2CR11 | -6,46028E+14 |
| SLC47A1 | -6,44587E+14 |
| B3GNT3 | -6,44587E+14 |
| SYT3 | -6,44587E+14 |
| LINC00547 | -6,41599E+14 |
| GCSAML | -6,40274E+14 |
| RFX4 | -6,38787E+14 |
| MIR124-2HG | -6,35941E+14 |
| PIANP | -6,3576E+14 |
| CCDC170 | -6,3576E+14 |
| PCDHA1 | -6,34339E+14 |
| LOC101928150 | -6,34201E+14 |

| | |
|---|---|
| TRPM5 | -6,3275E+14 |
| ST8SIA3 | -6,31166E+14 |
| LOC284865 | -6,31166E+14 |
| GRIA2 | -6,31166E+14 |
| IL21-AS1 | -6,27978E+14 |
| C5orf47 | -6,27978E+14 |
| TMEM255B | -6,26615E+14 |
| CYP4A22 | -6,26354E+14 |
| GABRA1 | -6,26354E+14 |
| TERT | -6,24872E+14 |
| PKD1L3 | -6,24697E+14 |
| KLHL1 | -6,23155E+14 |
| MIR8078 | -6,23155E+14 |
| GIPC2 | -6,22995E+14 |
| ADAMTS14 | -6,22995E+14 |
| LGI2 | -6,21449E+14 |
| TSG1 | -6,21449E+14 |
| FGF1 | -6,21231E+14 |
| DKK3 | -6,19743E+14 |
| DKFZP434A062 | -6,19743E+14 |
| MYOM3 | -6,18027E+14 |
| LOC440390 | -6,18027E+14 |
| LAMB4 | -6,18027E+14 |
| LACTBL1 | -6,1629E+14 |
| BMP4 | -6,1629E+14 |
| TMC5 | -6,1629E+14 |
| HTR2B | -6,1629E+14 |
| CDH18 | -6,1629E+14 |
| GRM6 | -6,1629E+14 |
| TRIML2 | -6,14764E+14 |
| LOC101929488 | -6,14764E+14 |
| BDKRB2 | -6,1452E+14 |
| ECEL1 | -6,1452E+14 |
| CNTN6 | -6,1452E+14 |
| ZIC1 | -6,1452E+14 |
| EBF2 | -6,1452E+14 |
| LOC101927623 | -6,1452E+14 |
| ADGRG4 | -6,1452E+14 |
| ARMC4 | -6,12703E+14 |
| LOC646903 | -6,12703E+14 |
| CD163L1 | -6,11037E+14 |
| TDRD1 | -6,09191E+14 |
| APOA4 | -6,09191E+14 |
| LOC283177 | -6,09191E+14 |
| FAM181A-AS1 | -6,09191E+14 |
| GSG1L | -6,09191E+14 |

| | |
|---|---|
| FAR2P1 | -6,09191E+14 |
| FOXF2 | -6,09191E+14 |
| H2BFM | -6,09191E+14 |
| HTR2A | -6,08841E+14 |
| RGSL1 | -6,0734E+14 |
| PROX1-AS1 | -6,0734E+14 |
| LINC01561 | -6,0734E+14 |
| KRT74 | -6,0734E+14 |
| LOC100505918 | -6,06613E+14 |
| LINC00482 | -6,05469E+14 |
| BPIFB3 | -6,05469E+14 |
| SLC36A2 | -6,05469E+14 |
| LINC01500 | -6,03566E+14 |
| RNF112 | -6,03566E+14 |
| ANTXR1 | -6,03566E+14 |
| FRMD1 | -6,03566E+14 |
| LINC00597 | -6,01858E+14 |
| LOC284344 | -6,01858E+14 |
| ARID3C | -6,01858E+14 |
| MMP12 | -6,01616E+14 |
| SIAH3 | -6,01616E+14 |
| ZP4 | -5,99826E+14 |
| NEUROD4 | -5,99826E+14 |
| GABRD | -5,99598E+14 |
| C16orf89 | -5,99598E+14 |
| PCSK2 | -5,99598E+14 |
| VSX1 | -5,99598E+14 |
| ZIC4 | -5,99598E+14 |
| MEPE | -5,99598E+14 |
| GABRR1 | -5,99598E+14 |
| MUSK | -5,99598E+14 |
| CSF2RA | -5,99598E+14 |
| MGAT4C | -5,97475E+14 |
| SLC1A6 | -5,97475E+14 |
| PSG4 | -5,97475E+14 |
| LOC728084 | -5,95798E+14 |
| GRB14 | -5,95798E+14 |
| GPIHBP1 | -5,95798E+14 |
| NR0B1 | -5,95798E+14 |
| LINC00839 | -5,95087E+14 |
| MSI1 | -5,95087E+14 |
| MYL1 | -5,95087E+14 |
| SPO11 | -5,95087E+14 |
| FZD10 | -5,93769E+14 |
| SNHG24 | -5,93769E+14 |
| LILRA1 | -5,93769E+14 |

| | |
|---|---|
| CYP27C1 | -5,93769E+14 |
| MLPH | -5,93769E+14 |
| EYA2 | -5,93769E+14 |
| FOXP1-AS1 | -5,93769E+14 |
| F11 | -5,93769E+14 |
| CYP4X1 | -5,91709E+14 |
| LOC283214 | -5,91709E+14 |
| TMEM132D | -5,91709E+14 |
| BNC1 | -5,91709E+14 |
| CCDC144NL-AS1 | -5,91709E+14 |
| DUXA | -5,91709E+14 |
| OLIG2 | -5,91709E+14 |
| CXCL1 | -5,91709E+14 |
| RBM46 | -5,91709E+14 |
| FAM87A | -5,91709E+14 |
| LOC392232 | -5,91709E+14 |
| LINC01606 | -5,89964E+14 |
| AQP4-AS1 | -5,89601E+14 |
| OR7A5 | -5,89601E+14 |
| KCNJ16 | -5,87691E+14 |
| LYPD4 | -5,87691E+14 |
| TNFRSF6B | -5,87691E+14 |
| SPATA12 | -5,87691E+14 |
| LOC101927374 | -5,87691E+14 |
| CLEC5A | -5,87691E+14 |
| LINC01296 | -5,87422E+14 |
| TMEM56 | -5,85467E+14 |
| LRP3 | -5,85133E+14 |
| ZMAT4 | -5,85133E+14 |
| HNRNPCL1 | -5,83256E+14 |
| CLCNKB | -5,83256E+14 |
| LRRC7 | -5,83256E+14 |
| DKFZP434L187 | -5,83256E+14 |
| ANKRD30B | -5,83256E+14 |
| DMRTC2 | -5,83256E+14 |
| TRPM4 | -5,83256E+14 |
| RHAG | -5,83256E+14 |
| LOC101926964 | -5,81036E+14 |
| LOC101928436 | -5,81036E+14 |
| EPS8L3 | -5,81036E+14 |
| GLYATL1 | -5,81036E+14 |
| KRT25 | -5,81036E+14 |
| OR7E91P | -5,81036E+14 |
| LINC01310 | -5,81036E+14 |
| LRRN1 | -5,81036E+14 |

| | |
|---|---|
| PCDHA10 | -5,81036E+14 |
| GREM2 | -5,7649E+14 |
| FENDRR | -5,7649E+14 |
| DAW1 | -5,7649E+14 |
| LINC01524 | -5,7649E+14 |
| VGLL3 | -5,7649E+14 |
| CCDC37 | -5,7649E+14 |
| SLC51A | -5,7649E+14 |
| GC | -5,7649E+14 |
| KLHL38 | -5,7649E+14 |
| OR7E156P | -5,71629E+14 |
| PDLIM3 | -5,71629E+14 |
| TRPV6 | -5,71629E+14 |
| RNU4-1 | -4,29481E+14 |
| RNA28S5 | -4,01737E+14 |
| KNDC1 | -4,01229E+14 |
| TMEFF2 | -3,96834E+14 |
| DTNA | -3,8936E+14 |
| FSIP2 | -3,8628E+14 |
| RNU4-2 | -3,81867E+14 |
| SVEP1 | -3,81475E+14 |
| SLC24A2 | -3,78104E+14 |
| SLC4A3 | -3,7474E+14 |
| PPFIA2 | -3,73902E+14 |
| LONRF2 | -3,73902E+14 |
| PTGER3 | -3,73016E+14 |
| SLC12A1 | -3,71353E+14 |
| DSCAM | -3,67776E+14 |
| LOC100131257 | -3,63145E+14 |
| MUC5B | -3,58838E+14 |
| RIMS1 | -3,5858E+14 |
| RUNX1T1 | -3,57529E+14 |
| IL22RA2 | -3,55603E+14 |
| NOS1 | -3,54925E+14 |
| ASTN1 | -3,50647E+14 |
| RMRP | -3,50106E+14 |
| SLC9C2 | -3,47664E+14 |
| XDH | -3,45483E+14 |
| SLIT3 | -3,41342E+14 |
| GREB1L | -3,41302E+14 |
| BRSK2 | -3,41248E+14 |
| VEPH1 | -3,40283E+14 |
| TNC | -3,39117E+14 |
| RBFOX1 | -3,39062E+14 |
| STXBP5L | -3,39052E+14 |
| NCAM1 | -3,38401E+14 |

| | |
|---|---|
| VWA3B | -3,36901E+14 |
| MRO | -3,34727E+14 |
| CDHR3 | -3,34633E+14 |
| ZNF704 | -3,27664E+14 |
| MICU3 | -3,25304E+14 |
| FAM71F1 | -3,24099E+14 |
| BEAN1 | -3,22901E+14 |
| MROH2A | -3,22845E+14 |
| KIF26A | -3,21705E+14 |
| RIPPLY2 | -3,21705E+14 |
| ABCA8 | -3,20505E+14 |
| RN7SK | -3,19907E+14 |
| ENPP7 | -3,17222E+14 |
| C1orf21 | -3,14125E+14 |
| SCN10A | -3,13615E+14 |
| SGCD | -3,12852E+14 |
| ADAMTS2 | -3,11836E+14 |
| TPTE2P1 | -3,07505E+14 |
| CORIN | -3,05972E+14 |
| CRB1 | -3,05299E+14 |
| FAT3 | -3,01332E+14 |
| SLC4A10 | -3,01029E+14 |
| ABCB11 | -2,96025E+14 |
| APOB | -2,95442E+14 |
| SLC4A1 | -2,92431E+14 |
| SDK2 | -2,8953E+14 |
| CSMD1 | -2,89316E+14 |
| LRRC9 | -2,89157E+14 |
| ADAMTS18 | -2,89157E+14 |
| HYDIN | -2,8706E+14 |
| LOXHD1 | -2,85039E+14 |
| TLL1 | -2,83862E+14 |
| HIST1H1B | -2,79839E+14 |
| SULF1 | -2,77758E+14 |
| ARHGEF4 | -2,74793E+14 |
| DPYSL5 | -2,7385E+14 |
| TMEM59L | -2,70292E+14 |
| KIRREL | -2,66871E+14 |
| GRM5 | -2,66871E+14 |
| ARAP3 | -2,66871E+14 |
| CEP126 | -2,64876E+14 |
| ERBB4 | -2,62394E+14 |
| LOC101929541 | -2,53908E+14 |
| SH3TC2 | -2,49667E+14 |
| COL20A1 | -2,49358E+14 |
| ENAH | -2,47612E+14 |

| | |
|---|---|
| FLT4 | -2,4676E+14 |
| SGIP1 | -2,43357E+14 |
| MYT1L | -2,42457E+14 |
| PTPRQ | -2,41642E+14 |
| RYR2 | -2,38309E+14 |
| PKHD1 | -2,38084E+14 |
| TMEM54 | -2,36785E+14 |
| ESYT3 | -2,35965E+14 |
| WNK3 | -2,34648E+14 |
| CACNA1F | -2,25377E+14 |
| KSR2 | -2,2047E+14 |
| COBL | -2,14769E+14 |
| MYO15A | -2,06521E+14 |
| ARFGEF3 | -2,05839E+14 |
| MUC17 | -2,04702E+14 |
| BACE2 | -1,98845E+14 |
| SLC34A2 | -1,96168E+14 |
| ENAM | -1,92461E+14 |
| TCP10L | -1,90354E+14 |
| LAMB2 | -1,8299E+14 |
| DNAH3 | -1,80991E+14 |
| PLCD4 | -1,77921E+14 |
| HIST1H1E | -1,71275E+14 |
| TP53I11 | -1,69746E+14 |
| OTOF | -1,57796E+14 |
| LRP2 | -1,57359E+14 |
| EML6 | -1,54713E+14 |
| CACNA1C | -1,51994E+14 |
| SCARNA7 | -1,44407E+14 |
| CCDC30 | -1,34631E+14 |
| PTCHD2 | -1,33342E+14 |
| DNAH11 | -1,31616E+14 |
| RASAL2 | -1,25935E+14 |
| DNAH10 | -1,23643E+14 |
| SPRED3 | -1,1254E+14 |
| PTPRN2 | -1,05037E+14 |
| ABCC8 | -6,98757E+13 |
| NR2F1-AS1 | -6,92747E+13 |
| ACAN | -6,7879E+13 |
| NTRK3 | -6,68305E+13 |
| CRTAC1 | -6,29579E+13 |
| MGC39584 | -6,29579E+13 |
| CARD10 | -6,29579E+13 |
| FAM90A25P | -6,29579E+13 |
| GAS2L2 | -6,12889E+13 |
| LOC100505716 | -6,12889E+13 |

| | |
|---|---|
| PRICKLE2-AS1 | -6,06223E+13 |
| MGC27382 | -5,78787E+13 |
| KRT73-AS1 | -5,78787E+13 |
| SP7 | -5,78787E+13 |
| PWAR1 | -5,78787E+13 |
| PDZD9 | -5,78787E+13 |
| KCTD19 | -5,78787E+13 |
| RBFADN | -5,78787E+13 |
| LOC641367 | -5,78787E+13 |
| SFTPB | -5,78787E+13 |
| GPR149 | -5,78787E+13 |
| CNGA1 | -5,78787E+13 |
| ADH1B | -5,78787E+13 |
| GRIK2 | -5,78787E+13 |
| GHRHR | -5,78787E+13 |
| OPRK1 | -5,78787E+13 |
| LINC01289 | -5,78787E+13 |
| HAS2-AS1 | -5,78787E+13 |
| GYG2 | -5,78787E+13 |
| YWHAEP7 | -3,71133E+13 |
| MEG3 | -3,549E+13 |
| PRSS23 | -3,51736E+13 |
| PTPRT | -3,43422E+13 |
| PPP1R1B | -3,32345E+13 |
| COL4A1 | -3,08088E+13 |
| KCNC1 | -3,04776E+13 |
| PCDH10 | -2,4143E+13 |
| DNAH14 | -2,25982E+13 |
| ADGRV1 | -1,90537E+13 |
| OLFML2B | -5,97812E+12 |
| HOXD3 | -5,97812E+12 |
| CNTN3 | -5,97812E+12 |
| PCDHA9 | -5,97812E+12 |
| LMX1B | -5,97812E+12 |
| AIM1L | -5,74118E+12 |
| CD1D | -5,74118E+12 |
| KERA | -5,74118E+12 |
| EPX | -5,74118E+12 |
| LOC284395 | -5,74118E+12 |
| LOC645949 | -5,74118E+12 |
| OR5K1 | -5,74118E+12 |
| NPY6R | -5,74118E+12 |
| MYH16 | -5,74118E+12 |
| NUP210L | -3,36845E+12 |
| LIFR | -2,68541E+12 |
| GLYATL2 | -2,49295E+12 |

| | |
|---|---|
| ALOX12B | -0,901459356 |
| MYLK4 | -0,867525617 |
| SORBS1 | -0,76423671 |
| EGR3 | -0,724237678 |
| BEX5 | -0,709534938 |
| HOXB9 | -0,64757502 |
| EGR1 | -0,638083241 |
| SNORD104 | -0,619891233 |
| MIR17HG | -0,597979123 |
| ABCA3 | -0,594069272 |
| WBP1 | -0,559173783 |
| JAKMIP2 | -0,539021565 |
| SNPH | -0,52496445 |
| LOC728613 | -0,499114005 |
| ENOX1 | -0,497654786 |
| HIST1H2BC | -0,472010308 |
| RBMS2 | -0,454027326 |
| ANKRD42 | -0,452790959 |
| AK7 | -0,450894901 |
| FAHD2CP | -0,449002007 |
| SPATA7 | -0,448593743 |
| ZSCAN2 | -0,432506058 |
| C10orf10 | -0,419776719 |
| HOXC6 | -0,417523808 |
| IER3 | -0,41359194 |
| OTX1 | -0,392444107 |
| CDADC1 | -0,388619994 |
| CNPY4 | -0,364404228 |
| PYGL | -0,357157396 |
| BCL2A1 | -0,356292456 |
| NAT6 | -0,349738143 |
| ENTPD2 | -0,344247051 |
| BCL2L11 | -0,332552552 |
| PINK1 | -0,326803374 |
| DNAJB4 | -0,305920001 |
| LXN | -0,302254653 |
| DNAJC24 | -0,295039018 |
| TMEM175 | -0,293803029 |
| CBR1 | -0,291668318 |
| PPP1R16A | -0,287645249 |
| CYP4V2 | -0,282404761 |
| ZNF354A | -0,278720934 |
| ARHGEF11 | -0,275210001 |
| MCAM | -0,273296352 |
| TCEAL4 | -0,268683238 |
| MSX1 | -0,262694366 |

| | |
|---|---|
| RNF19B | -0,260257452 |
| TARSL2 | -0,259157186 |
| IQCE | -0,258535968 |
| LEF1 | -0,255185258 |
| KRCC1 | -0,254237926 |
| PPP1R13B | -0,248601161 |
| ABHD4 | -0,245725682 |
| CETN3 | -0,244973312 |
| ZCRB1 | -0,244173001 |
| ACKR3 | -0,241516989 |
| CBWD1 | -0,240256559 |
| KRT10 | -0,240157965 |
| CCR7 | -0,238201212 |
| DYNC2LI1 | -0,237946448 |
| NSRP1 | -0,237733178 |
| RNF216P1 | -0,236099318 |
| FAM172A | -0,235488959 |
| FAIM | -0,235290429 |
| KCTD13 | -0,234574121 |
| ARL2 | -0,2333097 |
| MOXD1 | -0,233063504 |
| SIAH2 | -0,232762059 |
| CYSTM1 | -0,232552751 |
| TRAF3IP1 | -0,228889871 |
| NKIRAS1 | -0,227448905 |
| ARL2BP | -0,225326181 |
| DCAKD | -0,22488584 |
| DLX2 | -0,222865686 |
| CHMP4A | -0,222478339 |
| CFDP1 | -0,222053738 |
| MYPOP | -0,221444445 |
| NPNT | -0,221366597 |
| CIR1 | -0,220869582 |
| CD86 | -0,220702959 |
| C4orf32 | -0,220650271 |
| ZNF213 | -0,218686 |
| TIRAP | -0,218506812 |
| TULP3 | -0,217661566 |
| AKT1S1 | -0,217092252 |
| WDR60 | -0,217061969 |
| UBLCP1 | -0,216451938 |
| CALCOCO1 | -0,214129764 |
| CEP290 | -0,213659023 |
| PIK3R2 | -0,212724673 |
| FAM50A | -0,21170732 |
| TMEM63B | -0,211660806 |

| | |
|---|---|
| LINC01215 | -0,211178603 |
| FOXP4 | -0,210335957 |
| CDC14A | -0,210312122 |
| IFT74 | -0,210184178 |
| ARID4A | -0,210056791 |
| RAB12 | -0,20867208 |
| STX4 | -0,208491587 |
| DDX10 | -0,208362704 |
| SP140 | -0,208355962 |
| RAB29 | -0,207985234 |
| GADD45GIP1 | -0,206649654 |
| VIM | -0,206648809 |
| R3HCC1 | -0,204856645 |
| TOM1L2 | -0,204849545 |
| PAPD5 | -0,204579686 |
| NABP2 | -0,20448612 |
| MIA3 | -0,203637704 |
| MSC-AS1 | -0,203372233 |
| NELFE | -0,203294588 |
| HGSNAT | -0,203229888 |
| RAB2B | -0,203088121 |
| C12orf65 | -0,2028805 |
| BRD7 | -0,202228514 |
| TMA7 | -0,202154275 |
| FRA10AC1 | -0,199509828 |
| LEPROTL1 | -0,198616678 |
| MARCH9 | -0,198474338 |
| TMEM50B | -0,198437956 |
| STRADA | -0,197773235 |
| BCL2L1 | -0,197153726 |
| SRI | -0,196730354 |
| MNAT1 | -0,195733278 |
| TMOD2 | -0,195383142 |
| FTH1 | -0,194925793 |
| CCDC6 | -0,194901425 |
| CBWD2 | -0,193773789 |
| HIVEP3 | -0,193008838 |
| FAM127A | -0,192087456 |
| WAC-AS1 | -0,191811509 |
| ZNF768 | -0,19126511 |
| EDF1 | -0,191172198 |
| CCDC124 | -0,189828993 |
| RAB1A | -0,189702971 |
| EIF3J | -0,189560813 |
| ITFG3 | -0,18871553 |
| NUFIP1 | -0,188349972 |

| | |
|---|---|
| CLIP2 | -0,188190632 |
| TCEB3 | -0,187918662 |
| ZFPL1 | -0,187599088 |
| INSIG1 | -0,186462613 |
| PGD | -0,185975763 |
| HKR1 | -0,185478745 |
| PURA | -0,185368412 |
| BSDC1 | -0,184723546 |
| JUNB | -0,184633273 |
| TDRD3 | -0,184520348 |
| UPF3B | -0,184254514 |
| TBCK | -0,183730388 |
| PHF14 | -0,182848025 |
| RWDD4 | -0,182611957 |
| TSPAN3 | -0,182364604 |
| UNC119 | -0,181858019 |
| SYF2 | -0,18183926 |
| DYNLRB1 | -0,181303514 |
| UBAP2L | -0,180912437 |
| IK | -0,180555297 |
| DAG1 | -0,179589777 |
| BCL3 | -0,179213856 |
| PARM1 | -0,178972803 |
| TOX2 | -0,178547485 |
| NFKBIE | -0,178206056 |
| ST6GALNAC6 | -0,178065544 |
| CYB5A | -0,177803627 |
| IFIT2 | -0,177543796 |
| YLPM1 | -0,177236811 |
| ARHGAP31 | -0,176753027 |
| OXTR | -0,176593805 |
| HMGN5 | -0,176517408 |
| TRAPPC1 | -0,17631988 |
| FAM65A | -0,176206355 |
| RAB11FIP3 | -0,176003576 |
| SLC4A1AP | -0,175806319 |
| CDC42EP3 | -0,175335288 |
| TUBA1A | -0,174885902 |
| PPID | -0,174682227 |
| PDCD5 | -0,174584014 |
| CCSER2 | -0,17456625 |
| GOPC | -0,174072425 |
| BTN2A2 | -0,172590948 |
| GSK3A | -0,171905913 |
| EBF1 | -0,171832661 |
| UBXN1 | -0,171085263 |

| | |
|---|---|
| BRPF3 | -0,17063162 |
| GATAD1 | -0,170467438 |
| C7orf73 | -0,169921438 |
| CPT1A | -0,169618646 |
| RBM38 | -0,169294518 |
| FLNB | -0,169017114 |
| TRIP10 | -0,168643786 |
| PRR14 | -0,16826743 |
| ZMIZ2 | -0,167923203 |
| PPIL4 | -0,167445125 |
| SOCS1 | -0,167392677 |
| ARL8A | -0,166895792 |
| MESDC2 | -0,16688 |
| NFKB2 | -0,166797592 |
| CBLL1 | -0,166364757 |
| CBX1 | -0,165504952 |
| CRIP1 | -0,165211319 |
| HMGN3 | -0,164771698 |
| LHX2 | -0,163941093 |
| TSPYL1 | -0,163789739 |
| BRD3 | -0,16372136 |
| TMCC3 | -0,163048084 |
| TRAPPC6B | -0,162455681 |
| ZNF664 | -0,161413215 |
| WBP2 | -0,160925977 |
| CIZ1 | -0,160837419 |
| ATN1 | -0,160747149 |
| BRD2 | -0,16031923 |
| PREPL | -0,160049308 |
| GDI1 | -0,158872139 |
| RPL23A | -0,158678807 |
| EGR2 | -0,158398746 |
| PPDPF | -0,158243263 |
| SHFM1 | -0,158227565 |
| ORAI1 | -0,158072368 |
| BLK | -0,158014314 |
| LMTK2 | -0,157877311 |
| RAB7A | -0,157485624 |
| TOB1 | -0,157174996 |
| RELA | -0,156906604 |
| CSTB | -0,156887191 |
| RBM25 | -0,156863674 |
| SNRPD1 | -0,156725162 |
| HTATSF1 | -0,156593693 |
| IER2 | -0,156297163 |
| C21orf59 | -0,156028774 |

| | |
|---|---|
| EVI2B | -0,155294064 |
| TMEM57 | -0,155115317 |
| SLAMF1 | -0,154503568 |
| SCLT1 | -0,154328442 |
| CD2BP2 | -0,154326284 |
| NFKBIB | -0,154034358 |
| NPRL3 | -0,153953962 |
| PRKACA | -0,153953752 |
| UPF3A | -0,153806535 |
| SUGP1 | -0,152924372 |
| NKIRAS2 | -0,152826227 |
| R3HDM1 | -0,1527246 |
| CD44 | -0,152084959 |
| CASC3 | -0,151884028 |
| SAMD4B | -0,151832945 |
| CRK | -0,151759803 |
| HNRNPH3 | -0,151395477 |
| SOX9 | -0,151273467 |
| REL | -0,151267574 |
| NOLC1 | -0,150874078 |
| EPM2AIP1 | -0,150370045 |
| PIP5K1C | -0,150009166 |
| ERICH1 | -0,149842764 |
| SNRNP27 | -0,148794695 |
| NCL | -0,148678572 |
| TAX1BP1 | -0,148481659 |
| MRPL10 | -0,148068697 |
| PRPF38B | -0,147812467 |
| SRRM1 | -0,147621709 |
| EIF3A | -0,146952382 |
| KDM2B | -0,146789236 |
| TAF6 | -0,146763333 |
| TSG101 | -0,146239037 |
| HNRNPR | -0,145999375 |
| MRFAP1L1 | -0,144630769 |
| SMARCE1 | -0,144256433 |
| EHD1 | -0,143667903 |
| PRCC | -0,143581203 |
| MAP3K4 | -0,142792843 |
| CD55 | -0,142791028 |
| AP3D1 | -0,14272508 |
| KLC2 | -0,142697062 |
| WIPF2 | -0,14239146 |
| CC2D1A | -0,141826062 |
| NEMF | -0,141451248 |
| PITPNM1 | -0,141203229 |

| | |
|---|---|
| DPF2 | -0,140820887 |
| SSB | -0,140190418 |
| DHX38 | -0,139869181 |
| ABCF1 | -0,139467779 |
| PPM1K | -0,138951126 |
| BCL7B | -0,138497484 |
| AGPAT1 | -0,138240701 |
| ATF7 | -0,137329654 |
| TERF2IP | -0,136976395 |
| C9orf78 | -0,136489717 |
| CNOT3 | -0,136207237 |
| ZC3H18 | -0,135390017 |
| EIF5B | -0,135303211 |
| KIF1C | -0,135165232 |
| PSMF1 | -0,134979997 |
| HECTD3 | -0,134320304 |
| SREK1 | -0,133938199 |
| ADD1 | -0,133736545 |
| HNRNPA2B1 | -0,133331801 |
| RUVBL2 | -0,133213204 |
| ABI1 | -0,133056218 |
| EIF3G | -0,13293562 |
| TOX4 | -0,130808666 |
| LSS | -0,130431113 |
| SSU72 | -0,130278485 |
| ID2 | -0,129976717 |
| BTF3 | -0,129947876 |
| CMPK1 | -0,129285557 |
| HYOU1 | -0,129230814 |
| MYC | -0,128760318 |
| PAF1 | -0,128226704 |
| RHOF | -0,128134403 |
| FOXK2 | -0,127348052 |
| SAFB2 | -0,126002232 |
| CARM1 | -0,125934594 |
| GPR183 | -0,12585554 |
| WAC | -0,125664938 |
| POLR2A | -0,12484639 |
| PPIG | -0,124755187 |
| TNIP1 | -0,124063993 |
| MED15 | -0,12394026 |
| SRSF11 | -0,123456581 |
| CHERP | -0,12273624 |
| TNFAIP3 | -0,122732615 |
| DVL3 | -0,122655778 |
| DCAF5 | -0,122450895 |

| | |
|---|---|
| NDE1 | -0,121933528 |
| PPP1R9B | -0,121114438 |
| SUPT5H | -0,120859632 |
| TAPBP | -0,120595131 |
| ATF6B | -0,120167011 |
| TCF25 | -0,120150677 |
| SFSWAP | -0,118232829 |
| PROSER1 | -0,118066429 |
| CSNK2B | -0,117179444 |
| KMT2E | -0,114821024 |
| MGEA5 | -0,112181532 |
| FADS2 | -0,112078193 |
| ATF7IP | -0,108566469 |
| YBX3 | -0,108369879 |
| FNBP1 | -0,107791741 |
| RPL28 | -0,104699631 |
| BPTF | -0,10422342 |
| PRRC2C | -0,097153519 |
| RPS26 | -0,095561442 |

Up-regulated

| Gene | log2FoldChange |
|---|---|
| RPL3 | 0,094952231 |
| NACA | 0,09561793 |
| RPL4 | 0,095906604 |
| HLA-DRA | 0,09641374 |
| CAPZA1 | 0,100246307 |
| PCM1 | 0,100840676 |
| SEPT9 | 0,10267629 |
| IGF2R | 0,102946263 |
| ETS1 | 0,103439076 |
| ARPC5 | 0,103501774 |
| ANXA6 | 0,10409322 |
| H2AFZ | 0,10420576 |
| MTHFD1 | 0,104570951 |
| STARD7 | 0,105192137 |
| PMAIP1 | 0,106191677 |
| RPS28 | 0,106462924 |
| NEK6 | 0,107664391 |
| HLA-DQB1 | 0,107736438 |
| FBL | 0,108020305 |
| LPCAT1 | 0,108404243 |
| CD70 | 0,109392762 |
| CFL1 | 0,109623805 |

| | |
|---|---|
| GOT2 | 0,110369947 |
| SRSF3 | 0,111143038 |
| CTSC | 0,111317134 |
| STMN1 | 0,111356949 |
| RPS21 | 0,111497438 |
| SNX25 | 0,1117727 |
| H2AFX | 0,112230782 |
| MARS | 0,112634863 |
| ADRBK1 | 0,112653395 |
| PTDSS1 | 0,113573746 |
| GTF2I | 0,11487447 |
| MTDH | 0,115091759 |
| GSTP1 | 0,115490974 |
| MTHFD2 | 0,115646884 |
| ATP5A1 | 0,115739738 |
| ANAPC1 | 0,115996078 |
| LIMD2 | 0,117706815 |
| IDH3A | 0,118220601 |
| BCAT1 | 0,118221546 |
| VARS | 0,118239489 |
| SETX | 0,119366347 |
| LRPPRC | 0,119904431 |
| YARS | 0,119964336 |
| RANBP1 | 0,120126096 |
| CD52 | 0,120152944 |
| GALNT2 | 0,120394712 |
| RBBP4 | 0,12075484 |
| SHMT2 | 0,120786935 |
| RPS13 | 0,120857621 |
| TXNDC11 | 0,121265358 |
| MCM5 | 0,121523239 |
| HLA-DQA1 | 0,121730824 |
| SELT | 0,121840149 |
| PAFAH1B1 | 0,122117097 |
| CD79B | 0,122183054 |
| LYPLA1 | 0,122200111 |
| DDX39A | 0,122503785 |
| LGALS9 | 0,123054016 |
| BUB3 | 0,123436907 |
| RRM2B | 0,123874946 |
| CD53 | 0,124040459 |
| TOMM20 | 0,124061149 |
| NCAPD2 | 0,124384616 |
| MID1IP1 | 0,12454649 |
| CD164 | 0,126138454 |
| SPAG5 | 0,126210868 |

| | |
|---|---|
| RSL24D1 | 0,126228662 |
| USP15 | 0,127231759 |
| SEL1L | 0,127285895 |
| POLD1 | 0,127360113 |
| RPN2 | 0,127802307 |
| PHGDH | 0,128012749 |
| CTDSP1 | 0,128259067 |
| PCNT | 0,128268465 |
| BUB1 | 0,128623119 |
| CLTC | 0,12872567 |
| STT3B | 0,128930454 |
| GM2A | 0,129003365 |
| SORL1 | 0,129532988 |
| FKBP1A | 0,129645322 |
| CDK2 | 0,129693713 |
| CBL | 0,12970225 |
| STAT1 | 0,129838589 |
| PGAM5 | 0,129926928 |
| CHCHD2 | 0,130120596 |
| RRM2 | 0,130178394 |
| CDV3 | 0,130554199 |
| LMNB1 | 0,13059168 |
| WHSC1 | 0,130749073 |
| AKNA | 0,130797118 |
| NMD3 | 0,130857201 |
| SLC5A3 | 0,131054227 |
| AURKB | 0,131677239 |
| MDFIC | 0,132058354 |
| FAM78A | 0,132532955 |
| SBF1 | 0,132583828 |
| DHTKD1 | 0,132667507 |
| ATAD2 | 0,132819627 |
| RAD21 | 0,132870533 |
| SEP15 | 0,132931995 |
| ECHS1 | 0,133300901 |
| BUB1B | 0,134218381 |
| NAA25 | 0,134332932 |
| IPO5 | 0,134590902 |
| PRDX3 | 0,13491449 |
| GANAB | 0,134939484 |
| TES | 0,135081978 |
| SLC38A1 | 0,135118071 |
| TBL1XR1 | 0,135266924 |
| ZNF106 | 0,135812855 |
| RNH1 | 0,135816064 |
| PRKDC | 0,136251661 |

| | |
|---|---|
| CDCA7 | 0,136496626 |
| FERMT3 | 0,136861605 |
| RIC1 | 0,136920697 |
| FLNA | 0,137332366 |
| SLC1A5 | 0,137432693 |
| PLEKHB2 | 0,137479763 |
| SLC1A4 | 0,137542669 |
| ATR | 0,138162808 |
| RPS6KA3 | 0,138203706 |
| CCNB2 | 0,138386966 |
| ASPM | 0,138394996 |
| ZFAND5 | 0,138516094 |
| UCP2 | 0,13860577 |
| PLEKHO1 | 0,138644222 |
| POLR1B | 0,138874718 |
| MARCH6 | 0,138893398 |
| COX7C | 0,140262895 |
| TIMM13 | 0,140398629 |
| NREP | 0,140790001 |
| STARD4 | 0,141397563 |
| RPSA | 0,141686593 |
| RPL10A | 0,141763076 |
| PSMB3 | 0,141834649 |
| CAND1 | 0,1421379 |
| ORAI2 | 0,142254436 |
| RPL15 | 0,142278104 |
| TCF4 | 0,142337238 |
| NADK | 0,142575398 |
| PGK1 | 0,142789757 |
| BTBD2 | 0,142895076 |
| SPG21 | 0,142982203 |
| MAN1A1 | 0,143031146 |
| CCM2 | 0,143508496 |
| FAM49B | 0,143544117 |
| GLDC | 0,143657305 |
| CST3 | 0,143859801 |
| KDM4B | 0,14399739 |
| NNT | 0,144295786 |
| DOCK11 | 0,144454609 |
| MRPL35 | 0,144565802 |
| GPC4 | 0,144747687 |
| OAS1 | 0,144834978 |
| ACAP1 | 0,144968695 |
| SYNE1 | 0,145517577 |
| RB1 | 0,145521852 |
| PLA1A | 0,145787663 |

| | |
|---|---|
| NUP205 | 0,145867998 |
| TMEM30A | 0,145928297 |
| ASNS | 0,146072052 |
| VRK2 | 0,146746561 |
| IKZF1 | 0,146798602 |
| ANXA11 | 0,14682584 |
| ATP6V0E1 | 0,147199172 |
| DDIT4 | 0,14730018 |
| CXXC5 | 0,147513561 |
| STT3A | 0,147739804 |
| RAP1A | 0,147838131 |
| ATXN7L3 | 0,147921073 |
| CKS1B | 0,147971764 |
| ZBTB1 | 0,148046062 |
| ACADM | 0,148294013 |
| AKAP13 | 0,148548985 |
| EIF2AK3 | 0,148587474 |
| HPS5 | 0,148629671 |
| TOMM40 | 0,148787785 |
| KIFC1 | 0,149148919 |
| NDUFS7 | 0,14921218 |
| TACC1 | 0,149257563 |
| MCM4 | 0,149784508 |
| CLIC1 | 0,149813684 |
| PI4K2B | 0,149827584 |
| RAPGEF6 | 0,150109912 |
| KIF23 | 0,150365977 |
| UTP18 | 0,15052113 |
| ACAA2 | 0,150648248 |
| NDUFA9 | 0,150793389 |
| SPTY2D1 | 0,15081674 |
| GLRX | 0,1510406 |
| MMD | 0,151169077 |
| PAN3 | 0,151170486 |
| TEC | 0,151173521 |
| NCAPH | 0,151319826 |
| HLA-DRB1 | 0,1513859 |
| LRRK1 | 0,151749539 |
| SDHC | 0,151800602 |
| NCAPG2 | 0,151932005 |
| SAPCD2 | 0,15201064 |
| SLC12A2 | 0,152062441 |
| MYLIP | 0,152327589 |
| IFRD2 | 0,152534394 |
| TMX4 | 0,152556687 |
| PSMB8 | 0,152761533 |

| | |
|---|---|
| HIST1H1C | 0,15284023 |
| ADPRH | 0,153101654 |
| CYBA | 0,153809953 |
| ATP5B | 0,153872688 |
| ARSB | 0,154409537 |
| GSTO1 | 0,154411333 |
| E2F8 | 0,154433232 |
| SLC2A4RG | 0,154656282 |
| LONP1 | 0,155173595 |
| GINS4 | 0,155332492 |
| DUT | 0,155471496 |
| FANCD2 | 0,155638132 |
| SETDB2 | 0,155697799 |
| AKAP11 | 0,156350664 |
| SLAMF7 | 0,156570775 |
| RHOC | 0,156605971 |
| SYPL1 | 0,156652068 |
| MDH1 | 0,156835579 |
| FOXRED1 | 0,15693496 |
| GMFB | 0,157215856 |
| CDC25A | 0,157249745 |
| EARS2 | 0,157566649 |
| ZBTB2 | 0,158050221 |
| PLS3 | 0,158194495 |
| MCM2 | 0,158323337 |
| PICALM | 0,159199048 |
| DDX60 | 0,15936 |
| LPIN1 | 0,159433035 |
| CECR1 | 0,159720532 |
| PTPN7 | 0,159897002 |
| GMPR2 | 0,159936886 |
| SDHA | 0,16027726 |
| AURKAIP1 | 0,160598272 |
| DAD1 | 0,160665856 |
| NT5C3A | 0,161098845 |
| SLC20A1 | 0,161160727 |
| NR3C1 | 0,16136809 |
| COG4 | 0,161850235 |
| PHF19 | 0,16239361 |
| DPY19L3 | 0,162473292 |
| KCNAB2 | 0,162772659 |
| CARHSP1 | 0,162896498 |
| TBC1D2B | 0,163069143 |
| RASAL3 | 0,163399042 |
| FOXN2 | 0,163514542 |
| SAMD9L | 0,163612017 |

| | |
|---|---|
| EMB | 0,164056407 |
| AARS | 0,164239979 |
| CDC42SE2 | 0,164392742 |
| CENPN | 0,164601003 |
| COX15 | 0,165285345 |
| POLE | 0,165330486 |
| ZMYND8 | 0,165701746 |
| SIPA1 | 0,165815259 |
| ARPC4 | 0,16632706 |
| EMX1 | 0,166331528 |
| WDR36 | 0,167201698 |
| IRF7 | 0,167223741 |
| ITM2B | 0,167320766 |
| B3GNT2 | 0,167322638 |
| KIAA0101 | 0,167469413 |
| ATP5D | 0,167705603 |
| SUSD6 | 0,167851539 |
| SEPHS1 | 0,168146108 |
| XRN1 | 0,168956012 |
| CCNB1 | 0,169268278 |
| NUP85 | 0,170059118 |
| NDC1 | 0,170706642 |
| TIMELESS | 0,170766773 |
| TNFRSF13B | 0,170924999 |
| PSMA2 | 0,171060901 |
| ANKRD28 | 0,171190804 |
| UBE2S | 0,171215631 |
| TK1 | 0,171320211 |
| PLEKHA2 | 0,17210752 |
| CXCR4 | 0,172447417 |
| LY6E | 0,172466524 |
| JUN | 0,172780195 |
| DACT1 | 0,172980414 |
| SOX5 | 0,173615961 |
| FAM214A | 0,173759584 |
| ITGB7 | 0,173857209 |
| ATP8A1 | 0,174071353 |
| SYNE3 | 0,174085869 |
| SLC3A2 | 0,174592448 |
| RFX3 | 0,174802665 |
| UTRN | 0,174804208 |
| PCK2 | 0,175061851 |
| PMVK | 0,175214641 |
| LRRK2 | 0,175248272 |
| SUMF2 | 0,175253702 |
| FAM46A | 0,17530269 |

| | |
|---|---|
| MBNL3 | 0,175311739 |
| NCOA3 | 0,175473041 |
| FANCI | 0,175603444 |
| IFIT1 | 0,175620698 |
| SGOL2 | 0,176185068 |
| NUP155 | 0,17620098 |
| VPS51 | 0,176265224 |
| ITGA4 | 0,17645989 |
| FASTKD2 | 0,176821719 |
| ABCB10 | 0,177600188 |
| PTCD3 | 0,177852545 |
| RNF213 | 0,178383577 |
| NCF1 | 0,178654773 |
| STK11IP | 0,178740917 |
| MRPS28 | 0,178821686 |
| HOOK1 | 0,179459886 |
| KAT8 | 0,179659406 |
| ACTR1B | 0,179887941 |
| DCLRE1A | 0,180223907 |
| MIPEP | 0,180762306 |
| CD19 | 0,180894637 |
| C11orf31 | 0,181131455 |
| SFMBT2 | 0,181196229 |
| RNASEH2A | 0,182119998 |
| APOL6 | 0,182502585 |
| ORC1 | 0,182592204 |
| AHNAK | 0,183220036 |
| CHDH | 0,183313136 |
| MYB | 0,18402364 |
| CYB561A3 | 0,184823728 |
| NFE2L3 | 0,184835146 |
| GPD2 | 0,18486461 |
| DTYMK | 0,185326885 |
| USP1 | 0,185732316 |
| FGFR1 | 0,186262464 |
| PLXDC2 | 0,186331466 |
| SOGA1 | 0,18661116 |
| MMP7 | 0,186976354 |
| WDFY1 | 0,187515414 |
| ALOX5 | 0,188326679 |
| PHPT1 | 0,188959172 |
| ARHGAP6 | 0,189680071 |
| MMS22L | 0,190103115 |
| PTAR1 | 0,19037221 |
| S1PR2 | 0,191127022 |
| SCARB2 | 0,191154511 |

| | |
|---|---|
| DOK1 | 0,191655899 |
| RGS19 | 0,191676512 |
| UBL4A | 0,191702436 |
| ISG15 | 0,191851874 |
| OSTC | 0,192475333 |
| IKZF2 | 0,19254017 |
| ATL2 | 0,192990677 |
| GK | 0,193262498 |
| RNGTT | 0,193445397 |
| TNFRSF1B | 0,194262184 |
| SLAMF6 | 0,194273558 |
| MDM4 | 0,194453883 |
| CLN6 | 0,194627283 |
| ADCY7 | 0,195795201 |
| RPSAP58 | 0,196132206 |
| COX18 | 0,196260585 |
| CDKN3 | 0,196333273 |
| SLC19A1 | 0,196926214 |
| ATP1B3 | 0,19748034 |
| SOX4 | 0,197503479 |
| FAM43A | 0,197660744 |
| DFNA5 | 0,197802193 |
| LZTS2 | 0,198108955 |
| DUS3L | 0,198249357 |
| ALDH16A1 | 0,199188594 |
| THOC3 | 0,199281167 |
| NME1 | 0,199410001 |
| MRPS18B | 0,199576327 |
| BMF | 0,199632172 |
| CENPV | 0,199746071 |
| FCRLA | 0,19983588 |
| TEP1 | 0,200473677 |
| BRCA2 | 0,201520617 |
| SLC7A11 | 0,201806569 |
| LIMA1 | 0,201849793 |
| MRPL1 | 0,20278121 |
| COL19A1 | 0,202812926 |
| FIZ1 | 0,20369808 |
| PNP | 0,204077056 |
| MPEG1 | 0,204305684 |
| COQ2 | 0,204476091 |
| SIVA1 | 0,204588029 |
| ABCA6 | 0,205413893 |
| MACC1 | 0,205980952 |
| MYO1G | 0,206012022 |
| TEX9 | 0,206484 |

| | |
|---|---|
| MTX1 | 0,207089897 |
| NLRX1 | 0,207384454 |
| TNFRSF21 | 0,207715798 |
| ZWILCH | 0,207887493 |
| CA2 | 0,208175919 |
| CSGALNACT2 | 0,20973165 |
| MRPL23 | 0,210403591 |
| CLCN5 | 0,211282915 |
| SLC35E2B | 0,21140753 |
| ISOC2 | 0,212669546 |
| RICTOR | 0,212695669 |
| MAD2L1BP | 0,212752066 |
| KIAA1671 | 0,214213039 |
| ANK1 | 0,214445657 |
| ARHGAP4 | 0,214594253 |
| CNR2 | 0,214719022 |
| MRPS36 | 0,215108778 |
| JTB | 0,215238748 |
| GPCPD1 | 0,215468414 |
| HSPB1 | 0,215574265 |
| WDR83OS | 0,215895948 |
| BZRAP1-AS1 | 0,216821755 |
| SEMA4D | 0,217725207 |
| HLA-DMB | 0,218150865 |
| EDRF1 | 0,219529819 |
| ZFAND4 | 0,22109401 |
| SERPINB9P1 | 0,221387056 |
| GIMAP2 | 0,221787869 |
| LY9 | 0,223053447 |
| LOC101927027 | 0,223294094 |
| PTP4A3 | 0,22492656 |
| FBXO41 | 0,226336917 |
| ENOSF1 | 0,226450605 |
| PSMB10 | 0,228936161 |
| GALNT7 | 0,230134726 |
| CHAC1 | 0,230524723 |
| FGR | 0,231913869 |
| TAF6L | 0,232597883 |
| PXMP2 | 0,233452064 |
| SLC25A53 | 0,234020878 |
| CD84 | 0,2343549 |
| KIF21B | 0,235314051 |
| TRAF3IP3 | 0,235377191 |
| MRPL54 | 0,235604186 |
| PRDM15 | 0,237847702 |
| MAP2K6 | 0,237860539 |

| | |
|---|---|
| SLC15A4 | 0,237885684 |
| PSAT1 | 0,239097381 |
| TRIP6 | 0,239283469 |
| GINS3 | 0,239805916 |
| CCDC109B | 0,240238683 |
| TMEM208 | 0,240601077 |
| MZB1 | 0,241495001 |
| FAH | 0,241660492 |
| IL3RA | 0,241853296 |
| ADAMTS7 | 0,242253087 |
| ITGB8 | 0,243369649 |
| APOBEC3B | 0,243979299 |
| TMEM140 | 0,243993973 |
| SPATA13 | 0,245649188 |
| TMEM160 | 0,246887555 |
| LRRC61 | 0,247689187 |
| MYRIP | 0,248740957 |
| DGKD | 0,248852765 |
| C14orf159 | 0,24904761 |
| RASSF6 | 0,250332512 |
| UGGT2 | 0,250596905 |
| FAM64A | 0,250931375 |
| ZNF611 | 0,25130264 |
| SETD4 | 0,252243772 |
| C3orf58 | 0,253205455 |
| DDX60L | 0,254073722 |
| IL15 | 0,256070144 |
| CSRNP1 | 0,256070578 |
| PP7080 | 0,257156596 |
| TMC8 | 0,260073068 |
| MACROD2 | 0,261771706 |
| TMEM62 | 0,262832812 |
| SLC38A5 | 0,262946387 |
| SPARC | 0,263967546 |
| IL32 | 0,266563657 |
| TNFSF11 | 0,268626821 |
| UGT2B17 | 0,268741287 |
| C19orf52 | 0,268882758 |
| ACSM3 | 0,269052811 |
| FCMR | 0,269348318 |
| ZNF273 | 0,270210224 |
| TNFRSF11A | 0,270336321 |
| ITGAL | 0,271965247 |
| C10orf128 | 0,273318261 |
| ABHD11 | 0,274309835 |
| SLC12A8 | 0,275390493 |

| | |
|---|---|
| ZNF75D | 0,276243541 |
| CACNB1 | 0,278097165 |
| GHDC | 0,280163045 |
| EIF5AL1 | 0,281160875 |
| FAM83D | 0,28155372 |
| KMO | 0,281838366 |
| D2HGDH | 0,282401017 |
| NCF1B | 0,28268132 |
| AICDA | 0,282941023 |
| TNFRSF17 | 0,283094031 |
| ALPK2 | 0,283108527 |
| S1PR4 | 0,285335051 |
| DDR2 | 0,28549671 |
| LRIG3 | 0,288176787 |
| MED18 | 0,295930405 |
| VDR | 0,29636843 |
| CD27 | 0,296710568 |
| ARHGAP9 | 0,297333555 |
| CCR10 | 0,302633356 |
| LIPH | 0,302796142 |
| MPZL3 | 0,302859715 |
| ADCK5 | 0,305473252 |
| RHOB | 0,306612999 |
| HS3ST1 | 0,306825959 |
| C1orf106 | 0,308011125 |
| KCNA3 | 0,309234571 |
| SLC39A10 | 0,310032858 |
| TREML2 | 0,311098454 |
| YDJC | 0,313629132 |
| H3F3AP4 | 0,314293402 |
| TLR9 | 0,314325928 |
| KLK1 | 0,315779948 |
| CFAP57 | 0,320009131 |
| CACNA1E | 0,323559991 |
| FUOM | 0,326551908 |
| CALCRL | 0,328546766 |
| FJX1 | 0,333398616 |
| NCKAP5 | 0,33393549 |
| SLITRK6 | 0,334516131 |
| MS4A7 | 0,338258476 |
| ENPP2 | 0,340092592 |
| HLA-DOB | 0,343846182 |
| ASS1 | 0,347798422 |
| TMEM86B | 0,34853837 |
| ITGA11 | 0,349451482 |
| CACNA1D | 0,350477148 |

| | |
|---|---|
| CD24 | 0,350495029 |
| CCR6 | 0,35193935 |
| TFAP2B | 0,353202518 |
| NCAM2 | 0,357308415 |
| CD200R1 | 0,363858682 |
| SLCO4A1 | 0,364552851 |
| APELA | 0,369120344 |
| CXCL10 | 0,369868324 |
| NOD2 | 0,373751133 |
| TNFSF15 | 0,377972921 |
| LINC01055 | 0,384425515 |
| LOC100507195 | 0,38551382 |
| CREB3L1 | 0,38624142 |
| RRN3P3 | 0,39128435 |
| B3GLCT | 0,391994796 |
| EPHA4 | 0,393988743 |
| CSPG4 | 0,398646647 |
| DPYD | 0,404107075 |
| ASB9 | 0,415098026 |
| SAMSN1 | 0,41619098 |
| S100A4 | 0,416194743 |
| BTNL9 | 0,418723728 |
| LINC00996 | 0,423670352 |
| CD101 | 0,428875128 |
| FGFR4 | 0,440268017 |
| LINC00426 | 0,442358831 |
| BZW2 | 0,444424392 |
| RNASE6 | 0,445068764 |
| KCNIP2 | 0,447014816 |
| LINC01551 | 0,448804259 |
| GAS2 | 0,464225615 |
| SFN | 0,465652579 |
| GPR174 | 0,469021863 |
| CORO2B | 0,469791654 |
| FRMD4A | 0,480824747 |
| EVI2A | 0,48906891 |
| INHBE | 0,491059985 |
| TPRG1 | 0,491222641 |
| OTOGL | 0,491853806 |
| CLNK | 0,493473034 |
| TIAM2 | 0,494388312 |
| RFX3-AS1 | 0,500890338 |
| SGMS1-AS1 | 0,503150776 |
| CD28 | 0,507427629 |
| TGM2 | 0,511527564 |
| FCGBP | 0,513548078 |

| | |
|---|---|
| MYO5C | 0,519026454 |
| CMKLR1 | 0,526639812 |
| GRAP2 | 0,52723991 |
| FGL2 | 0,52809182 |
| PRLR | 0,531522108 |
| DOK2 | 0,532473927 |
| SMG1P3 | 0,539625388 |
| VWA1 | 0,542395418 |
| PCDHGC3 | 0,545010851 |
| MYH11 | 0,54615443 |
| ADGRD1 | 0,546476592 |
| SIX4 | 0,54675682 |
| NEURL3 | 0,552418326 |
| CTTNBP2 | 0,55554532 |
| TDO2 | 0,555770761 |
| CLDN1 | 0,561501384 |
| HES2 | 0,573002198 |
| PTPN13 | 0,576357624 |
| LOXL3 | 0,576695127 |
| GPR18 | 0,581032819 |
| LOC101929450 | 0,581462748 |
| RASGRP2 | 0,583749777 |
| CEACAM1 | 0,584147889 |
| PKP2 | 0,585944279 |
| CFAP54 | 0,587077975 |
| AMZ1 | 0,591580884 |
| MYOF | 0,592861327 |
| PVRIG | 0,593284511 |
| MIR1282 | 0,598491019 |
| GNAZ | 0,599097131 |
| LINC01226 | 0,610621644 |
| ZBED2 | 0,621561272 |
| LOC100128164 | 0,634511027 |
| IL21R-AS1 | 0,634688354 |
| PADI2 | 0,637779764 |
| UGT2A3 | 0,645345442 |
| CXCL8 | 0,663747359 |
| EBLN2 | 0,682903004 |
| LGALS14 | 0,687187143 |
| LOC100130093 | 0,704783347 |
| CHST9 | 0,713731434 |
| CCL25 | 0,732991288 |
| NINJ2 | 0,769009986 |
| DOK7 | 0,770174857 |
| FSD2 | 0,787083787 |
| F2RL3 | 0,792281859 |

| | |
|---|---|
| NEUROD2 | 0,800873819 |
| SLFNL1-AS1 | 0,80847723 |
| TMEM119 | 0,832893717 |
| LOC728989 | 0,872768812 |
| IGF1 | 0,92550886 |
| CDKN1C | 0,940395985 |
| NFATC4 | 0,969091963 |
| LOC100505530 | 0,996094612 |
| SEPP1 | 1,01104E+14 |
| PRC1-AS1 | 1,03715E+14 |
| MIR1268A | 1,04096E+14 |
| PDZRN3 | 1,04536E+14 |
| GPRC5C | 1,07297E+14 |
| TCL6 | 1,08758E+14 |
| NCR2 | 1,16967E+14 |
| C8orf89 | 1,42992E+14 |
| GYLTL1B | 1,57438E+14 |
| FAM69B | 1,71583E+14 |
| HK3 | 1,72777E+14 |
| GTF2H2C | 1,82611E+14 |
| SNORA72 | 1,96424E+14 |
| THBS1 | 2,41631E+14 |
| MIR378J | 3,52522E+14 |
| C18orf61 | 5,40073E+14 |
